# Improving risk indexes for Alzheimer’s disease and related dementias for use in midlife

**DOI:** 10.1093/braincomms/fcac223

**Published:** 2022-10-06

**Authors:** Aaron Reuben, Terrie E Moffitt, Wickliffe C Abraham, Antony Ambler, Maxwell L Elliott, Ahmad R Hariri, Honalee Harrington, Sean Hogan, Renate M Houts, David Ireland, Annchen R Knodt, Joan Leung, Amber Pearson, Richie Poulton, Suzanne C Purdy, Sandhya Ramrakha, Line J H Rasmussen, Karen Sugden, Peter R Thorne, Benjamin Williams, Graham Wilson, Avshalom Caspi

**Affiliations:** Department of Psychology and Neuroscience, Duke University, Durham, NC, USA; Department of Psychology and Neuroscience, Duke University, Durham, NC, USA; Center for Genomic and Computational Biology, Duke University, Durham, NC, USA; Department of Psychiatry and Behavioral Sciences, Duke University, Durham, NC, USA; King’s College London, Social, Genetic, and Developmental Psychiatry Centre, Institute of Psychiatry, Psychology, and Neuroscience, London, UK; PROMENTA, Department of Psychology, University of Oslo, Oslo, Norway; Brain Health Research Centre, Department of Psychology, University of Otago, Dunedin, New Zealand; King’s College London, Social, Genetic, and Developmental Psychiatry Centre, Institute of Psychiatry, Psychology, and Neuroscience, London, UK; Department of Psychology and Neuroscience, Duke University, Durham, NC, USA; Department of Psychology and Neuroscience, Duke University, Durham, NC, USA; Department of Psychology and Neuroscience, Duke University, Durham, NC, USA; Dunedin Multidisciplinary Health and Development Research Unit, Department of Psychology, University of Otago, Dunedin, New Zealand; Department of Psychology and Neuroscience, Duke University, Durham, NC, USA; Dunedin Multidisciplinary Health and Development Research Unit, Department of Psychology, University of Otago, Dunedin, New Zealand; Department of Psychology and Neuroscience, Duke University, Durham, NC, USA; School of Psychology, The University of Auckland, Auckland, New Zealand; Department of Geography, Environment, and Spatial Sciences, Michigan State University, East Lansing, MI, USA; Department of Public Health, University of Otago, Wellington, New Zealand; Dunedin Multidisciplinary Health and Development Research Unit, Department of Psychology, University of Otago, Dunedin, New Zealand; Center for Brain Research, Faculty of Medical and Health Sciences, The University of Auckland, Auckland, New Zealand; Dunedin Multidisciplinary Health and Development Research Unit, Department of Psychology, University of Otago, Dunedin, New Zealand; Department of Clinical Research, Copenhagen University Hospital Amager and Hvidovre, Hvidovre, Denmark; Department of Psychology and Neuroscience, Duke University, Durham, NC, USA; Center for Brain Research, Faculty of Medical and Health Sciences, The University of Auckland, Auckland, New Zealand; Faculty of Medical and Health Sciences, Department of Physiology, The University of Auckland, Auckland, New Zealand; Section of Audiology, Faculty of Medical and Health Sciences, The University of Auckland, Auckland, New Zealand; Department of Psychology and Neuroscience, Duke University, Durham, NC, USA; Dunedin Multidisciplinary Health and Development Research Unit, Department of Psychology, University of Otago, Dunedin, New Zealand; Department of Preventive and Social Medicine, Dunedin School of Medicine, University of Otago, Dunedin, New Zealand; Department of Psychology and Neuroscience, Duke University, Durham, NC, USA; Center for Genomic and Computational Biology, Duke University, Durham, NC, USA; Department of Psychiatry and Behavioral Sciences, Duke University, Durham, NC, USA; King’s College London, Social, Genetic, and Developmental Psychiatry Centre, Institute of Psychiatry, Psychology, and Neuroscience, London, UK; PROMENTA, Department of Psychology, University of Oslo, Oslo, Norway

**Keywords:** Alzheimer’s disease, dementia, risk index, preventive medicine, modifiable risk factors

## Abstract

Knowledge of a person’s risk for Alzheimer’s disease and related dementias (ADRDs) is required to triage candidates for preventive interventions, surveillance, and treatment trials. ADRD risk indexes exist for this purpose, but each includes only a subset of known risk factors. Information missing from published indexes could improve risk prediction. In the Dunedin Study of a population-representative New Zealand-based birth cohort followed to midlife (*N* = 938, 49.5% female), we compared associations of four leading risk indexes with midlife antecedents of ADRD against a novel benchmark index comprised of nearly all known ADRD risk factors, the Dunedin ADRD Risk Benchmark (DunedinARB). Existing indexes included the Cardiovascular Risk Factors, Aging, and Dementia index (CAIDE), LIfestyle for BRAin health index (LIBRA), Australian National University Alzheimer’s Disease Risk Index (ANU-ADRI), and risks selected by the Lancet Commission on Dementia. The Dunedin benchmark was comprised of 48 separate indicators of risk organized into 10 conceptually distinct risk domains. Midlife antecedents of ADRD treated as outcome measures included age-45 measures of brain structural integrity [magnetic resonance imaging-assessed: (i) machine-learning-algorithm-estimated brain age, (ii) log-transformed volume of white matter hyperintensities, and (iii) mean grey matter volume of the hippocampus] and measures of brain functional integrity [(i) objective cognitive function assessed via the Wechsler Adult Intelligence Scale-IV, (ii) subjective problems in everyday cognitive function, and (iii) objective cognitive decline measured as residualized change in cognitive scores from childhood to midlife on matched Weschler Intelligence scales]. All indexes were quantitatively distributed and proved informative about midlife antecedents of ADRD, including algorithm-estimated brain age (*β*'s from 0.16 to 0.22), white matter hyperintensities volume (*β*'s from 0.16 to 0.19), hippocampal volume (*β*'s from −0.08 to −0.11), tested cognitive deficits (*β*'s from −0.36 to −0.49), everyday cognitive problems (*β*'s from 0.14 to 0.38), and longitudinal cognitive decline (*β*'s from −0.18 to −0.26). Existing indexes compared favourably to the comprehensive benchmark in their association with the brain structural integrity measures but were outperformed in their association with the functional integrity measures, particularly subjective cognitive problems and tested cognitive decline. Results indicated that existing indexes could be improved with targeted additions, particularly of measures assessing socioeconomic status, physical and sensory function, epigenetic aging, and subjective overall health. Existing premorbid ADRD risk indexes perform well in identifying linear gradients of risk among members of the general population at midlife, even when they include only a small subset of potential risk factors. They could be improved, however, with targeted additions to more holistically capture the different facets of risk for this multiply determined, age-related disease.

## Teaser

Reuben *et al*. report that existing dementia-risk indexes are informative about brain health in the general population decades before dementia symptoms emerge, even when the indexes include only a subset of potential risks. Comparisons to a novel comprehensive risk-benchmark indicated that targeted risk-factor additions could improve the selection of at-risk individuals.

## Graphical Abstract

**Graphical abstract:**
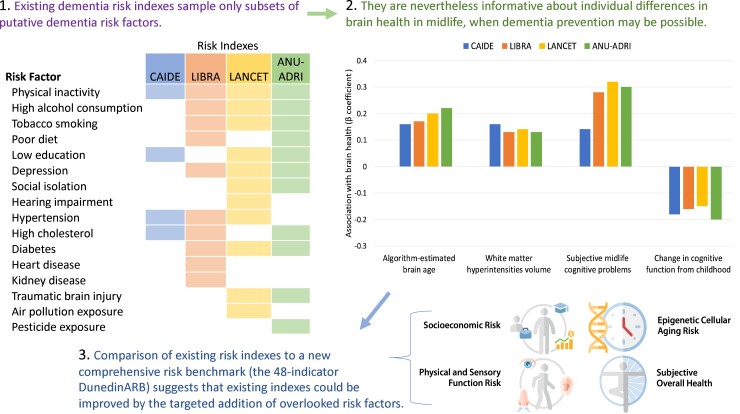

## Introduction

The population-burden of Alzheimer’s disease and related dementias (ADRD) is growing as the global population ages. Currently, 50 million people worldwide are estimated to have dementia, and that number is expected to triple within 30 years.^1^ As a non-specific consequence of diverse brain pathologies,^2^ dementia is multiply determined, with many potential paths leading to impairment that unfolds across years and, potentially, decades. As one consequence of the diversity of risk factors and long premorbid phase, most late-life individual ADRD interventions, including nearly all pharmaceuticals,^3^ have not yet been able to prevent disease, delay progression, or dramatically improve symptoms.

Attention is now turning to preventive efforts in midlife, both behavioural and pharmacological, that can decrease premorbid ADRD risk or else delay onset of impairment to extend patients’ functional years and lower the population-burden of disease.^4^ Such multimodal interventions with at least some effectiveness appear to be on the horizon,^5–7^ although not without controversy.^8^ Promising preventive ADRD therapies will generate new challenges for dementia study and care because they are likely to be: expensive, particularly when combined with surveillance neuroimaging; scarce, at least initially; and in high-demand from consumers, both those at-risk and the legions of worried-well.^5,8^ According to a recent analysis by Alzheimer’s Disease International of 70 000 survey respondents from 155 countries, 95% of the general public believe that they will develop dementia at some point in their lives, and most are concerned about it.^1^

In order to target scarce intervention resources to those individuals most in-need, improve selection of participants for long-term randomized controlled trials, and support the work of clinicians who will be called upon to screen and diagnose ADRD risk, a number of premorbid risk indexes have been developed for use in midlife.^9^ Each index follows different risk factor selection criteria. Some, including the LIfestyle for BRAin health (LIBRA) index,^10^ are comprised of only modifiable risk factors, such as physical activity and weight. Others, including the Australian National University Alzheimer’s Disease Risk Index (ANU-ADRI),^11^ are comprised of only risk factors that could be assessed via self-report, such as social engagement. Others still, including the Cardiovascular Risk Factors, Aging, and Dementia (CAIDE) index,^12^ are comprised of only risk factors that were available in a longitudinal test cohort. Although many of the published risk indexes have shown moderate longitudinal predictive validity,^10,13–16^ each is comprised of different risk factors that represent only a subset of the risks associated with ADRD—typically those that are convenient to measure in physicians’ clinics (e.g. hypertension) or on questionnaires (e.g. educational attainment). Because existing risk indexes sample only a part of the universe of known risk factors for dementia, it is not clear to what extent different indexes identify the same or different individuals as ‘at-risk’ or, further, to what extent important information may be missing from these algorithms that could improve risk prediction.

We have the good fortune to have prospective measures of nearly all putative ADRD risk factors in one population-representative birth cohort assessed repeatedly and followed to midlife: the New Zealand-based Dunedin Study. This allowed us to construct a comprehensive ADRD Risk Benchmark [hereafter Dunedin Alzheimer’s disease and related dementias Risk Benchmark (DunedinARB)] to evaluate the performance of existing ADRD risk indexes in terms of their associations with midlife measures of brain health that are known antecedents of ADRD. We designed the DunedinARB to include 48 separate risk indicators grouped into 10 conceptually distinct domains of risk: genetic (e.g. family history of dementia), lifestyle (e.g. tobacco and alcohol consumption), socioeconomic (e.g. low educational attainment), psychological and somatic (e.g. history of major depression), physical and sensory (e.g. hearing impairment), cardio-metabolic (e.g. hypertension), inflammatory [e.g. high C-reactive protein (CRP) levels], epigenetic (e.g. high scores on DNA methylation epigenetic aging clocks), harmful events [e.g. history of traumatic brain injury (TBI)], and overall health (e.g. poor self-appraised subjective health). Using this comprehensive benchmark, we set out to determine whether a risk index comprised of most ADRD risk factors would be significantly more strongly associated with brain health at midlife compared with more limited indexes and, by extension, identify gaps in existing indexes. Importantly, we are not recommending this benchmark for clinical use; rather we developed it for comparative analyses here to reveal whether there is any information missing from published risk indexes that might be worth the extra effort to collect.

In addition to the DunedinARB, we generated measures of four top published ADRD risk indexes suitable for use in midlife: (i) the CAIDE; (ii) the LIBRA; (iii) an index comprised of the modifiable risk factors for dementia selected by the Lancet Commission on Dementia Prevention, Intervention, and Care identified based on the availability of systematic or meta-analytic review evidence and (iv) the ANU-ADRI. (Risk scores designed primarily for determining disease risk later in life, age 65+, have been tested elsewhere.)^17–20^ Dunedin Study member scores on the four published risk indexes plus the benchmark DunedinARB were then compared on their association with diverse measures of midlife brain structural and functional integrity that are known antecedents of ADRD,^21–25^ including magnetic resonance imaging (MRI)-measured brain aging and white matter disease, objective tests of cognitive function and decline, and subjective reports of everyday cognitive difficulties. Analyses were conducted in two sequential stages, beginning, first, with the construction and validation of the benchmark DunedinARB and moving, second, to the construction of the four published risk indexes and a comparison of their performance to each other and to the benchmark.

## Materials and methods

### Study design and population

Participants were members of the Dunedin Study. The full cohort comprises all individuals born between April 1972 and March 1973 in Dunedin, New Zealand, who were eligible based on residence in the province and who participated in the first assessment at age 3. The cohort represents the full range of socioeconomic status in the general population of New Zealand’s South Island.^26^ On adult health, the cohort matches the New Zealand National Health and Nutrition Survey on key indicators (e.g. body mass index, smoking, physical activity and visits to a physician)^26^ and the NZ Census of citizens of the same age on educational attainment.^27^ The cohort is primarily white (as self-described using fixed categories); 7.5% self-identify as being Maori, which matches the ethnic distribution of the South Island of New Zealand. Assessments were carried out at birth and ages 3, 5, 7, 9, 11, 13, 15, 18, 21, 26, 32, 38, and the most recent data collection was completed in April 2019, at age 45 years. Participants gave written informed consent, and Study protocols were approved by the Health and Disability Ethics Committee.

### Measures

We studied 48 ADRD risk indicators (organized into 10 risk domains) and 6 midlife measures of brain structural and functional integrity. These are described in detail in Supplementary Tables 1 and 2, respectively. All midlife outcome measures were assessed at age 45, when Study members completed standardized interviews, cognitive testing, and a neuroimaging MRI protocol including assessment of brain structure with T1-weighted, fluid-attenuated inversion recovery, and diffusion-weighted sequences using a Siemens Skyra 3T scanner (Siemens Healthcare) equipped with a 64-channel head and neck coil.

### Statistical analysis

**Figure 1:**
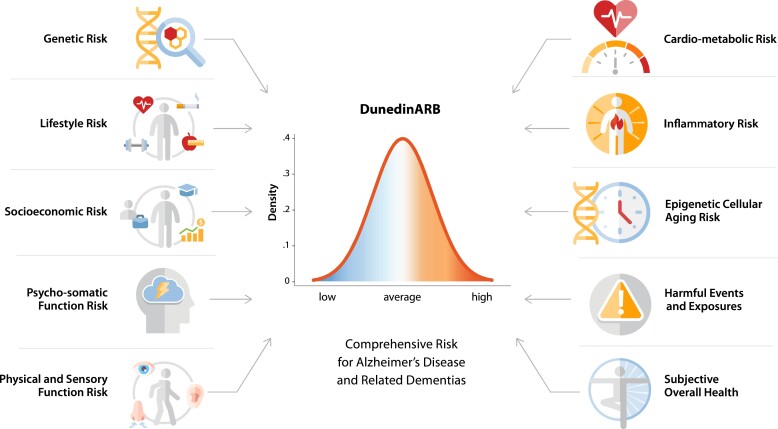
**Schematic of the DunedinARB.** The comprehensive DunedinARB is comprised of 48 risk indicators grouped into 10 conceptually distinct domains. Genetic risk includes family history of dementia and APOE ε4 allele status. Lifestyle risk includes physical activity, diet, tobacco smoking, alcohol consumption, folic acid supplementation, and regular prophylactic NSAID use. Socioeconomic risk includes occupational and educational attainment. Psycho-somatic Function risk includes chronic pain, history of migraine, history of depression, social isolation, sleep quality, neuroticism and conscientiousness. Physical and Sensory Function risk includes balance, gait, hearing acuity and subjective hearing function, objective and subjective vision function, and sense of smell. Cardio-Metabolic Status risk includes hypertension, obesity, and diabetes status, total cholesterol, triglycerides, and retinal vascular health. Inflammatory risk includes CRP, Interleukin 6, and soluble urokinase Plasminogen Activator Receptor levels and history of rheumatoid arthritis. Epigenetic Cellular Aging risk includes four separate DNA methylation ‘aging’ clocks (Horvath, Hannum, PhenoAge and GrimAge). Harmful Events and Exposures risk includes childhood lead exposure, occupational exposure to neurotoxicants and history of TBI. Subjective Overall Health risk includes self, informant and research-worker ratings of Study member overall health. Details on the individual risk factors and indicators are provided in Supplementary Table 1.

The study followed two stages. In the first stage, the benchmark DunedinARB (Fig. 1) was constructed following the criteria presented in Supplementary Table 1 and evaluated with respect to: (i) the intercorrelation of its component risk domains, (ii) its overall distribution and (iii) its sex-adjusted association, using ordinary least squares (OLS) regression, with the midlife measures of brain structural and functional integrity. The DunedinARB was generated for all Study members who attended the age-45 data collection, using Markov chain Monte Carlo imputation with multiple chains, in SAS, to impute missing risk indicator data. Attrition analysis (Supplementary Appendix1) using childhood IQ, childhood SES and Adverse Childhood Events, among other measures, identified no significant differences between the full cohort (*N* = 1037), those alive at age 45 (*N* = 997), and those who attended data collection (*N* = 938). Those who were deceased by the time of data collection had significantly lower childhood IQ’s than those who were still alive (*t* = 2.09, *P*-values = 0.04).

In the second stage, the four published risk indexes (the CAIDE, the LIBRA, the Lancet and the ANU-ADRI) were constructed following the criteria presented in Supplementary Table 3 and evaluated with respect to: (i) their correlation with each other and with the DunedinARB, (ii) their overall distributions and (iii) their sex-adjusted association, using OLS regression, with the midlife measures of brain structural and functional integrity. The published indexes were generated for all Study members who attended the age-45 assessment (*N* = 938).

Analyses were conducted using Stata v16.1 and SAS 9.4. Findings were checked for reproducibility by an independent data-analyst, who recreated the code based on the manuscript and applied it to a fresh dataset. This report follows the STROBE (STrengthening the Reporting of OBservational studies in Epidemiology) reporting guidelines for observational studies.^28^ Significance tests were two-tailed, *α* = 0.05. To minimize false positives, a false discovery rate correction^29^ was applied to all tests of the association of the ADRD risk indexes with the outcome measures of brain structural and functional integrity. Tests of the hippocampal-volume outcome measure were additionally adjusted for total brain volume in *post hoc* sensitivity tests.

### Data availability

The Dunedin Study datasets reported in the current article are available on request by qualified scientists. Requests require a concept paper describing the purpose of data access, ethical approval at the applicant’s university and provision for secure data access (https://moffittcaspi.trinity.duke.edu/research-topics/dunedin). We offer secure access on the Duke, Otago, and King’s College London campuses.

## Results

### Construction and validation of the benchmark DunedinARB

A comprehensive benchmark for midlife ADRD risk, the DunedinARB, was generated for each Study member who attended the age-45 assessment wave (*N* = 938; 90.5% of the original cohort, 94.1% of the original cohort members alive at age 45; 49.5% female). The DunedinARB was comprised of nearly all known or proposed risk factors for ADRD (Fig. 1), organized into 10 conceptually distinct domains of risk, each comprised of 2–7 distinct risk indicators (48 indicators in total). Established or proposed risk factors for ADRD were identified by non-systematic search of PubMed, Scopus, and Google Scholar for these five key words: Alzheimer’s, dementia, risk, review and meta-analysis. Additional risk factors were identified by review of the 2011 NIH State of the Science Consensus Panel on ADRD risk factors^30^ and review of selected factors in each of the four published risk indexes included in the second stage of our research. Supplementary Appendix 2 details the risk factor search methodology and Supplementary Table 1 describes the individual risk factors included in the DunedinARB, their missingness, assessment procedures and the assignment of risk points. Total risk scores in each of the 10 risk domains were *z*-scored and then summed to produce the benchmark DunedinARB with equal contributions from each domain.

**Figure 2:**
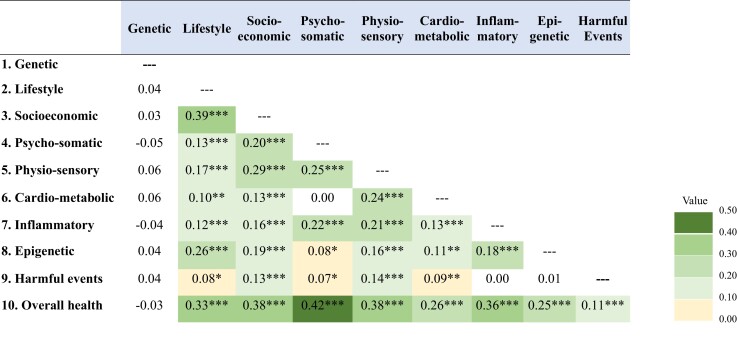
**Overlap (correlation, Pearson’s *r*) among the 10 domains of risk comprising the DunedinARB.** *P-values < 0.05, **P-values < 0.01, ***P-values < 0.001. Shading reflects association size for significant associations (*P* < 0.05), with darker colours highlighting larger associations. Risk domains are as follows: (i) genetic risk; (ii) lifestyle risk, (iii) socioeconomic risk; (iv) psycho-somatic function risk; (v) physical and sensory function risk; (vi) cardio-metabolic status risk; (vii) inflammatory risk; (viii) epigenetic cellular aging risk; (ix) harmful events and exposures risk; and (x) subjective overall health risk. Individual risk indicators contributing to each of the 10 risk domains are detailed in Supplementary Table 1; risk domains included between 2 and 7 indicators each.

Fig. 2 presents the overlap (correlation) among the 10 domains of risk comprising the DunedinARB. Overlap was common, such that no risk domain was entirely independent of the others. However, correlations tended to be small to moderate (Pearson’s *r* from −0.05 to 0.42), suggesting that each risk domain provided non-redundant information to the overall DunedinARB. The highest overlap among the conceptually distinct risk domains followed common-sense expectations, including socioeconomic status risk with lifestyle risk (*r* = 0.39, *P* < 0.001), psychological and somatic function risk with subjective overall health risk (*r* = 0.42, *P* < 0.001), and cardio-metabolic status risk with physical and sensory function risk (*r* = 0.24, *P* < 0.001). Subjective overall health risk correlated modestly with most other risk domains (*r* > 0.25 with seven out of nine risk domains). Supplementary Table 4 presents correlations among individual risk indicators within the 10 domains of risk, which varied by domain but tended to be small to moderate (*r* < 0.50).

**Figure 3:**
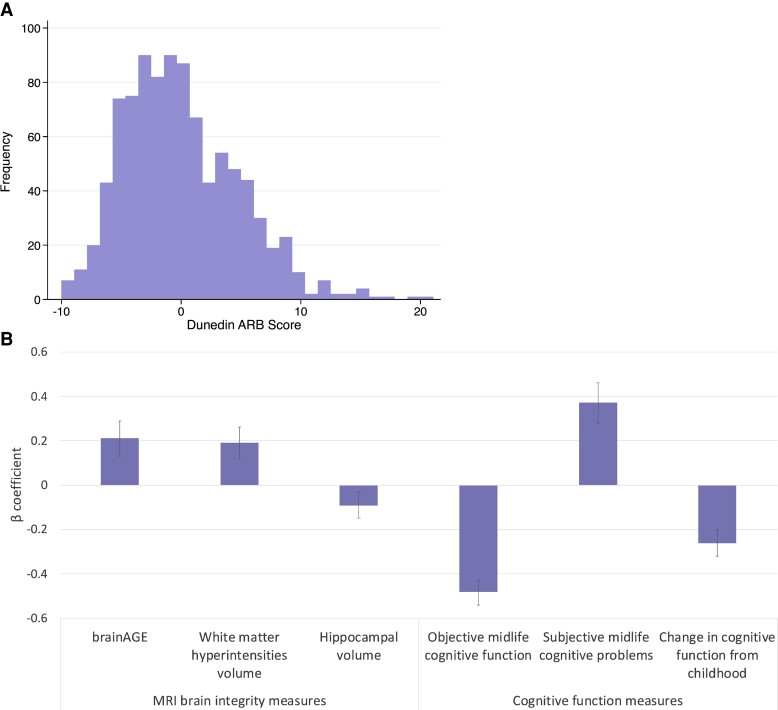
**Performance characteristics of the DunedinARB.** (**A**) Presents the distribution of DunedinARB scores in the cohort. (**B**) Presents the association of the DunedinARB with the midlife measures of brain structural (MRI) and functional (cognitive) integrity. *β*'s coefficients in (**B**) are derived from OLS regression of the brain integrity outcomes onto the DunedinARB, with adjustment for sex. Analytic sample sizes vary by outcome, from *N* = 852 (white matter hyperintensities volume) to *N* = 921 (subjective midlife cognitive problems).

Fig. 3(A) presents the distribution of the overall DunedinARB in the population-representative Dunedin Study cohort [mean (SD) = 0 (4.86)], which followed an approximately normal, if slightly leptokurtic, distribution in the cohort (skew = 0.72, kurtosis = 3.39). Some Study members had very high or very low risk, but most had low to moderate risk. Overall, men had greater risk than women [mean (SD) DunedinARB score = 0.58 (5.00) for males, −0.60 (4.65) for females; *t*-test (936) =  −3.74, *P* < 0.001].

To evaluate the predictive validity of the DunedinARB, we tested its association with measures of midlife brain structural and functional integrity that have been shown, in other samples, to be predictive of neurodegenerative disease in older adults^16,31–33^ (described in Supplementary Table 2). Three MRI measures of brain structural integrity were tested: (i) the brain Age Gap Estimate (brainAGE),^34^ which is the difference between an individual’s chronological age at the time of imaging and their ‘brain age,’ as estimated by a machine-learning algorithm trained to predict chronological age from grey- and white-matter MRI measures in independent samples ranging in age from 19 to 82;^35^ (ii) log-transformed volume of white matter hyperintensities,^36^ a measure of ischaemic and general white matter pathology and (iii) mean grey matter volume of the hippocampus,^37^ a brain region central to both healthy memory function and age-related memory decline.^38^ In addition to the measures of brain structural integrity, three measures of brain functional integrity were tested: (i) objective cognitive function^39^ (the Wechsler Adult Intelligence Scale-IV, full-scale IQ); (ii) subjective problems in everyday cognitive function,^39^ such as misplacing eyeglasses, getting easily distracted, or forgetting errands, as reported on by Study members and up to three informants who knew them well and (iii) objective cognitive decline^39^ (measured as residualized change in full-scale IQ scores from childhood to age 45 years, assessed via matched Weschler Intelligence scales).

Across all six measures, the DunedinARB score proved informative about midlife brain health (Fig. 3B). First, Study members with higher DunedinARB scores demonstrated lower structural brain integrity: each standard deviation increase in the DunedinARB was associated with an additional 1.77-years older algorithm-estimated brainAGE (95% CI: 1.25, 2.29, *P*-value < 0.001, *β* = 0.22), a 0.16-log mm^3^ greater volume of white matter hyperintensities (95% CI: 0.10, 0.21, *P*-value < 0.001, *β* = 0.19), and a 79.04 mm^3^ smaller hippocampal grey matter volume (95% CI: −129.82, −28.27, *P*-value = 0.003, *β* = –0.09). *Post hoc* adjustment for total brain volume identified that DunedinARB associations with hippocampal volume were non-specific (adjusted-*β* = −0.01, *P*-value = 0.778) and likely represented an overall trend of smaller brain volumes among individuals with greater risk scores.

Second, study members with higher DunedinARB scores demonstrated lower cognitive function: each standard deviation increase in the DunedinARB was associated with an additional 7.39-point lower score in full-scale IQ (95% CI: −8.25, −6.54, *P*-value < 0.001, *β* = −0.49) and a 0.29 SD higher score on self and informant-reported scales assessing everyday cognitive problems (95% CI: 0.24, 0.34, *P*-value <0.001, *β* = 0.38).

Third, Study members with higher DunedinARB scores demonstrated greater longitudinal decline in cognitive function from childhood to adulthood, a hallmark antecedent of ADRD:^40^ each standard deviation increase in the DunedinARB was associated with an additional 2.51-point decline in full-scale IQ score from childhood to midlife (95% CI: −3.11, −1.90, *P*-value < 0.001, *β* = –0.26).

Supplementary Table 5 presents the association of the 10 domains of risk comprising the DunedinARB (Fig. 1) with the midlife outcome measures.

### Construction of published risk indexes and comparison to the benchmark DunedinARB

Scores for four published pre-morbid ADRD risk indexes that have received considerable research attention to date were generated for each Dunedin Study member who attended the age-45 assessment wave (*N* = 938). For all risk factors included in each index, study members were assigned risk points following the index’s published guidelines (Supplementary Table 3). The four indexes were:

1. the Cardiovascular Risk Factors, Aging, and Incidence of Dementia risk index (CAIDE, model-2; eight indicators, including genetic risk),^12,14,15^
2. the LIfestyle for BRAin health (LIBRA) index (12 indicators),^10,41,42^
3. the Lancet Commission on Dementia Prevention, Intervention, and Care risk factor list (Lancet; 12 indicators)^43^ and
4. the Australian National University Alzheimer's Disease Risk Index (15 indicators).^11,44^

#### What is the population distribution of risk as measured by published ADRD risk indexes?

**Figure 4:**
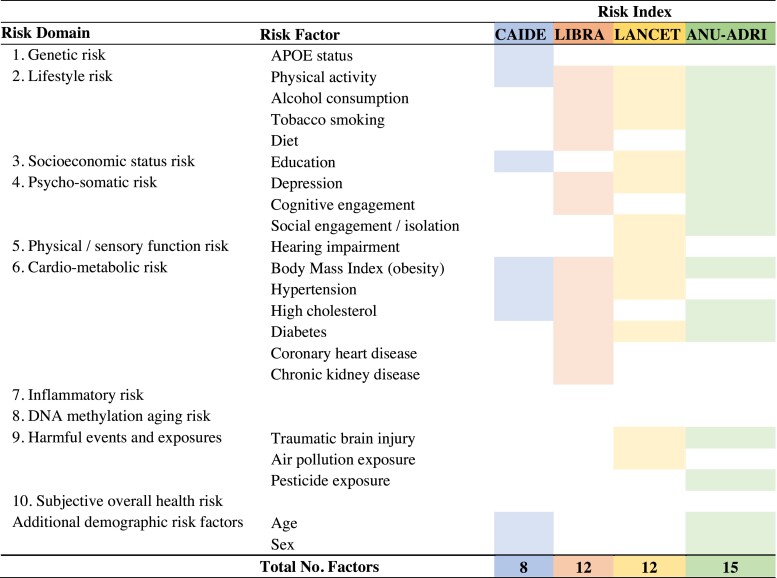
**Composition of the four published ADRD risk indexes.** The DunedinARB includes direct or proxy measures of all risks listed here with the exception of air pollution exposure and cognitive engagement. Age and sex did not contribute to the DunedinARB score as the cohort is of equal age and sex was retained for use in analyses as a covariate to account for known sex-differences in brain structure.

Fig. 4 presents the risk factors that comprise each of the four published ADRD risk indexes. The CAIDE includes 8 risk indicators, the LIBRA 12, the Lancet 12, and the ANU-ADRI 15. Altogether, the four indexes utilize a total of 20 unique indicators (when compared with the 48 included in the benchmark DunedinARB). Consequently, a large number of proposed risk factors with consistent empirical support of predicting dementia were not included in any index (e.g. poor sleep,^45,46^ poor sense of smell,^47–49^ disadvantageous personality traits,^50,51^ etc.).

**Figure 5:**
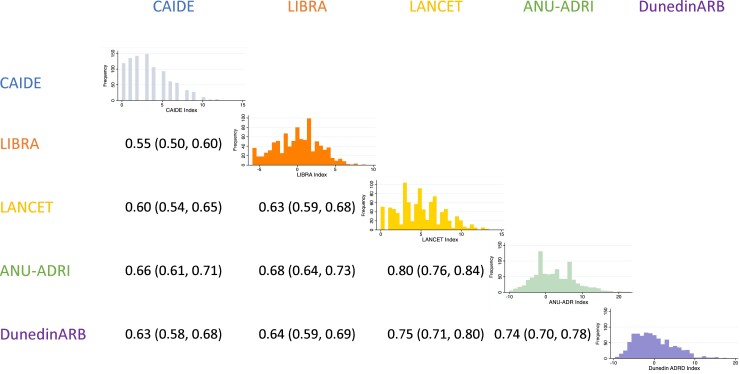
**Distribution and correlation (overlap) of ADRD risk among Dunedin Study members as measured by the four published risk indexes and the DunedinARB.** Cells below the diagonal present pairwise Pearson’s *r* correlation coefficients (95% confidence intervals). All correlations are statistically significant, *P* < 0.001. Cells on the diagonal present histograms showing the distribution of each risk index in the Dunedin Study cohort and the DunedinARB benchmark.

Despite their inclusion of different, conceptually distinct risk factors, there was moderate to high correlation among the risk indexes (Pearson’s *r*'s between 0.55 and 0.80, *P*-values < 0.001) (Fig. 5), indicating that they largely ranked Study members’ risk similarly, although rankings were not interchangeable. The distribution of risk scores was similar across the four indexes and DunedinARB (Fig. 5).

Table 1 presents the overlap (correlation) of the four published indexes with the 10 domains of risk (e.g. genetic, lifestyle, etc.) captured by the DunedinARB’s 48 indicators. Cells highlighted in yellow indicate risk domains specifically represented in the construction of each index, documenting that each risk index, by design, was missing some risk information. The figure reveals two points of interest. First, despite missing some risk information, each risk index captured information about risk domains that were not directly assessed in that index. For example, high LIBRA scores reflect socioeconomic risk as much as high CAIDE, Lancet, and ANU-ADRI scores do, although socioeconomic risk is not included in the LIBRA index but is included in the three other indexes. Second, some risk information was notably absent from some risk indexes. For example, genetic risk is not reflected in high LIBRA, Lancet, or ANU-ADRI scores; CAIDE and LIBRA only weakly capture risks associated with harmful events and exposures (e.g. TBI, neurotoxicant exposures, etc.); and CAIDE does not capture psychological and somatic risk.

**Table 1:** Associations of the 10 domains of risk comprising the DunedinARB with the four published risk indexes

	CAIDE (8 risk factors)	LIBRA (12 risk factors)	LANCET (12 risk factors)	ANU-ADRI (15 risk factors)
	**Pearson’s *r***			
1. Genetic risk	**0**.35***	−0.02	0.06	0.02
2. Lifestyle risk	0.40***	**0**.58***	**0**.58***	**0**.62***
3. Socioeconomic status risk	**0**.56***	0.51***	**0**.45***	**0**.56***
4. Psychological and somatic risk	0.08*	**0**.29***	**0**.45***	**0**.40***
5. Physical and sensory function risk	0.28***	0.30***	**0**.50***	0.35***
6. Cardio-metabolic risk	**0**.50***	**0**.42***	**0**.37***	**0**.36***
7. Inflammatory risk	0.18***	0.25***	0.25***	0.27***
8. Epigenetic aging risk	0.23***	0.20***	0.25***	0.24***
9. Harmful events and exposures	0.11***	0.12***	**0**.24***	**0**.26***
10. Subjective overall health	0.38***	0.45***	0.51***	0.51***

Cells highlighted with bold text indicate risk domains specifically represented in the construction of the risk index. *P-values < 0.05, **P-values < 0.01, ***P-values < 0.001.

#### Are published risk indexes informative about midlife brain health?

Table 2 presents the association of the four published risk indexes with the midlife measures of brain structural and functional integrity that are antecedents of ADRD. Despite being comprised of risk factors for dementia in old age, all four risk indexes were significantly associated with most measures of midlife brain health, ∼20–40 years before dementia diagnoses are expected.

**Table 2:** Comparison of the four published risk indexes against the DunedinARB in associations with midlife measures of brain structural and functional integrity, adjusted for sex

	Brain structural integrity (MRI) measures			Brain functional integrity (cognitive) measures		
	brainAGE	Log WMH volume	Hippocampal volume	Objective midlife IQ	Subjective midlife cognitive problems	IQ change from childhood
		*β*'s (95% CI)			*β*'s (95% CI)	
CAIDE	0.16 (0.08, 0.23)	0.16 (0.09, 0.23)	−0.09 (−0.15, −0.03)	−0.44 (−0.50, −0.37)	0.14 (0.08, 0.21)	−0.18 (−0.25, −0.11)
LIBRA	0.17 (0.10, 0.24)	0.13 (0.06, 0.19)	−0.08 (−0.14, −0.02)	−0.36 (−0.42, −0.30)	0.28 (0.22, 0.34)	−0.16 (−0.22 −0.09)
LANCET	0.20 (0.14, 0.27)	0.14 (0.07 0.20)	−0.11 (−0.17, −0.05)	−0.39 (−0.45, −0.33)	0.32 (0.26, 0.38)	−0.15 (0.21, −0.09)
ANU-ADRI	0.22 (0.15, 0.28)	0.13 (0.06, 0.20)	−0.08 (−0.14, −0.02)	−0.43 (−0.49, −0.37)	0.30 (0.24, 0.36)	−0.20 (−0.27, −0.14)
DunedinARB	0.22 (0.16, 0.29)	0.19 (0.13, 0.26)	−0.09 (−0.15, −0.03)	−0.49 (−0.55, −0.44)	0.38 (0.32, 0.44)	−0.26 (−0.33, −0.20)

brainAGE, brain Age Gap Estimate, is the difference between an individual’s chronological age at the time of imaging and their ‘brain age,’ as estimated by a machine-learning algorithm trained to predict chronological age from MRI measures in independent samples ranging in age from 19 to 82. WMH, white matter hyperintensities.

First, study members with higher ADRD risk scores had lower structural brain integrity, including older algorithm-estimated brainAGE (*β*'s between 0.16 and 0.22, *P*-values < 0.001), greater volume of white matter hyperintensities (*β*'s between 0.13 and 0.16, *P*-values < 0.001), and smaller hippocampal grey matter volume (*β*'s between –0.08 and –0.11, *P*-values < 0.05). *Post hoc* adjustment for total brain volume identified that risk index associations with hippocampal volume were non-specific (adjusted *β*'s between −0.02 and −0.05, *P*-values > 0.05) and likely represented an overall trend of smaller brain volumes among individuals with greater risk scores.

Second, Study members with higher ADRD risk scores demonstrated lower midlife cognitive function, including lower full-scale IQ scores (*β*'s between −0.36 and −0.44, *P*-values < 0.001) and higher scores on scales assessing everyday cognitive problems (*β*'s between 0.14 and 0.32, *P*-values < 0.001).

Third, Study members with higher ADRD risk scores demonstrated greater longitudinal decline in cognitive function from childhood to adulthood, a hallmark premorbid feature of ADRD (*β*'s between −0.15 and −0.20, *P*-values< 0.001).

#### Do published risk indexes represent an efficient mix of putative risk factors for ADRD?

In terms of association with the midlife measures of brain structural and functional integrity that are antecedents of ADRD, all four published risk indexes compared favourably to the benchmark DunedinARB (Table 2), and no one risk index notably outperformed all others across all outcomes. Published risk indexes thus likely represent an efficient mix of risk factors for ADRD, at least at midlife, despite only including a small portion of putative risks. The inclusion of more risk factors, as in the DunedinARB, tended to broadly increase associations with the outcome measures, but only modestly, and not always significantly.

On the MRI measures of brain structural integrity, the DunedinARB tended to outperform the four published indexes, but the improvements were small (difference in *β*'s < 0.06). Tests of differences between dependent correlations^52^ revealed that the DunedinARB significantly outperformed the CAIDE in its association with brainAGE (DunedinARB sex-adjusted *β* = 0.22, 95% CI: 0.16, 0.29), and outperformed the LIBRA, Lancet, and ANU-ADRI in its association with white matter hyperintensity volume (DunedinARB sex-adjusted *β* = 0.19, 95% CI: 0.13, 0.26). There were no significant differences among published index or benchmark associations with hippocampal volume.

On the cognitive measures, the DunedinARB always outperformed the four published indexes, as indicated by tests of dependent correlations,^52^ but improvements varied by cognitive outcome. Differences in *β*'s between the DunedinARB and the published indexes ranged from 0.05 to 0.13 for objective midlife IQ and 0.06 to 0.24 for subjective cognitive problems.

On the measure of longitudinal cognitive decline, the DunedinARB again consistently outperformed all of the published indexes (sex-adjusted *β* = −0.26, 95% CI: −0.32, −0.20), as indicated by tests of dependent correlations (differences in *β*'s ranged from 0.06 to 0.11).

These findings indicated that information contained in the DunedinARB and missing from the published indexes was uniquely informative about cognitive ability and, notably, longitudinal cognitive decline unfolding across adulthood. To evaluate this hypothesis further, we performed a number of *post hoc* sensitivity tests. First, models regressing the residualized cognitive decline measure on the DunedinARB were re-estimated including each of the four published risk indexes as covariates, in turn, to determine whether the DunedinARB was a significant predictor of cognitive decline over and above each published risk index (four new tests in total), and it was. The DunedinARB remained significantly associated with cognitive decline in all models including the four risk indexes. Second, to determine whether a particular risk domain within the DunedinARB was a significant predictor of cognitive decline over and above each published risk index, models regressing the residualized cognitive decline measure on the 10 risk domains comprising the DunedinARB were estimated including each of the 4 published risk indexes in turn (40 new tests in total). Results of these tests are reported in Supplementary Table 6. Four domains of risk were significantly associated with cognitive decline over and above all published indexes (socioeconomic status risk, physical and sensory function risk, epigenetic aging risk and subjective overall health risk).

## Discussion

We compared 4 published ADRD risk indexes to a comprehensive benchmark comprising 48 indicators covering 10 domains of risk and mapped all 5 ADRD-risk scores onto midlife measures of brain structural and functional integrity that are antecedents of ADRD. This evaluation generated seven findings.

First, despite consistent overlap among distinct domains of ADRD risk (e.g. individuals with greater lifestyle-based risks such as tobacco smoking also had greater epigenetic risks, such as advanced cellular aging), all 10 domains of ADRD risk were found to be non-redundant; each added some unique information to a comprehensive benchmark. Second, midlife risk for later-life ADRD followed an approximately normal distribution on all ADRD-risk scores in our population-representative cohort. While ADRD diagnosis is categorical, relative risk appears to be quantitatively distributed, with the majority of the cohort, in midlife, at low-to-moderate risk of late-life ADRD. This quantitative risk distribution poses a challenge to decision-making about premorbid dementia surveillance and therapeutics, given that cut-offs are not yet known.

Third, dementia risk indexes were informative about brain structural and functional integrity decades before the expected emergence of clinical diagnosis. Study members with higher risk scores demonstrated older brains (i.e. older algorithm-estimated brain age from MRI data), more white matter pathology, smaller hippocampi (a brain region critical for memory function and a primary locus of neurodegeneration in Alzheimer’s dementia) likely reflecting smaller overall brains, relative deficits in tested cognitive performance, elevated subjective problems in everyday cognitive function, and longitudinal cognitive decline across adulthood, all at the relatively young age of 45. These findings reinforce the emerging perspective, voiced by the Lancet Commission on Dementia Prevention, Intervention, and Care, that it is ‘never too early’ to prevent dementia.^43(p1)^

Fourth, published risk indexes comprised of small subsets of putative ADRD risk factors were correlated; they tended to rank the same individuals as at higher or lower-risk for ADRD as each other, despite each including a different collection of risk factors. All published indexes (the CAIDE, the LIBRA, the Lancet, and the ANU-ADRI) were found to contain information about domains of risk that they did not directly assess, including risk related to systemic inflammation, epigenetic aging, and subjective overall health. Individual indexes were, however, found to selectively lack information about particular domains of risk (Table 1), notably genetic risk, harmful events and exposures (e.g. history of TBI), and psycho-somatic risks (e.g. history of depression), depending on the particular index.

Fifth, published risk indexes were determined to represent an efficient mix of ADRD risk factors, as they tended to perform nearly as well as the more comprehensive benchmark DunedinARB in their association with the outcome-measure antecedents of ADRD, particularly those of brain structural integrity. The DunedinARB outperformed the published indexes in its associations with these structural measures, but only modestly, and not always significantly. For example, the shortest index, the CAIDE, demonstrated a similar (although weaker) pattern of findings to the comprehensive DunedinARB, despite being comprised of 80% fewer indicators. This suggests that some conceptually distinct risk factors supported by the current literature are best considered proxy measures of other risk factors (and not unique contributors to risk). Other risk factors may only influence brain health after midlife. This latter potential would reinforce the Lancet Commission’s life-course model of dementia prevention,^43^ which argues that the influence of ADRD risk factors can vary across the life-course.

Sixth, while published ADRD risk indexes were found to perform well against the DunedinARB on many outcome measures, *post hoc* sensitivity tests determined that the DunedinARB was informative over and above each of the published indexes about cognitive decline unfolding across adulthood. This additional predictive value could be accounted for, in part, by the DunedinARB’s inclusion of risk domains that have largely been overlooked by existing indexes, including socioeconomic risk, physical and sensory function risk, epigenetic risk, and subjective overall health risk (Supplementary Table 6). This suggests that rounding out the four published indexes by adding any of these selectively missing risk domains may improve their ability to select individuals at-risk for ADRD. This could be done in a manner that maintains the unique benefits of each particular index (e.g. using self-report measures for the ANU-ADRI, making use of information readily available to physicians for the CAIDE, etc.).

Seventh, a close look at subjective cognitive complaints, here measured as reports by Study members and informants about everyday cognitive problems, such as misplacing eyeglasses, getting easily distracted, or forgetting errands, is informative because subjective complaints typically bring patients to clinical attention, lead them to volunteer for intervention trials, or prompt them to request medications. Ninety-three percent of this midlife cohort had one or more subjective complaints, and 75% had three or more. Comparison of the published indexes with the comprehensive DunedinARB suggested that existing indexes could do a better job of capturing risks related to developing cognitive complaints. Research in larger samples can inform as to which index best discriminates conversion to ADRD among the pool of individuals presenting to clinicians with subjective cognitive complaints.

## Limitations

This study has several limitations. First, it was observational and cannot establish causation. Reverse causation is possible; for example, cognitive decline may have promoted socioeconomic-status risk. However, for purposes of risk prediction, causation is not a central consideration. Second, we investigated only one cohort in one country and findings should be replicated elsewhere. Third, brain-health measures of ADRD antecedents were largely, although not exclusively, cross-sectional midlife measures. Continued follow-up will be required to more precisely estimate ADRD risk (for example, using plasma biomarkers) as well as to document risk factor associations with further longitudinal declines in brain integrity. Fourth, while the DunedinARB included most known or suspected measures of ADRD risk, some measures were unavailable, including blood, cerebrospinal fluid (CSF), or positron emission tomography (PET) ADRD biomarkers or exposure to outdoor air pollution, which could be added for further investigation.

## Conclusion

With the coming advent of successful yet costly premorbid ADRD interventions on the horizon, the time has arrived to refine screening systems to differentiate the truly high-risk in urgent need of preventive treatment from the moderate-risk majority and legions of worried-well, for whom benefits may not outweigh side-effects. With a dizzying array of factors that may elevate dementia risk differentially across the lifespan, it can be difficult to know where to focus clinical and research attention. Our study findings suggest that risk indexes are informative about risk as early as midlife, with differences in brain integrity already apparent by age 45. Findings also suggest that published ADRD risk inventories, including the CAIDE, the LIBRA, the Lancet, and the ANU-ADRI, perform well in identifying linear gradients of risk among members of the general population, even when they include only a small subset of potential risk factors. Our findings also suggest, however, that these published risk indexes could be improved with targeted additions to more holistically capture the different facets of risk that contribute to these costly and difficult-to-treat diseases of late-life.

## Supplementary Material

fcac223_Supplementary_Data

## Abbreviations

ADRD =: Alzheimer’s disease and related dementias
ANU-ADRI =: Australian National University Alzheimer’s Disease Risk Index
brainAGE =: brain Age Gap Estimate
CAIDE =: Cardiovascular Risk Factors, Aging, and Dementia index
DunedinARB =: Dunedin Alzheimer’s disease and related dementias Risk Benchmark
LIBRA =: LIfestyle for BRAin Health index
TBI =: traumatic brain injury.
