# Supplementary material for: Improving risk indexes for Alzheimer’s disease and related dementias for use in midlife
Source: Brain Commun. 2022 Oct 6;4(5):fcac223. doi: 10.1093/braincomms/fcac223 (PMC9535507; doi:10.1093/braincomms/fcac223)
Supplement: fcac223_Supplementary_Data [file fcac223_supplementary_data.zip › Supplementary_material.docx]

**Supplement**

For manuscript entitled, “Improving risk indexes for Alzheimer disease and related dementias for use in midlife.”

List of materials

[Supplementary Appendix 1. Age-45 Data Collection Phase attrition analysis 2](#_Toc104973684)

Supplementary [Appendix 2. Methodology for the identification of ADRD risk factors for the DunedinARB. 5](#_Toc104973685)

Supplementary [Table 1. Description of the risk indicators included in the Dunedin ADRD Risk Benchmark and assignment of risk points. 6](#_Toc104973686)

Supplementary [Table 2. Description of the midlife brain integrity measures. 21](#_Toc104973687)

Supplementary [Table 3. Assignment of risk points and weighting for each risk factor in the four published ADRD indices. 23](#_Toc104973688)

Supplementary [Table 4. Correlations among individual risk indicators within the 10 classes of risk comprising the DunedinARB. 30](#_Toc104973689)

Supplementary [Table 5. Association of the 10 domains of risk comprising the DunedinARB with the midlife measures of brain integrity. 35](#_Toc104973690)

Supplementary [Table 6. Association of the 10 domains of risk comprising the DunedinARB with longitudinal cognitive decline over and above the 4 published risk indices. 36](#_Toc104973691)

Supplementary [References 37](#_Toc104973692)

# Supplementary Appendix 1. Age-45 Data Collection Phase attrition analysis

We conducted an attrition analysis using childhood IQ, childhood SES, p-factor (using ages 18-45 year data), Adverse Childhood Events (ACEs), and a polygenic score for educational attainment to determine whether participants in the Phase 45 data collection were representative of the original cohort.

No significant differences in childhood IQ were found between the full cohort, those still alive, those seen at Phase 45 or those who underwent brain scans at Phase 45. Those who were deceased by the Phase 45 data collection had significantly lower childhood IQ’s than those who were still alive (t = 2.09, p = 0.04).


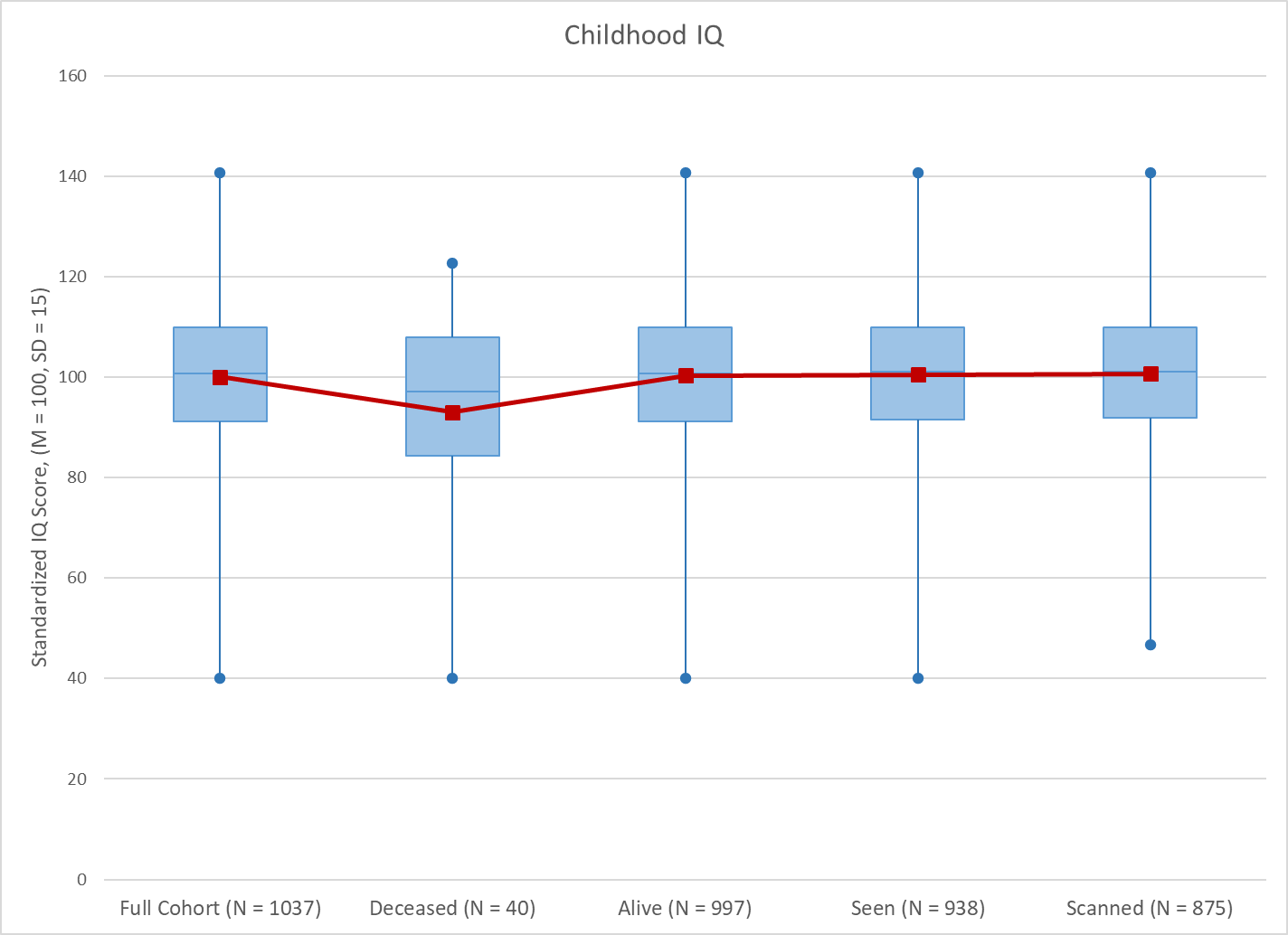


No significant differences were found between the full cohort, those deceased, those alive, those seen at Phase 45 or those scanned at Phase 45 on childhood SES.


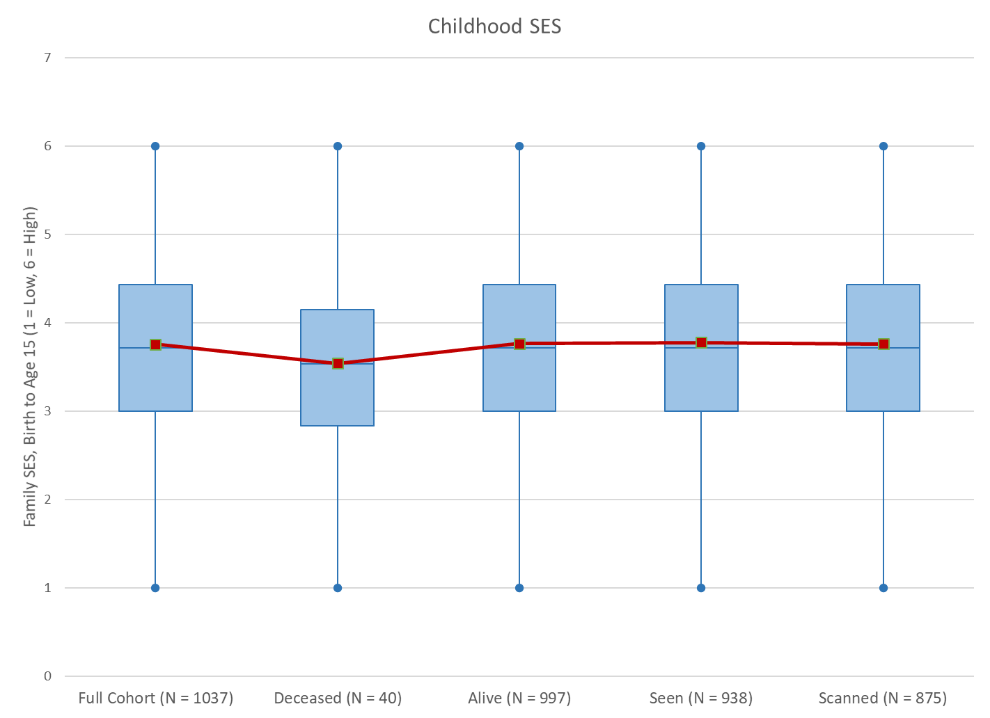


No significant differences in p-factor were found between the full cohort, those still alive, those seen at Phase 45 or those scanned at Phase 45. Those who were deceased by the Phase 45 data collection had significantly higher p-factor scores than those who were still alive (t = -2.86, p = 0.004).


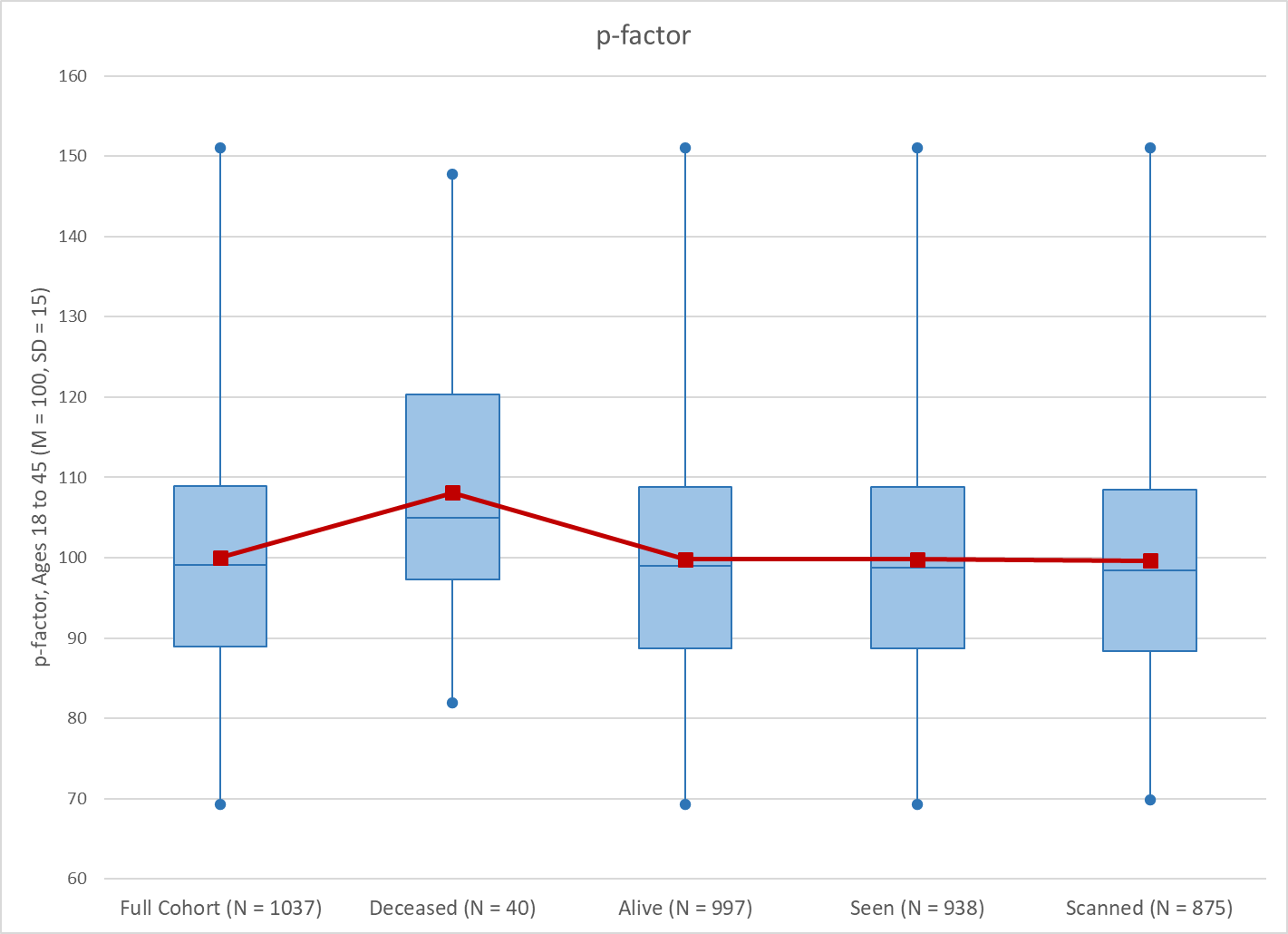


No significant differences were found between the full cohort, those deceased, those alive, those seen at Phase 45 or those scanned at Phase 45 on Adverse Childhood Events (ACEs).


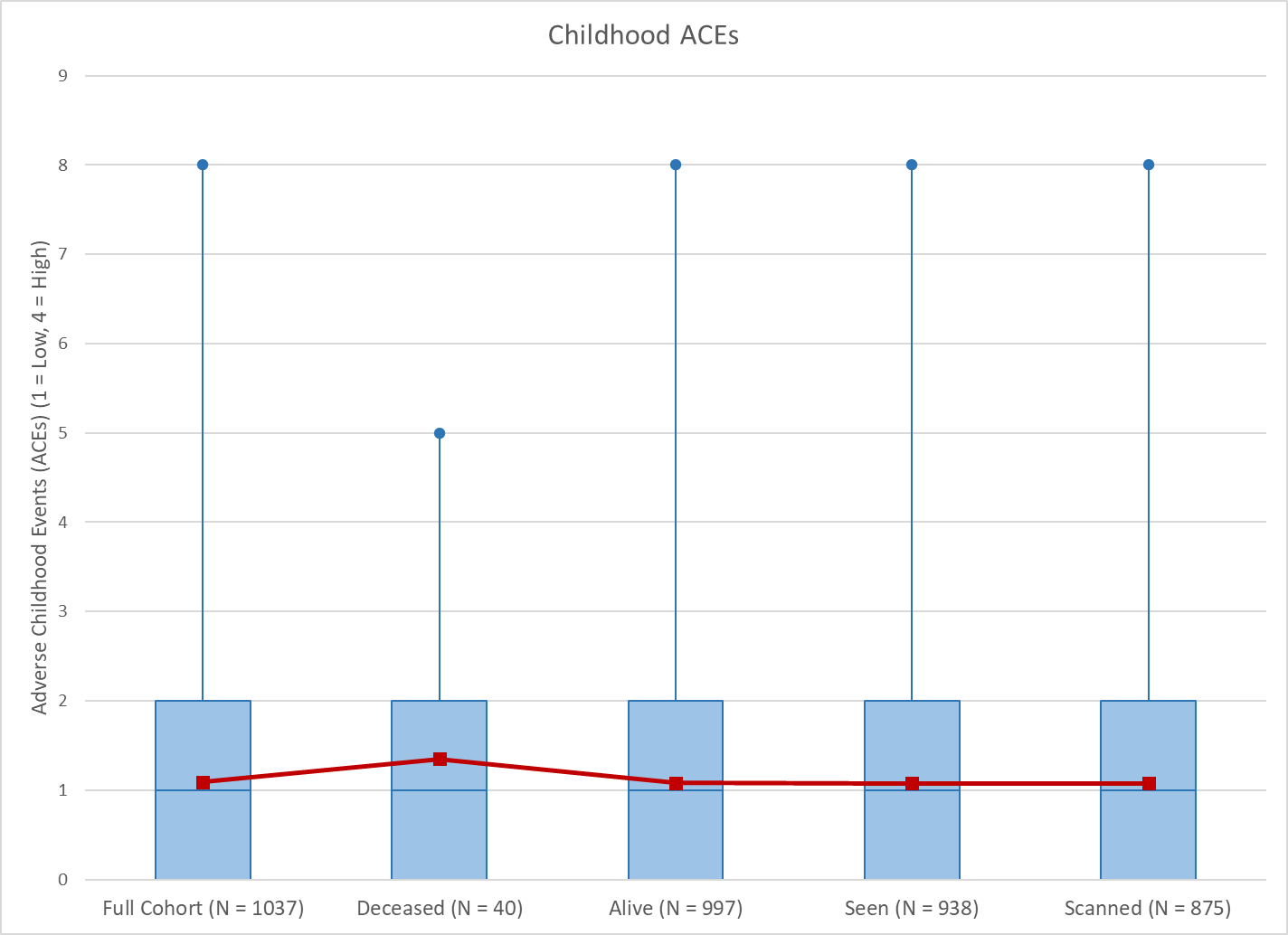


No significant differences were found between the full cohort, those deceased, those alive, those seen at Phase 45 or those scanned at Phase 45 on Childhood Low Self-Control.


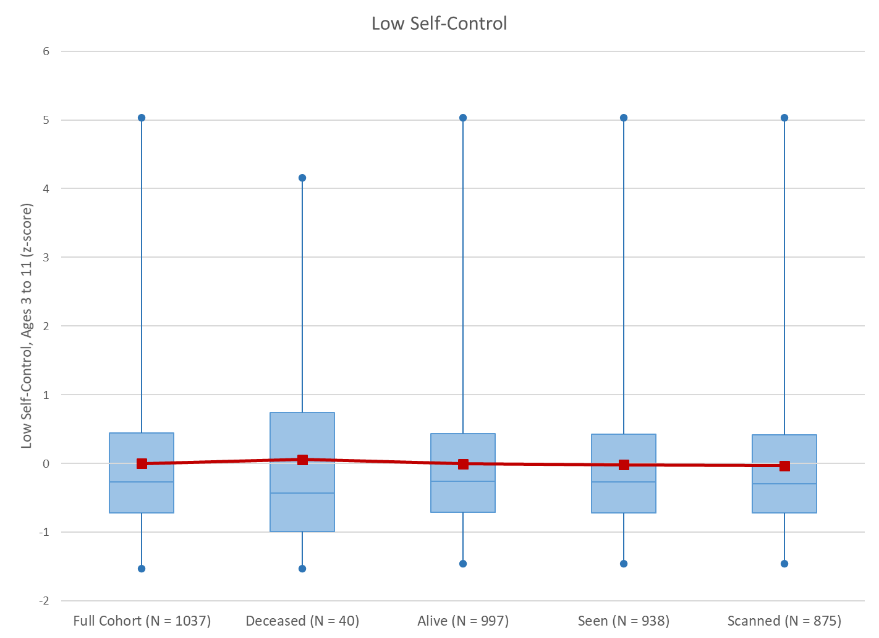


We began collecting DNA from Study members at age 26, in 1998. The DNA biobank does not contain DNA from Study members of Maori descent. No significant differences were found between non-Maori participants with DNA, those who subsequently died, those alive, those seen at Phase 45 or those scanned at Phase 45 on the SSGAC 2021 polygenic score for educational attainment.


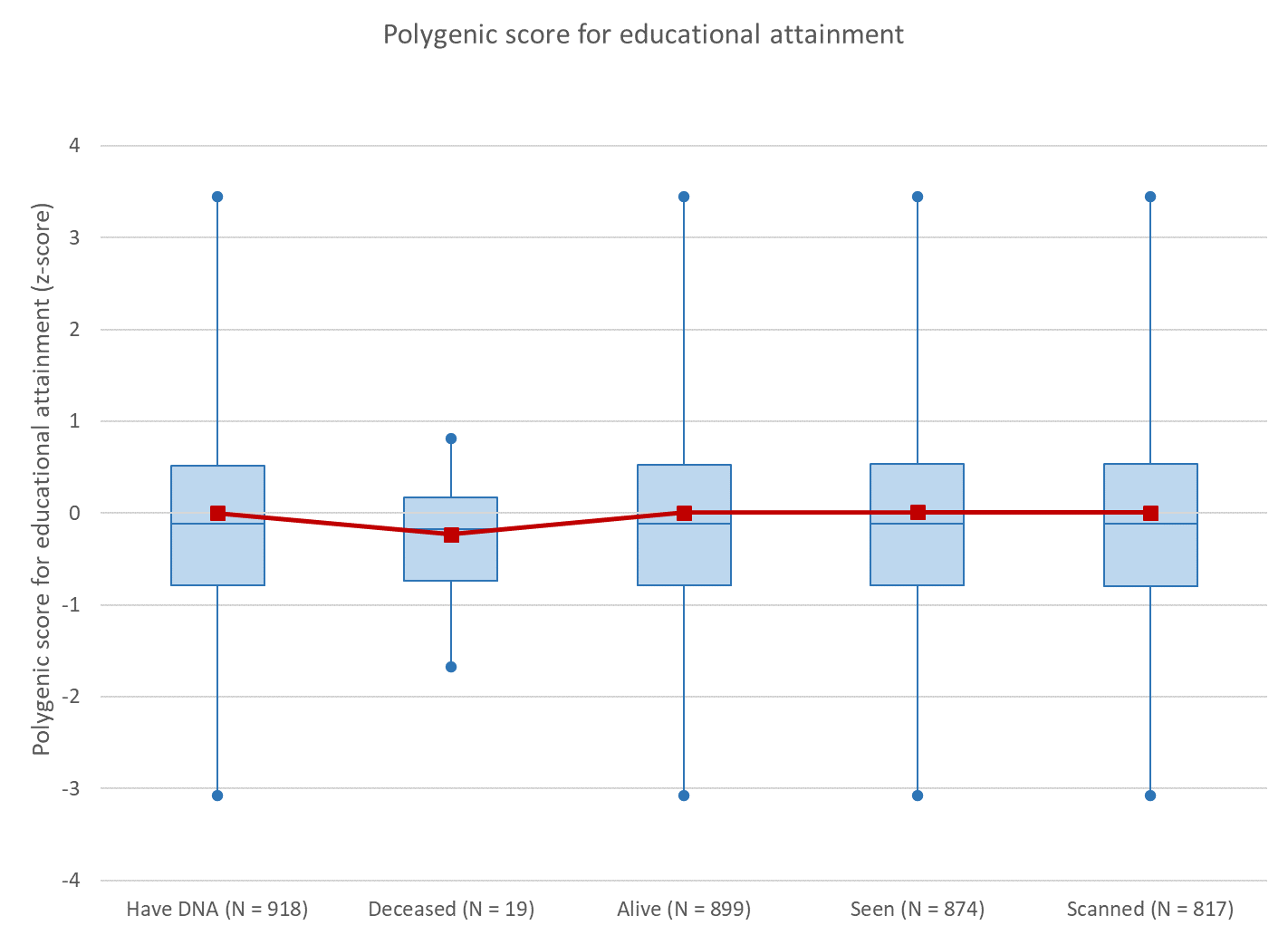


# Supplementary Appendix 2. Methodology for the identification of ADRD risk factors for the DunedinARB.

Established and proposed risk factors for ADRD were selected for inclusion in the Dunedin ADRD Risk Benchmark (DunedinARB) following two steps. First, risk factors were selected based on their identification as risk factors through comprehensive review by: 1) the 2011 NIH State of the Science Consensus Panel on ADRD risk factors,^1^ 2) the Lancet Commission on Dementia Prevention, Intervention, and Care,^2^ and 3) the published ADRD risk indexes included in the comparison stage of the present study (the CAIDE,^3–5^ the LIBRA,^6–8^ and the ANU-ADRI^9,10^). Second, additional risk factors were identified through two separate non-systematic searches of PubMed, Scopus, and Google Scholar by the senior author (TEM) and first author (AR) for these five key words: Alzheimer’s, dementia, risk, review, meta-analysis. These searches were intended to cast the widest net possible and risk factors were selected for inclusion in the DunedinARB if at least two quality studies reported statistically significant evidence of an association with dementia diagnoses in two separate samples, ideally from two separate populations. For the vast majority of risk indicators selected for the DunedinARB through the second search step, the authors found more than two studies reporting a significant risk-factor-dementia association. For example, the personality traits of conscientiousness and neuroticism, which have yet to be included in any ADRD risk index or consensus summary, have been associated with dementia risk in 8-12 different samples, with “robust evidence” identified in two separate systematic review and meta-analyses.^11,12^

# Supplementary Table 1. Description of the risk indicators included in the Dunedin ADRD Risk Benchmark and assignment of risk points.

| Risk domain^a^ | Risk indicator | Description | Risk point assignment^b^ |
| --- | --- | --- | --- |
| Genetic risk^13,14^ | Family history of dementia | Study members reported at age 45 on family history of dementia. (N=925 with present data before imputation, 99% of the analytic sample). | Risk points were assigned (0 [92.8% of cohort]; 1 [7.1%]; or 2 [0.1%]) based on number of parents with diagnosed dementia. These match the prevalence rates of dementia in the wider New Zealand population^15^ and other similar populations characterized by a large proportion of individuals of European descent (e.g., the United States).^16^ |
|  | APOE ε4 allele status | Number of APOE ε4 alleles was assessed. APOE protein iosoforms E2/E3/E4 were derived from phased haplotypes of SNPs rs7412 and rs429358 assayed on a genome-wide array, Infinium OmniExpress-12 v1.1 BeadChip array (Illumina Inc., San Diego, California). (N=848 with present data before imputation, 90% of the analytic sample). | Risk points were assigned (0 [69.7%]; 1 [27.2%]; or 2 [3.1%]) based on the number of ε4 alleles present. The overall APOE E4 allele frequency was 17.0%, which is typical for people of European descent, somewhat smaller than what is typical for people of African descent, and somewhat larger than what is typical for people of Asian descent.^17^ |
|  |  |  |  |
| Lifestyle risk^18–23^ | Physical activity | Study members reported at age 45 on hours per week of leisure-time physical activity at moderate or greater levels of intensity. Cohort range: 0 to 22, mean(SD) = 2.82 (3.26). (N=908 with present data before imputation, 97% of the analytic sample). | Risk points were assigned based on World Health Organization guidelines for adults aged 18-64 years^24^: -1 = >3hrs weekly (33.8%); 0 = 1-3hrs weekly (31.0%); 1 = <1hr weekly (35.2%). |
|  | Diet | Study members reported at age 45 on their weekly diet. A scale assessing adherence to a Mediterranean-style diet was comprised of Study member report about their typical consumption per week (none, <1, 1-2 times, 3-4 times, most days, or daily) of meat, fish, nuts, beans, fruit, vegetables, sugary drinks, and extra virgin olive oil. Higher scores indicate closer adherence to a healthy diet (higher in fish, nuts, beans, fruit, vegetables, and olive oil, and lower in meat and sugary drinks). Resulting scale was z-scored, cohort range = -2.84 to 2.69; mean(SD) = 0(1). (N=900 with present data before imputation, 96% of the analytic sample). | Risk points were assigned as: -1 = >1SD above the mean (15.8%); 0 = within 1 SD of the mean inclusive (70.9%); 1 = < 1SD below the mean (13.3%). |
|  | Tobacco smoking | Study members reported at age 45 on daily tobacco smoking habits. (N=924 with present data before imputation, 99% of the analytic sample). | Risk points were assigned based on classification as non-smokers (0 [47.8% ]), former smokers (1 [30.3%]), and current smokers (2 [22.0%]). |
|  | Alcohol consumption | Study members reported at age 45 on weekly alcohol consumption habits. Consistent with published ADRD risk indices^7,9^, moderate drinkers were designated as lower risk that non-drinkers or heavy drinkers. (N=938 with present data before imputation, 100% of the analytic sample). | Risk points were assigned based on classification as light drinkers (-1 [40.5%]), non-drinkers (0 [7.3%]), and heavy drinkers (1 [52.2%]). Light drinkers consumed ≤ 7 or 14 drinks per week, respectively for females and males, and reported fewer than 6 occasions of binge drinking (5 or more drinks in a single occasion) in the past year. |
|  | Folic acid supplementation | Study members reported at age 45 on whether they regularly took folic acid or B complex supplementation. (N=938 with present data before imputation, 100% of the analytic sample). | Risk points were assigned as: -1 = Yes (1.8%); 0 = No (98.2%). |
|  | Regular prophylactic NSAID use | Study members reported at age 45 on whether they regularly took non-steroidal anti-inflammatory drugs (NSAIDs). (N=938 with present data before imputation, 100% of the analytic sample). | Risk points were assigned as -1 = Yes (20.8%); 0 = No (79.2% of cohort). |
|  |  |  |  |
| Socioeconomic risk^25–27^ | Occupational attainment | Study members reported at age 45 on their occupational attainment. Occupations were scored using the New Zealand Socioeconomic Index (NZSEI-06), which codes each occupation based on its associated education level and income in the NZ Census (score range, 10 [low status]-90 [high status]) and groups occupations into 6 status levels. Examples of occupations in the 6 groups include medical practitioner (NZSEI code 90; group 6), engineering professional (code 66; group 5), database administrator (code 59; group 4), personal assistant (code 44; group 3), office cashier (code 28; group 2), and fish filleter (code 23; group 1). Homemakers and others not working in the past year were assigned the status of their most recent occupation, as reported at age 38. Study members who had been out of the labor force since age 32 were assigned the status of their partner if they had a partner with whom they shared a household. (N=935 with present data before imputation, 99% of the analytic sample). | Risk points were assigned as: -1 = High status (groups 5 and 6) (33.2%); 0 = Middle status (groups 3 and 4) (46.2%); 1 = Low status (groups 1 and 2) (20.7%). |
|  | Educational attainment | Study member’s reported on their educational attainment by age 45^28^ and were grouped into ranked education levels of: 0 = no certifications (N = 138, 14.7%); 1 = school certification achieved (N = 135, 14.4%); 2 = high school graduate or equivalent (N = 376, 40.1%); and 3 = Bachelor’s degree or higher (N = 288, 30.7%). (N=937 with present data before imputation, 99% of the analytic sample). | Risk points were assigned as: -1 = Bachelor’s degree or higher (30.7%); 0 = high school graduate or equivalent / school certification (54.6%); 1 = no school certification (14.7%) |
|  |  |  |  |
| Psychological and somatic function risk^11,28–32^ | Pain interference with daily function | Study members reported at age 45 on pain interference with daily life (0=not at all, to 5=very much) via questions about the extent to which pain hinders engagement with social, cognitive, emotional, physical, and recreational activities. Cohort range: 0-20, mean(SD)=3.24(4.24). (N=908 with present data before imputation, 97% of the analytic sample). | Risk points were assigned as: 0 = ≤1 SD above the mean (84.8%); 1 = >1 SD above the mean (15.2%). |
|  | History of migraine | Study members reported at each assessment wave from age 26 to 45 about whether they had experienced frequent headaches lasting from 30 min to 7 days in the past year. Headache pain characteristics and symptoms were also assessed and headaches classified as either tensio-type or migraine.^33,34^ A count of phases with migraine headaches was created. (N=938 with present data before imputation, 100% of the analytic sample). | Risk points were assigned as: 0 = never met criteria for migraine in adulthood (75.4%); 1 = met criteria for migraine at at least one wave (24.6%). 47.2% of Study members who met criteria for migraine did so at multiple waves. |
|  | History of depression | Study members reported at each assessment wave from age 15 to 45 about about symptoms of Major Depressive Disorder over the past year. 50.5% of Study members met criteria for depression at at least one wave and 28.4% met criteria at multiple waves. (N=938 with present data before imputation, 100% of the analytic sample). | Risk points were assigned as: 0 = 1 or fewer episodes of depression across adulthood (71.6%); 1 = >1 episode of depression across adulthood (28.4%). |
|  | Loneliness / social isolation | Study members reported at age 45 on loneliness and social isolation via response (0=hardly ever, 1=some of the time, 2=often) to four items adapted from the UCLA Loneliness Scale^35^ (e.g., “How often do you feel you lack companionship?”; “How often do you feel isolated from others?”). Items were summed to create a loneliness / social isolation scale. Cohort range: 0-8, mean(SD)=1.21(1.77). (N=922 with present data before imputation, 98% of the analytic sample). | Risk points were assigned as: 0 = ≤1 SD above the mean (80.4%); 1 = >1 SD above the mean (19.6%). |
|  | Sleep quality | Study members reported at age 45 on sleep quality using the Pittsburgh Sleep Quality Index (PSQI).^36^ The PSQI consists of 18 self-report items relating to individuals’ sleep patterns and different forms of sleep impairment in the past month. These questions are used to derive scores for seven different components of sleep (subjective sleep quality, sleep latency, sleep duration, habitual sleep efficiency, sleep disturbances, use of sleep medication and daytime dysfunction), each scored from 0 to 3. These were summed to produce a global score ranging from 0 to 21, with higher scores reflecting worse sleep quality. Cohort range: 1-17, mean(SD)=6.24(2.41). (N=909 with present data before imputation, 97% of the analytic sample). | Risk points were assigned as: 0 = ≤1 SD above the mean (85.4%); 1 = >1 SD above the mean (14.6%). |
|  | Neuroticism & Conscientiousness | At the age 45 assessment, informants nominated by the Study members as people "who knew them well" were mailed questionnaires and asked to describe each Study member using a 25-item version of the Big Five Inventory, which measured the personality traits of Neuroticism and Conscientiousness.^37^ Items such as "Can be moody" and "Is emotionally stable, not easily upset" asssesed Neuroticism and items such as "Does a thorough job" and "Makes plans and follows through with them" assessed Conscientiousness. Resulting scales ranged from 0 to 10: cohort mean(SD) Neuroticism = 3.96(2.02) and Conscientiousness = 7.48(1.54). (N=883 with present data before imputation, 94% of the analytic sample). | Risk points for Neuroticism were assigned as: 0 = ≤1 SD above the mean (81.2%); 1 = >1 SD above the mean (18.8%). Risk points for Conscientiousness were assigned as: -1 = >1 SD above the mean (14.0%); 0 = ≤1 SD above the mean (86.0%). |
|  |  |  |  |
| Physical and Sensory function risk^38–43^ | Balance | Balance was measured at age 45 using the Unipedal Stance Test as the maximum time achieved across three trials of the test with eyes closed.^44^ Cohort range: 1-30, mean(SD)=14.58(9.72). (N=911 with present data before imputation, 97% of the analytic sample). | Risk points were assigned as: 0 = ≥1 SD below the mean (86.4%); 1 = <1 SD below the mean (13.6%). |
|  | Gait | Gait speed (meters per second) was assessed at age 45 with the 6-m-long GAITRite Electronic Walkway (CIR Systems, Inc) with 2-m acceleration and 2-m deceleration before and after the walkway, respectively. Gait speed was assessed under 3 walking conditions: usual gait speed (walk at normal pace from a standing start, measured as a mean of 2 walks) and 2 challenge paradigms, dual task gait speed (walk at normal pace while reciting alternate letters of the alphabet out loud, starting with the letter “A,” measured as a mean of 2 walks) and maximum gait speed (walk as fast as safely possible, measured as a mean of 3 walks). We calculated the mean of the 3 individual walk conditions to generate our primary measure of composite gait speed.^45^ Cohort range: 0.74-2.12, mean(SD)=1.41(0.19). (N=904 with present data before imputation, 96% of the analytic sample). | Risk points were assigned as: 0 = ≥1 SD below the mean (84.9 = <1 SD below the mean (15.1%). |
|  | Objective hearing function (hearing acuity) | Hearing thresholds were measured at age 45 by conducting pure-tone audiometry. In a sound-attenuating booth, pure-tones delivered in the following order of frequencies – 1000 Hz, 2000 Hz, 4000 Hz, 8000 Hz, 12500 Hz, and 500 Hz – were presented to the study members through headphones. Presentation intensity levels began at 40 decibels at hearing level (dB HL) for normal hearing study members, and 60 dB HL for hearing aid users. Study members used a response button to indicate whenever they heard a tone, and the lowest intensity level that elicited a response was identified as the hearing threshold for that frequency. A high PTA was calculated by averaging 8000 Hz and 12500 Hz. Results from the best ear are reported. Cohort range: -7.5 to 85, mean(SD)=22.12(14.75). | Risk points were assigned as: 0 = ≥1 SD below the mean (84.2%); 1 = <1 SD below the mean (15.8%). |
|  | Subjective hearing function | Study members reported at age 45 on hearing problems via responses to 3 items from the Speech, Spatial, and Qualities of Hearing Scale (SSQ12)^46^ (e.g., “Can you follow the conversation in a busy restaurant?”). Cohort range: 0-29, mean(SD)=7.86(5.12). (N=924 with present data before imputation, 99% of the analytic sample). | Risk points were assigned as: 0 = ≤1 SD above the mean (82.2%); 1 = >1 SD above the mean (17.8%). |
|  | Objective vision function | Contrast sensitivity was measured at age 45 using a Pelli-Robson chart administered by trained technicians. The chart presents 3 letters per line and the letters gradually fade from black to gray to white on a white background to determine the lowest level of “contrast” that the eye can detect. The resulting measure is a contrast sensitivity score function, reflecting a person’s best-corrected contrast detection threshold, the lowest contrast at which a pattern can be seen. Cohort range: 1.4-2.25, mean(SD)=2.00 (0.13). (N=904 with present data before imputation, 96% of the analytic sample). | Risk points were assigned as: 0 = ≥1 SD below the mean (96.1%); 1 = <1 SD below the mean (3.9%). |
|  | Subjective vision function | Study members reported on vision difficulties at age 45 via responses on the 10-item Vision Quality of Life Core Measure (VCM1) questionnaire^47^ (e.g., “How often has your eyesight stopped you from doing the things you wanted to do?”). Cohort range: 0-42, mean(SD)= 3.61 (4.70). (N=925 with present data before imputation, 99% of the analytic sample). | Risk points were assigned as: 0 = ≤1 SD above the mean (88.0%); 1 = >1 SD above the mean (12.0%). |
|  | Poor sense of smell | Study members reported at age 45 on poor sense of smell via response to the question, "Have you had problems with your sense of smell, such as not being able to smell things, or things not smelling the way they should" that had lasted for at least 3 months. (N=931 with present data before imputation, 100% of the analytic sample). | Risk points were assigned as: 0 = No (96.8%); 1 = Yes (3.2%). |
|  |  |  |  |
| Cardio-metabolic function risk^48–53^ | Blood pressure (hypertension) | Systolic and diastolic blood pressure were assessed at age 45 according to standard protocols with a BpTRU™ Vital Signs Monitor BPM 200. (N=906 with present data before imputation, 97% of the analytic sample). | Risk points were assigned based on classification as non-hypertensive (0 [82.0%]) or hypertensive (1 [18.0%]). Study members were considered hypertensive if they had systolic blood pressure 140 mm Hg or greater or diastolic blood pressure 90 mm Hg or greater. |
|  | BMI (weight status) | Height was measured at age 45 using a Seca 264 Wireless Stadiometer. Weight was measured at age 45 to the nearest 0.1 kg using calibrated scales. Individuals were weighed in light clothing. Body mass index (BMI) was calculated. Cohort range: 16.17-62.17, mean(SD)=28.45(5.77). (N=920 with present data before imputation, 98% of the analytic sample). | Risk points were assigned based on classification as non-obese (0 = <30BMI, 66.2%) and obese (1 = ≥30BMI, 33.8%). |
|  | Diabetes status | Whole blood glycated hemoglobin concentration (A1C) (expressed as a percentage of total hemoglobin) was measured at age 45 by ion exchange high performance liquid chromatography (Variant II: BioRad, Hercultes, Calif.), a method certified by the US National Glycohemoglobin Standardization Program (<http://www.ngsp.org/>). (N=876 with present data before imputation, 93% of the analytic sample). | Risk points were assigned based on classification as non-diabetic (0 = <6.5% A1C level, 97.7% of the cohort) and diabetic (1 = ≥6.5% A1C level, 2.3% of cohort). |
|  | Total cholesterol and triglycerides | Serum non-fasting total cholesterol and triglycerides levels were measured at age 45 by colorimetric assay on a Cobas c702 analyzer. (N=879 with present data before imputation, 94% of the analytic sample). | Risk points were assigned based on clinical thresholds for high cholesterol (1 = ≥6.5 mmol/L, 8.1%) and high triglyceridies (1 = >2.26 mmol/L, 33.2%). |
|  | Retinal vascular health | Vascular health was measured at age 45 through assessment of retinal venular vessel calibers. Digital fundus photographs were taken after 15 min of dark adaptation and graded at the SNEC Ocular Reading Centre, Singapore National Eye Centre and Measurements using trained graders following standardized protocols with higher intergrader reliability. Vessel calibers were recorded for arterioles and venules where they passed through a region located 0.50 to 2.00 disk diameters from the optic disk margin and summarized as central retinal artery equivalent (CRAE) and central retinal vein equivalent (CRVE).^54^ Cohort range -2.71 to 3.72, mean(SD) = 0.01(0.98). (N=890 with present data before imputation, 95% of the analytic sample). | Risk points were assigned as: 0 = ≥1 SD below the mean (84.1%); 1 = <1 SD below the mean (15.9%). |
|  |  |  |  |
| Inflammatory risk | CRP level | Serum C-reactive protein (mg/L) was measured at age 45 using particle-enhanced immunoturbidimetric assays on a Cobas c702 analyzer (Roche Diagnostics GmbH) following standard procedures. The lower detection limit of the assay was 0.3 mg/L. The intraassay and interassay CVs reported by the manufacturer were *r* = 0.28–1.34% and *r* = 2.51–5.70%, respectively. Values were log-transformed for analysis to account for positive skew. Cohort range 0-4.51, mean(SD) = 0.97(0.69). (N=879 with present data before imputation, 94% of the analytic sample). | Risk points were assigned as: 0 = ≤1 SD above the mean (85.8%); 1 = >1 SD above the mean (14.2%). |
|  | Il-6 level | Serum IL-6 (pg/mL) was measured at age 45 using an electrochemiluminescence immunoassay on a Cobas e 602 analyzer (Roche Diagnostics GmbH) following standard procedure. The lower detection limit of the assay was 1.5 pg/mL. The intraassay and interassay CVs reported by the manufacturer were 2.5–6.0% and 2.9–8.5%, respectively. Cohort range 0.40-28.47, mean(SD) = 2.18(2.54). (N=876 with present data before imputation, 93% of the analytic sample). | Risk points were assigned as: 0 = ≤1 SD above the mean (93.9%); 1 = >1 SD above the mean (6.1%). |
|  | SuPAR level | Plasma suPAR (ng/mL) was measured at age 45 with the suPARnostic AUTO Flex ELISA (ViroGates A/S, Birkerød, Denmark) according to manufacturer’s instructions. The detection limit of the assay was 0.1 ng/mL. The intraassay correlation of repeat measurements of the same sample was *r* = 0.98 and coefficient of variation (CV) = 2.4%, and the interassay correlation was *r* = 0.81 and CV = 12.8%. Cohort range 0.87-14.37, mean(SD) = 3.07(1.06). (N=875 with present data before imputation, 93% of the analytic sample). | Risk points were assigned as: 0 = ≤1 SD above the mean (89.8%); 1 = >1 SD above the mean (10.2%). |
|  | Rheumatoid arthritis status | Study members reported at age 45 on their history of rheumatoid arthritis during a general health screen. (N=926 with present data before imputation, 99% of the analytic sample). | Risk points were assigned as: 0 = No current arthritis diagnosis (98.2%); 1 = Current arthritis diagnosis (1.8%). |
|  |  |  |  |
| Cellular aging (DNA methylation clock) risk^59,60^ | 4 separate DNA methylation aging clocks | DNA methylation was measured at age 45 as CpG methylation Beta values derived from leukocyte DNA samples using the EPIC array (Illumina Inc., San Diego, California). Methylation values were transformed into four separate DNA methylation “aging” clocks: Horvath,^61^ Hannum,^62^ PhenoAge,^63^ and Grim.^64^ Horvath and Hannum represent first-generation clocks, trained on chronological age in diverse samples. PhenoAge and Grim represent second-generation clocks, trained on phenotypic biomarkers associated with aging (e.g., white blood cell count, albumin levels, etc.). All clocks were calculated using the online calculator found at <https://dnamage.genetics.ucla.edu/new>. ‘Normalization’ and ‘advanced analysis in blood’ options were selected, and data were anonymized prior to upload. From the results file, the corresponding epigenetic age calculations (DNAmAge, DNAmAgeHannum, DNAmPhenoAge, DNAmGrimAge) were extracted. (N=819 with present data before imputation, 87% of the analytic sample). | Risk points were assigned for each DNA methylation clock as: 0 = ≤1 SD above the mean; 1 = >1 SD above the mean.  Horvath: 16.4% at risk  Hannum: 15.4% at risk  PhenoAge: 15.1% at risk  Grim: 16.5% at risk |
|  |  |  |  |
| Harmful events and exposures^65–71^ | Early life lead exposure | Blood-lead level (ug/dL) was assessed at age 11 years. Approximately 30ml of venous blood was collected and whole blood samples analyzed through graphite furnace atomic absorption spectrophotometry. Details on the method of blood collection, storage, and analysis have been previously described.^72,73^ Cohort lead exposure matched that of other same-aged cohorts tested in the United States and United Kingdom.^73^ Cohort range: 4-31, mean(SD) = 10.85(3.61). (N=541 with present data before imputation, 58% of the analytic sample). | Risk points were assigned as: 0 = ≤1 SD above the mean (88.7%); 1 = >1 SD above the mean (11.3%). |
|  | Occupational exposure to pesticides | Study members reported at age 45 on their current occupation. (N=877 with present data before imputation, 93% of the analytic sample). | Risk points were assigned based on occupations that include exposure to pesticides, insecticides, fungicides, or timber preservatives (1 point, 9.9% of cohort) or not (0 points, 90.1 %). |
|  | History of traumatic brain injury | Study member history of traumatic head injury was assessed prospectively by asking at each assessment wave if they experienced a head injury requiring medical attention plus aftercare over the past year. Parents reported on head injury at assessment waves in childhood and adolescence and Study members self-reported at adult assessment waves. (N=938 with present data before imputation, 100% of the analytic sample). | Risk points were assigned as: 0 = no history of traumatic head injury by age 45 (88.5%); 1 = history of traumatic head injury (11.5%). |
|  |  |  |  |
| Subjective Overall Health^74,75^ | Self-rated health | Study members reported at age 45 on their subjective overall health in response to the question, "In general, would you say your health is: excellent, very good, good, fair, poor." Cohort range: 1-5, mean(SD) = 3.66(0.90). (N=931 with present data before imputation, 99% of the analytic sample). | Risk points were assigned as: -1 = very good to excellent (59.3%); 0 = good (31.7%); 1 = fair to poor (9.1%). |
|  | Informant-rated health | Informants who knew the Study members well rated Study member overall health (excellent, very good, good, fair, poor) on questionnaires mailed to them during the age 45 assessment. Cohort range: 1-5, mean(SD) = 3.79(0.89). (N=883 with present data before imputation, 94% of the analytic sample). | Risk points were assigned as: -1 = very good to excellent (67.3%); 0 = good (25.3%); 1 = fair to poor (7.5%). |
|  | Research worker-rated health | Study research workers reported on their subjective impressions of Study member overall health (excellent, very good, good, fair, poor) at the age 45 assessment. Staff ratings were obtained from four raters for each Study member: the cardiovascular nurses, the sensory technicians, the Study Director, and the Assessment Manager (who was in charge of informed consent and logistics on the in-Unit assessment day). Cohort range: 1-5, mean(SD) = 3.36(0.84). (N=934 with present data before imputation, 99% of the analytic sample). | Risk points were assigned as: -1 = very good to excellent (47.7%); 0 = good (39.1%); 1 = fair to poor (13.3%). |
|  |  |  |  |

*Note.* Indicators were present for 93-100% of the analytic sample for all indicators except DNA methylation clocks and childhood blood-lead level, which were available for only 87% and 58% of the cohort respectively; missing indicator values were imputed to create complete indicator data for the entire cohort present at the age-45 wave. Removing the lead-level indicator did not change the results.

^a^Representative empirical studies, meta-analyses, systematic reviews, and narrative reviews are cited to describe the evidence of associations of known or proposed risk factors within the 10 risk domains with ADRD. These are cited for expository purposes and are not meant to reflect all the evidence available on a given risk domain, factor, or indicator.

^b^Risk points were assigned after imputation to address missing data. Risk point assignments were not weighted as meta-analyses were not available for all risk indicators. The Dunedin ARI construction allowed each risk domain to contribute equally to the overall risk score.

# Supplementary Table 2. Description of the midlife brain integrity measures.

|  |  | **Description** |
| --- | --- | --- |
| **Brain structural integrity measures** | | |
|  | BrainAGE, years | At age 45, participants completed a neuroimaging magnetic resonance imaging (MRI) protocol including assessment of brain structure with T1-weighted, fluid-attenuated inversion recovery, and diffusion-weighted sequences using a Siemens Skyra 3T scanner (Siemens Healthcare) equipped with a 64-channel head and neck coil.  High-resolution structural images were used to generate estimates of Study member brainAGE^76^, a composite neuroimaging biomarker of age-related brain structure, which estimates aging-related variance in diverse brains structures from cross-sectional brain measures. brainAGE is estimated by training machine-learning algorithms to predict chronological age from structural data collected in large samples of individuals across a broad age range. Using a publicly available algorithm brainAGE was estimated from surface area, cortical thickness, and subcortical volume data to produce a brain-age-gap estimate, which is the difference between an individual’s predicted age based on MRI data and their chronological age. Positive brainAGE values suggest that the brain is morphologically “older” than an individual’s chronological age would predict.  Analytic sample N = 869, M (SD) = 0.00 (8.01) years |
|  | White Matter Hyperintensities Volume, log mm^3^ | Total volume of white matter hyperintensities^77^ was measured using the Unidentified Bright Objects (UBO) algorithm. White matter hyperintensities are bright spots on a T2 weighted MRI scan that are a sign of deterioration of white matter. The variable was log-transformed to produce an approximately normal distribution.  Analytic sample N = 852, M (SD) = 6.52 (0.80) log mm^3^ |
|  | Hippocampal Volume, mm^3^ | Total bilateral volume of the hippocampus^78^, a region of the brain important for learning and memory, was measured using FreeSurfer’s automated segmentation (ASEG) algorithm.  Analytic sample N = 861, M (SD) = 86.45 (8.5) mm^3^ |
|  |  |  |
| **Brain functional integrity measures** | | |
|  | Objective Cognitive Function | Objective cognitive function was assessed using the Wechsler Adult Intelligence Scale–IV (WAIS-IV; potential score range 40-160; standardized in the full cohort to mean[SD]=100[15]) at age 45 years to produce the overall full-scale IQ. Higher scores indicate greater cognitive performance.  Analytic sample, N = 918, M mean(SD) = 100 (15). |
|  | Subjective Cognitive Problems | Subjective everyday cognitive function was reported on by the Study members (self-report) and by individuals who knew the Study members well (informant-report). With matched checklist items, the Study members were interviewed by trained Study staff and the informants were mailed questionnaires. Both were asked about whether the Study members had problems with their memory or attention over the past year, including questions such as whether they were more likely to be “easily distracted” and “get sidetracked” as well as to “misplace wallet, keys or eyeglasses” and “forget to do errands, return calls or pay bills.” Self and informant reports were correlated at r=.38, *p*<.001. Self and informant memory and attention scales were z-scored and averaged to produce an overall subject scale of problems with everyday cognitive function.  Analytic sample, N = 921, M (SD) = 0.01 (0.77). |
|  | Cognitive Decline | Cognitive change (decline) was assessed as change in objective cognitive function from childhood to midlife measured by studying IQ scores at midlife after controlling for IQ scores in childhood (“residualized change”). The Wechsler Intelligence Scale for Children–Revised (WISC– ; potential score range 40-160; standardized in the full cohort to mean(SD)=100(15)]) yielding the full-scale IQ was administered to each participant at ages 7, 9, and 11 years, with scores from all years averaged together to increase baseline reliability.  Analytic sample, N = 906, M (SD) = 0.00 (9.55). |

*Note.* SD = standard deviation.

# Supplementary Table 3. Assignment of risk points and weighting for each risk factor in the four published ADRD indices.

| **1**. **The** **CAIDE risk index**^5^ | | | | |
| --- | --- | --- | --- | --- |
|  | **Indicator** | **Level** | **Risk Points** |  |
|  | Age |  |  |  |
|  |  | <47 years old | 0 |  |
|  |  | 47-53 years old | 3 |  |
|  |  | >53 years old | 5 |  |
|  | Education |  |  |  |
|  |  | ≥10 years | 0 |  |
|  |  | 7-9 years | 3 |  |
|  |  | 0-6 years | 4 |  |
|  | Sex |  |  |  |
|  |  | Female | 0 |  |
|  |  | Male | 1 |  |
|  | Hypertension |  |  |  |
|  |  | SBP ≤ 140 mmHg | 0 |  |
|  |  | SBP >140 mmHg | 2 |  |
|  | Obesity (BMI) |  |  |  |
|  |  | ≤30 kg/m2 BMI | 0 |  |
|  |  | >30 kg/m2 BMI | 2 |  |
|  | Total cholesterol | |  |  |
|  |  | ≤6.5 mmol/l total cholesterol | 0 |  |
|  |  | >6.5 mmol/l cholesterol | 1 |  |
|  | Physical activity | |  |  |
|  |  | Active: physical activity at least twice a week, lasting at least 20–30 min each time, and causing sweating and breathlessness. | 0 |  |
|  |  | Inactive | 1 |  |
|  | APOE ε4 allele status |  |  |  |
|  |  | Non-carrier | 0 |  |
|  |  | Carrier of at least 1 ε4 allele | 2 |  |
|  |  |  |  |  |
|  |  | **Potential Total Points Range** | 0 to 18 |  |
|  |  | **Dunedin Study Total Points Range** | 0 to 13 |  |

| **2. The LIBRA risk index**^6^ | | |  |
| --- | --- | --- | --- |
|  | **Indicator** | **Level** | **Risk Points** |
|  | Hypertension |  |  |
|  |  | Not hypertensive | 0 |
|  |  | SBP ≥ 140 mmHg or DBP ≥ 90 mmHg | 1.6 |
|  | Obesity (BMI) |  |  |
|  |  | <30 kg/m2 BMI | 0 |
|  |  | ≥30 kg/m2 BMI | 1.6 |
|  | High cholesterol |  |  |
|  |  | <6.5 mmol/l total cholesterol | 0 |
|  |  | ≥6.5 mmol/l total cholesterol | 1.4 |
|  | Diabetes |  |  |
|  |  | Not diagnosed | 0 |
|  |  | Diagnosed | 1.3 |
|  | Coronary heart disease | |  |
|  |  | No disease | 0 |
|  |  | Myocardial infarction, angina, ischemic heart disease, or atrial fibrillation reported | 1 |
|  | Chronic kidney disease | |  |
|  |  | No disease | 0 |
|  |  | Chronic nephritis, chronic renal failure, and proteinuria reported | 1.1 |
|  | Physical inactivity | |  |
|  |  | Active: At least 20-30 minutes of daily or 2-3x weekly physical activity causing breathlessness and sweating. | 0 |
|  |  | Inactive | 1.1 |
|  | Low/moderate alcohol intake | |  |
|  |  | Consumes alcohol once every 2 months or less | -1 |
|  |  | Consumes alcohol once a month or more / Does not drink | 0 |
|  | Smoking |  |  |
|  |  | Never smoker | 0 |
|  |  | Ever smoker | 1.5 |
|  | Depression |  |  |
|  |  | Did not report feeling somewhat or more "hopeless" on questionnaire | 0 |
|  |  | Reported feeling somewhat or more "hopeless" on questionnaire | 2.1 |
|  | Healthy diet |  |  |
|  |  | Low adherence to a healthy diet (≤8 on a 17 point scale) | 0 |
|  |  | high adherence to a healthy diet (>8 on a 17 point scale) | -1.7 |
|  | Cognitive and social engagement^a†^ | |  |
|  |  | Self-reported engagement in intellectual and social activities | 0 |
|  |  | Self-reported engagement in intellectual and social activities | -3.2 |
|  |  |  |  |
|  |  | **Potential Total Points Range** | -5.9 to 12.7 |
|  |  | **Dunedin Study Total Points Range** | -2.7 to 12.7 |

| **3. The Lancet Commission**^2^ **risk factor list^b^** | | | |  |
| --- | --- | --- | --- | --- |
|  | **Indicator** | **Level** | **Risk Points** | |
|  | Education |  |  | |
|  |  | “More education”  (Operationalized as: high school graduate or above) | 0 | |
|  |  | “Less education”  (Operationalized as: left school without certification or high school degree) | 1.6 | |
|  | Hearing loss |  |  | |
|  |  | No hearing loss or hearing lose with use of a hearing aid | 0 | |
|  |  | Hearing loss without use of hearing aid | 1.9 | |
|  | Traumatic Head Injury |  |  | |
|  |  | No documented history of traumatic head injury | 0 | |
|  |  | History of traumatic head injury | 1.8 | |
|  | Hypertension | |  | |
|  |  | Not hypertensive | 0 | |
|  |  | SBP ≥ 140 mmHg or DBP ≥ 90 mmHg | 1.6 | |
|  | Excessive alcohol consumption | |  | |
|  |  | < 21 units alcohol consumed per week | 0 | |
|  |  | ≥ 21 units consumed per week | 1.2 | |
|  | Obesity (BMI) |  |  | |
|  |  | <30 kg/m2 BMI | 0 | |
|  |  | ≥30 kg/m2 BMI | 1.6 | |
|  | Smoking |  |  | |
|  |  | Non-smoker by midlife | 0 | |
|  |  | Current smoker by midlife | 1.6 | |
|  | Depression |  |  | |
|  |  | Never received a diagnosis by midlife | 0 | |
|  |  | Diagnosed episode by midlife | 1.9 | |
|  | Social isolation | |  | |
|  |  | Not socially isolated  (Operationalized as ≤1 SD above the cohort mean on a scale assessing loneliness) | 0 | |
|  |  | Socially isolated  (Operationalized as >1 SD above the cohort mean on a scale assessing loneliness) | 1.6 | |
|  | Physical activity | |  | |
|  |  | Active | 0 | |
|  |  | Inactive | 1.4 | |
|  | Diabetes |  |  | |
|  |  | No diagnosis | 0 | |
|  |  | Diagnosed | 1.5 | |
|  | Air pollution exposure | |  | |
|  |  | Low exposure | 0 | |
|  |  | High exposure^c^ | 1.1 | |
|  |  |  |  | |
|  |  | **Potential Total Points Range** | 0 to 18 | |
|  |  | **Dunedin Study Total Points Range** | 0 to 18 | |

| **4. The ANU-ADRI risk index**^10^ | | | |
| --- | --- | --- | --- |
|  | **Indicator** | **Level** | **Risk Points** |
|  | Age for males | |  |
|  |  | <65 years | 0 |
|  |  | ≥65 | 1 to 38 points |
|  | Age for females | |  |
|  |  | <65 years | 0 |
|  |  | ≥65 | 1 to 41 points |
|  | Education |  |  |
|  |  | > 11 years | 0 |
|  |  | 8-11 years | 3 |
|  |  | <8 years | 6 |
|  | Weight status if age <60 | |  |
|  |  | Reported as normal | 0 |
|  |  | Reported as overweight | 2 |
|  |  | Reported as obese | 5 |
|  | Diabetes |  |  |
|  |  | No diabetes reported | 0 |
|  |  | Diabetes reported | 3 |
|  | Symptoms of Depression | |  |
|  |  | CES-D score ≤ 16 | 0 |
|  |  | CES-D score > 16 | 2 |
|  | High cholesterol | |  |
|  |  | <6.2 mmol/l total cholesterol | 0 |
|  |  | ≥6.2 mmol/l total cholesterol | 3 |
|  | Traumatic Brain Injury |  |  |
|  |  | No history of TBI | 0 |
|  |  | Positive history of TBI | 4 |
|  | Smoking |  |  |
|  |  | Never smoker | 0 |
|  |  | Ever smoker | 1 |
|  |  | Current smoker | 4 |
|  | Alcohol intake | |  |
|  |  | No alcohol consumed | 0 |
|  |  | Light to moderate consumption | -3 |
|  |  | Heavy consumption | 0 |
|  | Social engagement^e^ | |  |
|  |  | Highest scores in sample | 0 |
|  |  | Lowest scores in sample | 6 |
|  |  | Low to medium scores in sample | 4 |
|  |  | Medium to high scores in sample | 1 |
|  | Physical activity | |  |
|  |  | High IPAQ score | 0 |
|  |  | Moderate IPAQ score | -2 |
|  |  | Low IPAQ score | -3 |
|  | Cognitive activity^f†^ | |  |
|  |  | Lowest cogntive activity questionnaire scores | 0 |
|  |  | Middle scores | -7 |
|  |  | Highest scores | -6 |
|  | Fish intake |  |  |
|  |  | <.25 servings per week | 0 |
|  |  | .25-2 servings per week | -3 |
|  |  | 2-4 servings per week | -4 |
|  |  | >4 servings per week | -5 |
|  | Pesticide exposure | |  |
|  |  | Never | 0 |
|  |  | Ever | 2 |
|  |  |  |  |
|  |  | **Potential Total Points Range** | -18 to 35 |
|  |  | **Dunedin Study Total Points Range** | -10 to 23 |

*Note*. BMI = Body Mass Index; SBP = Systolic Blood Pressure; DBP = Diastolic Blood Pressure; TBI = Traumatic Brain Injury. CES-D = Center for Epidemiologic Studies Depression scale (CES-D). IPAQ = the International Physical Activity Questionnaire

^†^This measure was not available in the Dunedin Cohort.

^a^Self-reported engagement in intellectual and social activities in the last 12 months (e.g., read the newspaper on a daily basis, have a hobby, take a holiday, using the internet, being a member of any organizations).

^b^At the time of study there were no previous publications assessing risk prediction using the Lancet Comission risk factor list. Lancet Index cut-points were consequently developed based on existing clinical thresholds (e.g., for weight status and hypertension), the presence of categorical conditions (e.g., depression diagnosis), cut-points used by other indices (e.g., for physical activity), and extreme scores on continuous measures relative to cohort peers (e.g., for social isolation). Risk scores were assigned based on the Lancet Commission’s published relative risk for dementia scores for each risk indicator (Table 1, Column 1 in Livingston et al., 2020).^2^

^c^High air pollution exposure was operationalized as residence for one year or more in a poor air quality city (i.e., top 500 worst air quality cities as ranked by IQAir, www.iqair.com) or a country with average annual outdoor ambiet air quality below World Health Organization standards.

^e^Social engagement in the ANU-ADRI development was measured via a composite score encompassing marital status, size and quality of social networks, level of social activities and living arrangements. It was operationalized in the Dunedin cohort as reverse coded high (≥ 6), medium to high (2.1 to 5), low to medium (0.1 to 2), and low (0) scores on the UCLA Loneliness Scale.^36^

^f^ANU-ADRI utilized a modified form of the Rush Memory and Aging Study cogntive activity questionnaire and assigned risk points based on the distribution of scores within the test sample.

# Supplementary Table 4. Correlations among individual risk indicators within the 10 classes of risk comprising the DunedinARB.

| **Risk class** | **Risk factors** | **Correlations**  **(Pearson’s r or Spearman’s *ρ*)** | | | | | |
| --- | --- | --- | --- | --- | --- | --- | --- |
|  |  | **1** | **2** |  |  |  |  |
| Genetic risk | 1. Family history of dementia | --- |  |  |  |  |  |
|  | 2. APOE e4 allele status | .10** | --- |  |  |  |  |
|  |  | **1** | **2** | **3** | **4** | **5** | **6** |
| Lifestyle risk | 1. Physical activity^†^ | --- |  |  |  |  |  |
|  | 2. Mediterranean Diet^†^ | .28*** | --- |  |  |  |  |
|  | 3. Tobacco smoking | -.11** | -.22*** | --- |  |  |  |
|  | 4. Alcohol consumption | .04 | .05 | .15*** | --- |  |  |
|  | 5. Folic acid supplementation^†^ | .04 | -.03 | .02 | -.01 | --- |  |
|  | 6. Regular prophylactic NSAID use^†^ | .01 | -.01 | -.02 | -.02 | .05 | --- |
|  |  | **1** | **2** |  |  |  |  |
| Socio-economic risk | 1. Occupational attainment^†^ | --- |  |  |  |  |  |
|  | 2. Educational attainment^†^ | .61*** | --- |  |  |  |  |
|  |  | **1** | **2** | **3** | **4** | **5** | **6** |
| Psychosomatic risk | 1. Pain interference with daily function | --- |  |  |  |  |  |
|  | 2. History of migraine | .19*** | --- |  |  |  |  |
|  | 3. History of depression | .16*** | .23*** | --- |  |  |  |
|  | 4. Social isolation | .12*** | .13*** | .39*** | --- |  |  |
|  | 5. Poor Sleep quality | .13*** | .09** | .20*** | .25*** | --- |  |
|  | 6. Neuroticism | .07* | .13*** | .33*** | .28*** | .19*** | --- |
|  | 7. Conscientiousness^†^ | -.15*** | -.03 | -.13*** | -.22*** | -.15*** | -.20*** |
|  |  |  |  |  |  |  |  |
|  |  |  |  |  |  |  |  |
|  |  |  |  |  |  |  |  |
|  |  |  |  |  |  |  |  |
|  |  |  |  |  |  |  |  |
|  |  | **1** | **2** | **3** | **4** | **5** | **6** |
| Physical and Sensory function risk | 1. Balance^†^ | --- |  |  |  |  |  |
|  | 2. Gait^†^ | .27*** | --- |  |  |  |  |
|  | 3. Objective hearing function | .11*** | .14*** | --- |  |  |  |
|  | 4. Subjective hearing function^†^ | .12*** | .18*** | .20*** | --- |  |  |
|  | 5. Objective vision function^†^ | .12*** | .12*** | .02 | .14*** | --- |  |
|  | 6. Subjective vision function^†^ | .10*** | .11*** | .05 | .20*** | .21*** | --- |
|  | 7. Poor sense of smell | .00 | .00 | -.07* | -.05 | -.06 | .00 |
|  |  | **1** | **2** | **3** | **4** | **5** | **6** |
| Cardio-metabolic status risk | 1. Blood pressure (hypertension) | --- |  |  |  |  |  |
|  | 2. BMI (weight status) | .25*** | --- |  |  |  |  |
|  | 3. Diabetes status | .09** | .26*** | --- |  |  |  |
|  | 4. Cholesterol level | .14*** | .11*** | .07** | --- |  |  |
|  | 5. Triglyceride level | .19*** | .29*** | .28*** | .38*** | --- |  |
|  | 6. Retinal vascular health^†^ | -.15*** | -.13*** | -.09* | -.04 | -.13*** | --- |
|  |  | **1** | **2** | **3** | **4** |  |  |
| Inflamma-tory risk | 1. C-reactive protein level | --- |  |  |  |  |  |
|  | 2. Interleukin 6 level | .45*** | --- |  |  |  |  |
|  | 3. SuPAR level | .27*** | .16*** | --- |  |  |  |
|  | 4. Rheumatoid arthritis | .05 | ..04 | .06 | --- |  |  |
|  |  | **1** | **2** | **3** | **4** |  |  |
| Epigenetic cellular aging risk | 1. Horvath clock | --- |  |  |  |  |  |
|  | 2. Hannum clock | .51*** | --- |  |  |  |  |
|  | 3. PhenoAge clock | .45*** | .43*** | --- |  |  |  |
|  | 4. Grim clock | .11*** | .21*** | .37*** | --- |  |  |
|  |  | **1** | **2** | **3** |  |  |  |
| Harmful events and exposures | Early life lead exposure | --- |  |  |  |  |  |
|  | Occupational exposure to pesticides | .04 | --- |  |  |  |  |
|  | History of traumatic head injury | .02 | .07* | --- |  |  |  |
|  |  | **1** | **2** | **3** |  |  |  |
| Subjective Overall Health | Self-rated health^†^ | --- |  |  |  |  |  |
|  | Informant-rated health^†^ | .46*** | --- |  |  |  |  |
|  | Research worker-rated health^†^ | .48*** | .51*** | --- |  |  |  |

*Note*: *p-values <.05, **p-values <.01, ***p-values <.001. Correlations calculated using raw, non-dichotomous indicator variables where relevant. ^†^ Indicators where higher scores indicate lower putative ADRD risk (e.g., higher self-rated health indicates better health, higher physical activity indicates greater weekly physical activity).

# Supplementary Table 5. Association of the 10 domains of risk comprising the DunedinARB with the midlife measures of brain integrity.

|  | **Structural integrity (MRI) measures** | | |  | **Functional integrity (cognitive) measures** | | |
| --- | --- | --- | --- | --- | --- | --- | --- |
|  | **brainAGE** | **Log WMH volume** | **Hippocampal volume** |  | **Objective midlife IQ** | **Subjective midlife cognitive problems** | **IQ change from childhood** |
|  | **β** | | |  | **β** | | |
| **1. Genetic risk** | -.04 | .04 | .01 |  | -.02 | .00 | -.02 |
| **2. Lifestyle risk** | .18*** | .05 | -.08* |  | -.29*** | .14*** | -.14*** |
| **3. Social class risk** | .18*** | .16*** | -.14*** |  | -.60*** | .24*** | -.30*** |
| **4. Psycho-somatic risk** | .15*** | .11** | -.05 |  | -.18*** | .45*** | -.11*** |
| **5. Physical & sensory risk** | .10** | .12*** | -.05 |  | -.37*** | .28*** | -.18*** |
| **6. Cardio-metabolic risk** | .09* | .15*** | -.00 |  | -.10** | .02 | -.01 |
| **7. Inflammatory risk** | .11** | .09** | -.03 |  | -.19*** | .15*** | -.08* |
| **8. DNA methylation aging risk** | .12*** | .08* | -.05 |  | -.20*** | .05 | -.17*** |
| **9. Harmful events risk** | .03 | .02 | -.04 |  | -.12*** | .13*** | -.08* |
| **10. Subjective health risk** | .15*** | .13*** | -.07* |  | -.31*** | .35*** | -.19*** |

*Note*. All associations adjusted for sex. *p-values <.05, **p-values <.01, ***p-values <.001.

# Supplementary Table 6. Association of the 10 domains of risk comprising the DunedinARB with longitudinal cognitive decline over and above the 4 published risk indices, adjusted for sex.

| **Domain of Risk** | Association over & above  the CAIDE | | Association over & above  the LIBRA | | Association over & above  the LANCET | | Association over & above  the ANU-ADRI | |
| --- | --- | --- | --- | --- | --- | --- | --- | --- |
|  | β | (95% CI) | β | (95% CI) | β | (95% CI) | β | (95% CI) |
| 1. Genetic | 0.05 | (-0.02, 0.12) | -0.02 | (-0.08, 0.04) | -0.01 | (-0.07, 0.06) | -0.01 | (-0.08, 0.05) |
| 2. Lifestyle | **-0.09** | **(-0.16, -0.02)** | -0.08 | (-0.16, 0.00) | **-0.08** | **(-0.16, -0.00)** | -0.03 | (-0.11, 0.06) |
| **3. Socioeconomic** | **-0.30** | **(-0.38, -0.23)** | **-0.30** | **(-0.38, -0.23)** | **-0.30** | **(-0.37, -0.23)** | **-0.28** | **(-0.36, -0.21)** |
| 4. Psycho-somatic | **-0.09** | **(-0.16, -0.02)** | -0.07 | (-0.14, 0.00) | -0.05 | (-0.13, 0.02) | -0.03 | (-0.10, 0.04) |
| **5. Physio-sensory** | **-0.14** | **(-0.21, -0.08)** | **-0.14** | **(-0.21, -0.08)** | **-0.14** | **(-0.21, -0.06)** | **-0.12** | **(-0.19, -0.06)** |
| 6. Cardio-metabolic | **0.09** | **(0.02, 0.17)** | 0.07 | (-0.00, 0.14) | 0.05 | (-0.02, 0.13) | **0.07** | **(0.00, 0.14)** |
| 7. Inflammatory | -0.04 | (-0.11, 0.03) | -0.04 | (-0.11, 0.03) | -0.04 | (-0.11, 0.03) | -0.02 | (-0.09, 0.05) |
| **8. Epigenetic** | **-0.14** | **(-0.20, -0.07)** | **-0.14** | **(-0.21, -0.08)** | **-0.14** | **(-0.21, -0.07)** | **-0.13** | **(-0.19, -0.06)** |
| 9. Harmful events | **-0.07** | **(-0.14, -0.01)** | -0.07 | (-0.13, 0.00) | -0.05 | (-0.12, 0.02) | -0.03 | (-0.10, 0.03) |
| **10. Overall health** | **-0.14** | **(-0.21, -0.07)** | **-0.15** | **(-0.22, -0.08)** | **-0.15** | **(-0.23, -0.08)** | **-0.12** | **(-0.19, -0.04)** |

*Note*. 95% CI = 95% confidence interval. Red highlights indicate risk domains that are significantly associated with cognitive decline over and above the published indices (i.e., in models also including the published risk index).

# Supplementary References

1. Daviglus ML, Plassman BL, Pirzada A, et al. Risk factors and preventive interventions for Alzheimer disease: State of the science. *Arch Neurol*. 2011;68(9):1185-1190. doi:10.1001/archneurol.2011.100

2. Livingston G, Huntley J, Sommerlad A, et al. Dementia prevention, intervention, and care: 2020 report of the Lancet Commission. *The Lancet*. 2020;396(10248):413-446. doi:10.1016/S0140-6736(20)30367-6

3. Vuorinen M, Spulber G, Damangir S, et al. Midlife CAIDE dementia risk score and dementia-related brain changes up to 30 years later on Magnetic Resonance imaging. *J Alzheimers Dis*. 2015;44(1):93-101. doi:10.3233/JAD-140924

4. Exalto LG, Quesenberry CP, Barnes D, Kivipelto M, Biessels GJ, Whitmer RA. Midlife risk score for the prediction of dementia four decades later. *Alzheimers Dement*. 2014;10(5):562-570. doi:10.1016/j.jalz.2013.05.1772

5. Kivipelto M, Ngandu T, Laatikainen T, Winblad B, Soininen H, Tuomilehto J. Risk score for the prediction of dementia risk in 20 years among middle aged people: a longitudinal, population-based study. *Lancet Neurol*. 2006;5(9):735-741. doi:10.1016/S1474-4422(06)70537-3

6. Vos SJB, van Boxtel MPJ, Schiepers OJG, et al. Modifiable risk Factors for prevention of dementia in midlife, late life and the oldest-old: Validation of the LIBRA index. *J Alzheimers Dis*. 2017;58(2):537-547. doi:10.3233/JAD-161208

7. Schiepers OJG, Köhler S, Deckers K, et al. Lifestyle for Brain Health (LIBRA): a new model for dementia prevention. *Int J Geriatr Psychiatry*. 2018;33(1):167-175. doi:10.1002/gps.4700

8. Deckers K, van Boxtel MPJ, Schiepers OJG, et al. Target risk factors for dementia prevention: a systematic review and Delphi consensus study on the evidence from observational studies. *Int J Geriatr Psychiatry*. 2015;30(3):234-246. doi:10.1002/gps.4245

9. Anstey KJ, Cherbuin N, Herath PM, et al. A self-report risk index to predict occurrence of dementia in three independent cohorts of older adults: The ANU-ADRI. *PLOS ONE*. 2014;9(1):e86141. doi:10.1371/journal.pone.0086141

10. Anstey KJ, Cherbuin N, Herath PM. Development of a new method for assessing global risk of Alzheimer’s disease for use in population health approaches to prevention. *Prev Sci*. 2013;14(4):411-421. doi:10.1007/s11121-012-0313-2

11. Low LF, Harrison F, Lackersteen SM. Does personality affect risk for dementia? A systematic review and meta-analysis. *Am J Geriatr Psychiatry*. 2013;21(8):713-728. doi:10.1016/j.jagp.2012.08.004

12. Aschwanden D, Strickhouser JE, Luchetti M, Stephan Y, Sutin AR, Terracciano A. Is personality associated with dementia risk? A meta-analytic investigation. *Ageing Res Rev*. 2021;67:101269. doi:10.1016/j.arr.2021.101269

13. Huang W, Qiu C, von Strauss E, Winblad B, Fratiglioni L. APOE genotype, family history of dementia, and Alzheimer disease risk: A 6-year follow-up study. *Arch Neurol*. 2004;61(12):1930-1934. doi:10.1001/archneur.61.12.1930

14. Rawle MJ, Davis D, Bendayan R, Wong A, Kuh D, Richards M. Apolipoprotein-E (Apoe) ε4 and cognitive decline over the adult life course. *Transl Psychiatry*. 2018;8(1):1-8. doi:10.1038/s41398-017-0064-8

15. Walesby KE, Exeter DJ, Gibb S, Wood PC, Starr JM, Russ TC. Prevalence and geographical variation of dementia in New Zealand from 2012 to 2015: Brief report utilising routinely collected data within the Integrated Data Infrastructure. *Australas J Ageing*. 2020;39(3):297-304. doi:10.1111/ajag.12790

16. Freedman VA, Kasper JD. Cohort Profile: The National Health and Aging Trends Study (NHATS). *Int J Epidemiol*. 2019;48(4):1044-1045g. doi:10.1093/ije/dyz109

17. ALzGene. Meta-analysis of all published AD association studies (case-control only) APOE E2/3/4. Published 2010. Accessed May 25, 2022. http://www.alzgene.org/meta.asp?geneID=83

18. Scarmeas N, Luchsinger JA, Schupf N, et al. Physical activity, diet, and risk of Alzheimer disease. *JAMA J Am Med Assoc*. 2009;302(6):627-637. doi:10.1001/jama.2009.1144

19. Tyndall AV, Clark CM, Anderson TJ, et al. Protective effects of exercise on cognition and brain health in older adults. *Exerc Sport Sci Rev*. 2018;46(4):215-223. doi:10.1249/JES.0000000000000161

20. Durazzo TC, Mattsson N, Weiner MW. Smoking and increased Alzheimer’s disease risk: A review of potential mechanisms. *Alzheimers Dement J Alzheimers Assoc*. 2014;10(3 0):S122-S145. doi:10.1016/j.jalz.2014.04.009

21. Ballarini T, Schröder A, Lent DM van, et al. The effects of Mediterranean diet on memory and Alzheimer’s disease biomarkers. *Alzheimers Dement*. 2020;16(S10):e045349. doi:https://doi.org/10.1002/alz.045349

22. Lefèvre-Arbogast S, Féart C, Dartigues JF, Helmer C, Letenneur L, Samieri C. Dietary B vitamins and a 10-year risk of dementia in older persons. *Nutrients*. 2016;8(12):761. doi:10.3390/nu8120761

23. Szekely CA, Breitner JCS, Fitzpatrick AL, et al. NSAID use and dementia risk in the Cardiovascular Health Study: Role of APOE and NSAID type. *Neurology*. 2008;70(1):17-24. doi:10.1212/01.wnl.0000284596.95156.48

24. World Health Organization. *WHO Guidelines on Physical Activity and Sedentary Behaviour*.; 2020.

25. Seblova D, Fischer M, Fors S, et al. Does prolonged education causally affect dementia risk when adult socioeconomic status is not altered? A Swedish natural experiment in 1.3 million individuals. *Am J Epidemiol*. 2021;190(5):817-826. doi:10.1093/aje/kwaa255

26. Russ TC, Stamatakis E, Hamer M, Starr JM, Kivimäki M, Batty GD. Socioeconomic status as a risk factor for dementia death: individual participant meta-analysis of 86 508 men and women from the UK. *Br J Psychiatry*. 2013;203(1):10-17. doi:10.1192/bjp.bp.112.119479

27. Sharp ES, Gatz M. The relationship between education and dementia an updated systematic review. *Alzheimer Dis Assoc Disord*. 2011;25(4):289-304. doi:10.1097/WAD.0b013e318211c83c

28. Richmond-Rakerd LS, D’Souza S, Andersen SH, et al. Clustering of health, crime and social-welfare inequality in 4 million citizens from two nations. *Nat Hum Behav*. Published online January 20, 2020:1-10. doi:10.1038/s41562-019-0810-4

29. Sabia S, Fayosse A, Dumurgier J, et al. Association of sleep duration in middle and old age with incidence of dementia. *Nat Commun*. 2021;12(1):2289. doi:10.1038/s41467-021-22354-2

30. Zilkens RR, Bruce DG, Duke J, Spilsbury K, Semmens JB. Severe psychiatric disorders in mid-Life and risk of dementia in late-life (Age 65-84 Years): A population based case-control Study. *Curr Alzheimer Res*. 2014;11(7):681-693. doi:10.2174/1567205011666140812115004

31. Spira AP, Chen-Edinboro LP, Wu MN, Yaffe K. Impact of sleep on the risk of cognitive decline and dementia. *Curr Opin Psychiatry*. 2014;27(6):478-483. doi:10.1097/YCO.0000000000000106

32. Chuang CS, Lin CL, Lin MC, Sung FC, Kao CH. Migraine and risk of dementia: A nationwide retrospective cohort study. *Neuroepidemiology*. 2013;41(3-4):139-145. doi:10.1159/000353559

33. Islamoska S, Hansen ÅM, Wang HX, et al. Mid- to late-life migraine diagnoses and risk of dementia: a national register-based follow-up study. *J Headache Pain*. 2020;21(1):98. doi:10.1186/s10194-020-01166-7

34. Headache Classification Committee of the International Headache Society. Classification and diagnostic criteria for headache disorders, cranial neuralgias and facial pain. *Cephalalgia*. 1988;8 Suppl 7:1-96.

35. Waldie KE, Hausmann M, Milne BJ, Poulton R. Migraine and cognitive function: a life-course study. *Neurology*. 2002;59(6):904-908. doi:10.1212/wnl.59.6.904

36. Russell D, Peplau LA, Cutrona CE. The revised UCLA Loneliness Scale: Concurrent and discriminant validity evidence. *J Pers Soc Psychol*. 1980;39(3):472-480. doi:10.1037/0022-3514.39.3.472

37. Buysse DJ, Reynolds CF, Monk TH, Berman SR, Kupfer DJ. The Pittsburgh Sleep Quality Index: a new instrument for psychiatric practice and research. *Psychiatry Res*. 1989;28(2):193-213. doi:10.1016/0165-1781(89)90047-4

38. Benet-Martínez V, John OP. Los Cinco Grandes across cultures and ethnic groups: multitrait multimethod analyses of the Big Five in Spanish and English. *J Pers Soc Psychol*. 1998;75(3):729-750.

39. Verghese J, Lipton RB, Hall CB, Kuslansky G, Katz MJ, Buschke H. Abnormality of gait as a predictor of non-Alzheimer’s dementia. *N Engl J Med*. 2002;347(22):1761-1768. doi:10.1056/NEJMoa020441

40. Thomson RS, Auduong P, Miller AT, Gurgel RK. Hearing loss as a risk factor for dementia: A systematic review. *Laryngoscope Investig Otolaryngol*. 2017;2(2):69-79. doi:10.1002/lio2.65

41. Paik JS, Ha M, Jung YH, et al. Low vision and the risk of dementia: a nationwide population-based cohort study. *Sci Rep*. 2020;10(1):9109. doi:10.1038/s41598-020-66002-z

42. Bathini P, Brai E, Auber LA. Olfactory dysfunction in the pathophysiological continuum of dementia. *Ageing Res Rev*. 2019;55:100956. doi:10.1016/j.arr.2019.100956

43. Verghese J, Wang C, Lipton RB, Holtzer R, Xue X. Quantitative gait dysfunction and risk of cognitive decline and dementia. *J Neurol Neurosurg Psychiatry*. 2007;78(9):929-935. doi:10.1136/jnnp.2006.106914

44. Naël V, Pérès K, Dartigues JF, et al. Vision loss and 12-year risk of dementia in older adults: the 3C cohort study. *Eur J Epidemiol*. 2019;34(2):141-152. doi:10.1007/s10654-018-00478-y

45. Springer BA, Marin R, Cyhan T, Roberts H, Gill NW. Normative values for the unipedal stance test with eyes open and closed. *J Geriatr Phys Ther 2001*. 2007;30(1):8-15. doi:10.1519/00139143-200704000-00003

46. Rasmussen LJH, Caspi A, Ambler A, et al. Association of neurocognitive and physical function with gait speed in midlife. *JAMA Netw Open*. 2019;2(10):e1913123. doi:10.1001/jamanetworkopen.2019.13123

47. Noble W, Jensen NS, Naylor G, Bhullar N, Akeroyd MA. A short form of the Speech, Spatial and Qualities of Hearing scale suitable for clinical use: the SSQ12. *Int J Audiol*. 2013;52(6):409-412. doi:10.3109/14992027.2013.781278

48. Frost NA, Sparrow JM, Durant JS, Donovan JL, Peters TJ, Brookes ST. Development of a questionnaire for measurement of vision-related quality of life. *Ophthalmic Epidemiol*. 1998;5(4):185-210. doi:10.1076/opep.5.4.185.4191

49. Exalto LG, Whitmer RA, Kappele LJ, Biessels GJ. An update on type 2 diabetes, vascular dementia and Alzheimer’s disease. *Exp Gerontol*. 2012;47(11):858-864. doi:10.1016/j.exger.2012.07.014

50. Ninomiya T. Epidemiological evidence of the relationship between diabetes and dementia. In: Nakabeppu Y, Ninomiya T, eds. *Diabetes Mellitus: A Risk Factor for Alzheimer’s Disease*. Advances in Experimental Medicine and Biology. Springer; 2019:13-25. doi:10.1007/978-981-13-3540-2_2

51. Perrotta M, Lembo G, Carnevale D. Hypertension and dementia: epidemiological and experimental evidence revealing a detrimental relationship. *Int J Mol Sci*. 2016;17(3):347. doi:10.3390/ijms17030347

52. Wartolowska KA, Webb AJS. Midlife blood pressure is associated with the severity of white matter hyperintensities: analysis of the UK Biobank cohort study. *Eur Heart J*. 2021;42(7):750-757. doi:10.1093/eurheartj/ehaa756

53. Cheung CY, Chan VTT, Mok VC, Chen C, Wong TY. Potential retinal biomarkers for dementia: what is new? *Curr Opin Neurol*. 2019;32(1):82-91. doi:10.1097/WCO.0000000000000645

54. Jong FJ de, Schrijvers EMC, Ikram MK, et al. Retinal vascular caliber and risk of dementia: The Rotterdam Study. *Neurology*. 2011;76(9):816-821. doi:10.1212/WNL.0b013e31820e7baa

55. Cheung CY, Tay WT, Mitchell P, et al. Quantitative and qualitative retinal microvascular characteristics and blood pressure. *J Hypertens*. 2011;29(7):1380-1391. doi:10.1097/HJH.0b013e328347266c

56. Peila R, Launer LJ. Inflammation and dementia: epidemiologic evidence. *Acta Neurol Scand Suppl*. 2006;185:102-106. doi:10.1111/j.1600-0404.2006.00693.x

57. Schmidt R, Schmidt H, Curb JD, Masaki K, White LR, Launer LJ. Early inflammation and dementia: a 25-year follow-up of the Honolulu-Asia Aging Study. *Ann Neurol*. 2002;52(2):168-174. doi:10.1002/ana.10265

58. Kinney JW, Bemiller SM, Murtishaw AS, Leisgang AM, Salazar AM, Lamb BT. Inflammation as a central mechanism in Alzheimer’s disease. *Alzheimers Dement N Y N*. 2018;4:575-590. doi:10.1016/j.trci.2018.06.014

59. Salameh Y, Bejaoui Y, El Hajj N. DNA methylation biomarkers in aging and age-related diseases. *Front Genet*. 2020;11:171. doi:10.3389/fgene.2020.00171

60. Fransquet PD, Lacaze P, Saffery R, McNeil J, Woods R, Ryan J. Blood DNA methylation as a potential biomarker of dementia: A systematic review. *Alzheimers Dement*. 2018;14(1):81-103. doi:10.1016/j.jalz.2017.10.002

61. Horvath S. DNA methylation age of human tissues and cell types. *Genome Biol*. 2013;14(10):3156. doi:10.1186/gb-2013-14-10-r115

62. Hannum G, Guinney J, Zhao L, et al. Genome-wide methylation profiles reveal quantitative views of human aging rates. *Mol Cell*. 2013;49(2):359-367. doi:10.1016/j.molcel.2012.10.016

63. Levine ME, Lu AT, Quach A, et al. An epigenetic biomarker of aging for lifespan and healthspan. *Aging*. 2018;10(4):573-591. doi:10.18632/aging.101414

64. Lu AT, Quach A, Wilson JG, et al. DNA methylation GrimAge strongly predicts lifespan and healthspan. *Aging*. 2019;11(2):303-327. doi:10.18632/aging.101684

65. Fann JR, Ribe AR, Pedersen HS, et al. Long-term risk of dementia among people with traumatic brain injury in Denmark: a population-based observational cohort study. *Lancet Psychiatry*. 2018;5(5):424-431. doi:10.1016/S2215-0366(18)30065-8

66. Nordström A, Nordström P. Traumatic brain injury and the risk of dementia diagnosis: A nationwide cohort study. *PLOS Med*. 2018;15(1):e1002496. doi:10.1371/journal.pmed.1002496

67. Loef M, Mendoza LF, Walach H. Lead (Pb) and the Risk of Alzheimer’s disease or cognitive decline: A systematic review. *Toxin Rev*. 2011;30(4):103-114. doi:10.3109/15569543.2011.624664

68. Schwartz BS, Stewart WF, Bolla KI, et al. Past adult lead exposure is associated with longitudinal decline in cognitive function. *Neurology*. 2000;55(8):1144-1150. doi:10.1212/WNL.55.8.1144

69. Reuben A. Childhood lead exposure and adult neurodegenerative disease. *J Alzheimers Dis*. 2018;64(1):17-42. doi:10.3233/JAD-180267

70. Genuis SJ, Kelln KL. Toxicant exposure and bioaccumulation: A common and potentially reversible cause of cognitive dysfunction and dementia. *Behav Neurol*. 2015;2015:e620143. doi:10.1155/2015/620143

71. Aloizou AM, Siokas V, Vogiatzi C, et al. Pesticides, cognitive functions and dementia: A review. *Toxicol Lett*. 2020;326:31-51. doi:10.1016/j.toxlet.2020.03.005

72. Silva PA, Hughes P, Williams S, Faed JM. Blood lead, intelligence, reading attainment, and behaviour in eleven year old children in Dunedin, New Zealand. *J Child Psychol Psychiatry*. 1988;29(1):43-52.

73. Reuben A, Caspi A, Belsky DW, et al. Association of childhood blood lead levels with cognitive function and socioeconomic status at age 38 years and with IQ change and socioeconomic mobility between childhood and adulthood. *JAMA*. 2017;317(12):1244-1251. doi:10.1001/jama.2017.1712

74. John P, Montgomery P. Does self-rated health predict dementia? *J Geriatr Psychiatry Neurol*. 2013;26(1):41-50. doi:10.1177/0891988713476369

75. Montlahuc C, Soumaré A, Dufouil C, et al. Self-rated health and risk of incident dementia: A community-based elderly cohort, the 3C Study. *Neurology*. 2011;77(15):1457-1464. doi:10.1212/WNL.0b013e31823303e1

76. Elliott ML, Belsky DW, Knodt AR, et al. Brain-age in midlife is associated with accelerated biological aging and cognitive decline in a longitudinal birth cohort. *Mol Psychiatry*. Published online December 10, 2019:1-10. doi:10.1038/s41380-019-0626-7

77. d’Arbeloff T, Elliott ML, Knodt AR, et al. White matter hyperintensities are common in midlife and already associated with cognitive decline. *Brain Commun*. 2019;1(1). doi:10.1093/braincomms/fcz041

78. Elliott ML, Caspi A, Houts RM, et al. Disparities in the pace of biological aging among midlife adults of the same chronological age have implications for future frailty risk and policy. *Nat Aging*. 2021;1(3):295-308. doi:10.1038/s43587-021-00044-4
