# Supplementary material for: Improving risk indexes for Alzheimer’s disease and related dementias for use in midlife
Source: Brain Commun. 2022 Oct 6;4(5):fcac223. doi: 10.1093/braincomms/fcac223 (PMC9535507; doi:10.1093/braincomms/fcac223)
Supplement: fcac223_Supplementary_Data [file fcac223_supplementary_data.zip › Manuscript_original_submission.pdf]

OXFORD UNIVERSITY PRESS | Brain Communications

Improving risk indexes for Alzheimer disease and related dementias for use in midlife

Journal:	*Brain Communications*
Manuscript ID	BRAINCOM-2022-088
Manuscript Type:	Original Article
Date Submitted by the Author:	28-Feb-2022
Complete List of Authors:	Reuben, Aaron; Duke University, Psychology and Neuroscience<br>Moffitt, Terrie; Duke University, Department of Psychology and Neuroscience; Institute of Psychiatry Psychology and Neuroscience, Social, Genetic, & Developmental Psychiatry Research Centre; Duke University School of Medicine, Department of Psychiatry & Behavioral Sciences; Duke University, Center for Genomic and Computational Biology<br>Abraham, Wickliffe; University of Otago<br>Ambler, Antony; King's College London<br>Elliott, Maxwell; Duke University<br>Hariri, Ahmad; Duke University, Department of Psychology and Neuroscience<br>Harrington, Honalee; Duke University, Psychology and Neuroscience<br>Hogan, Sean; Duke University, Psychology and Neuroscience<br>Houts, Renate; Duke University, Psychology and Neuroscience<br>Ireland, David; University of Otago, Dunedin Multidisciplinary Health and Development Research unit, Department of Psychology<br>Knodt, Annchen; Duke University, Laboratory of NeuroGenetics, Department of Psychology & Neurosicence<br>Leung, Joan; The University of Auckland<br>Pearson, Amber; Michigan State University<br>Poulton, Richie; University of Otago, Dunedin Multidisciplinary Health and Development Research Unit, Department of Psychology<br>Purdy, Suzanne; The University of Auckland, Department of Psychology<br>Ramrakha, Sandhya; University of Otago, Dunedin Multidisciplinary Health and Development Research Unit, Department of Psychology<br>Rasmussen, Line; University of Copenhagen<br>Sugden, Karen; Duke University, Psychology and Neuroscience<br>Thorne, Peter; The University of Auckland<br>Williams, Benjamin; Duke University, Psychology and Neuroscience<br>Wilson, Graham; University of Otago<br>Caspi, Avshalom; Duke University, Department of Psychology and Neuroscience; Institute of Psychiatry Psychology and Neuroscience, Social, Genetic, & Developmental Psychiatry Research Centre
Keywords:	

SCHOLARONE™
Manuscripts

Improving risk indexes for Alzheimer disease and related dementias for use in midlife

Aaron Reuben, M.E.M.,[1] Terrie E. Moffitt, Ph.D.,[1,2,3,4,5] Wickliffe C. Abraham, Ph.D.,[6]
Antony Ambler, M.Sc.,[4] Maxwell L. Elliott, M.A.,[1] Ahmad R. Hariri, Ph.D.,[1]
Honalee Harrington, B.A.,[1] Sean Hogan, M.S.W.,[7] Renate M. Houts, Ph.D.,[1]
David Ireland, Ph.D.,[7] Annchen R. Knodt, M.S.,[1] Joan Leung, Ph.D.,[8] Amber Pearson, Ph.D.,[9, 10]
Richie Poulton, Ph.D.,[7] Suzanne C. Purdy, Ph.D.,[11] Sandhya Ramrakha, Ph.D.,[7]
Line J.H. Rasmussen, Ph.D.,[12] Karen Sugden, Ph.D.,[1] Peter R. Thorne, Ph.D.,[11,13,14]
Benjamin Williams, B.Sc.,[1] Graham Wilson, Ph.D.,[7,15] and Avshalom Caspi, Ph.D.,[1,2,3,4,5]

[1]Department of Psychology and Neuroscience, Duke University, Durham, NC, USA
[2]Center for Genomic and Computational Biology, Duke University, Durham, NC, USA
[3]Department of Psychiatry and Behavioral Sciences, Duke University, Durham, NC, USA
[4]King's College London, Social, Genetic, and Developmental Psychiatry Centre,
Institute of Psychiatry, Psychology, & Neuroscience, London, UK
[5]PROMENTA, Department of Psychology, University of Oslo, Norway
[6]Brain Health Research Centre, Department of Psychology, University of Otago, Dunedin, New Zealand
[7]Dunedin Multidisciplinary Health and Development Research Unit, Department of Psychology,
University of Otago, Dunedin, New Zealand
[8]School of Psychology, the University of Auckland, Auckland, New Zealand
[9]Department of Geography, Environment, and Spatial Sciences, Michigan State University, East Lansing, MI, USA
[10]Department of Public Health, University of Otago, Wellington, New Zealand
[11]Center for Brain Research, Faculty of Medical and Health Sciences, the University of Auckland, Auckland, New Zealand
[12]Department of Clinical Research, Copenhagen University Hospital Amager and Hvidovre, Hvidovre, Denmark
[13]Department of Physiology, Faculty of Medical and Health Sciences, the University of Auckland, Auckland, New Zealand
[14]Section of Audiology, Faculty of Medical and Health Sciences, the University of Auckland, Auckland, New Zealand
[15]Department of Preventive and Social Medicine, Dunedin School of Medicine, University of Otago, Dunedin, New Zealand

For Review Only

Corresponding author: Aaron Reuben, Department of Psychology and Neuroscience, Duke University, Durham, NC, 27708. Tel: 919-684-6684. E-mail: aaron.reuben@duke.edu.

For Review Only

Abstract

Background. Knowledge of a person's risk for Alzheimer's disease and related dementias (ADRD) is required to triage candidates for preventive interventions, surveillance, and treatment trials. ADRD risk indexes exist for this purpose, but each includes only a subset of known risk factors. Information missing from published indexes could improve risk prediction.

Methods. In the Dunedin Study of a population-representative New Zealand-based birth cohort followed to midlife (N=938, 49.5% female), we compared associations of four leading risk indexes with midlife antecedents of ADRD against a novel benchmark index comprised of nearly all known ADRD risk factors (the Dunedin ADRD Risk Benchmark). Existing indexes included the CAIDE, LIBRA, ANU-ADRI, and risks selected by the Lancet Commission on Dementia. The Dunedin benchmark was comprised of 48 separate indicators of risk organized into 10 conceptually distinct risk domains. Midlife antecedents of ADRD treated as outcome measures included age-45 measures of brain structural integrity (MRI-assessed: 1. machine-learning-algorithm-estimated brain age, 2. log-transformed volume of white matter hyperintensities, and 3. mean grey matter volume of the hippocampus) and measures of brain functional integrity (1. objective cognitive function assessed via the Wechsler Adult Intelligence Scale-IV, 2. subjective problems in everyday cognitive function, and 3. objective cognitive decline measured as residualized change in cognitive scores from childhood to midlife on matched Weschler Intelligence scales).

Results. All indexes were quantitatively distributed and proved informative about midlife antecedents of ADRD, including algorithm-estimated brain age (β's from .16 to .22), white matter hyperintensities volume (β's from .16 to .19), hippocampal volume (β's from -.08 to -.11), tested cognitive deficits (β's from -.36 to -.49), everyday cognitive problems (β's from .14 to .38), and longitudinal cognitive decline (β's from -.18 to -.26). Existing indexes compared favorably to the comprehensive benchmark in their association with the brain structural integrity measures but were outperformed in their association with the functional integrity measures,

For Review Only

particularly subjective cognitive problems and tested cognitive decline. Results indicated that existing indexes could be improved with targeted additions, particularly of measures assessing socioeconomic status, physical and sensory function, epigenetic aging, and subjective overall health.

Conclusion. Existing premorbid ADRD risk indexes perform well in identifying linear gradients of risk among members of the general population at midlife, even when they include only a small subset of potential risk factors. They could be improved, however, with targeted additions to more holistically capture the different facets of risk for this multiply-determined, age-related disease.

Keywords. Alzheimer's disease. Dementia. Risk Index. Preventive medicine. Modifiable risk factors. Lifestyle. CAIDE. LIBRA. ANU-ADRI.

For Review Only

Introduction

The population-burden of Alzheimer's disease *and* related dementias (ADRD) is growing as the global population ages. Currently, 50 million people worldwide are estimated to have dementia, and that number is expected to triple within thirty years.[1] As a non-specific consequence of diverse brain pathologies,[2] dementia is multiply determined, with many potential paths leading to impairment that unfolds across years and, potentially, decades. As one consequence of the diversity of risk factors and long premorbid phase, most late-life individual ADRD interventions, including nearly all pharmaceuticals,[3] have not yet been able to prevent disease, delay progression, or dramatically improve symptoms.

Attention is now turning to preventive efforts in midlife, both behavioral and pharmacological, that can decrease premorbid ADRD risk or else delay onset of impairment to extend patients' functional years and lower the population-burden of disease.[4] Such multimodal interventions with at least some effectiveness appear to be on the horizon,[5–7] although not without controversy.[8] Promising preventive ADRD therapies will generate new challenges for dementia study and care because they are likely to be: expensive, particularly when combined with surveillance neuroimaging; scarce, at least initially; and in high-demand from consumers, both those at-risk and the legions of worried-well.[5,8] According to a recent analysis by Alzheimer's Disease International of 70,000 survey respondents from 155 countries, 95% of the general public believe that they will develop dementia at some point in their lives, and most are concerned about it.[1]

In order to target scarce intervention resources to those individuals most in-need, improve selection of participants for long-term randomized controlled trials, and support the work of clinicians who will be called upon to screen and diagnose ADRD risk, a number of premorbid

For Review Only

risk indexes have been developed for use in midlife[9]. Each index follows different risk factor selection criteria. Some, including the LIfestyle for BRAin health (LIBRA) index,[10] are comprised of only modifiable risk factors, such as physical activity and weight. Others, including the Australian National University Alzheimer's Disease Risk Index (ANU-ADRI),[11] are comprised of only risk factors that could be assessed via self-report, such as social engagement. Others still, including the Cardiovascular Risk Factors, Aging, and Dementia (CAIDE) index,[12] are comprised of only risk factors that were available in a longitudinal test cohort. Although many of the published risk indexes have shown moderate longitudinal predictive validity,[10,13–16] each is comprised of different risk factors that represent only a subset of the risks associated with ADRD – typically those that are convenient to measure in physicians' clinics (e.g., hypertension) or on questionnaires (e.g., educational attainment). Because existing risk indexes sample only a part of the universe of known risk factors for dementia, it is not clear to what extent different indexes identify the same or different individuals as "at-risk" or, further, to what extent important information may be missing from these algorithms that could improve risk prediction.

We have the good fortune to have prospective measures of nearly all putative ADRD risk factors in one population-representative birth cohort assessed repeatedly and followed to midlife: the New Zealand-based Dunedin Study. This allowed us to construct a comprehensive ADRD Risk Benchmark (hereafter DunedinARB) to evaluate the performance of existing ADRD risk indexes in terms of their associations with midlife measures of brain health that are known antecedents of ADRD. We designed the DunedinARB to include 48 separate risk indicators grouped into 10 conceptually distinct domains of risk: genetic (e.g., family history of dementia), lifestyle (e.g., tobacco and alcohol consumption), socioeconomic (e.g., low educational attainment), psychological and somatic (e.g., history of major depression), physical and sensory

For Review Only

(e.g., hearing impairment), cardio-metabolic (e.g., hypertension), inflammatory (e.g., high CRP levels), epigenetic (e.g., high scores on DNA methylation epigenetic aging clocks), harmful events (e.g., history of traumatic brain injury, TBI), and overall health (e.g., poor self-appraised subjective health). Using this comprehensive benchmark, we set out to determine whether a risk index comprised of most ADRD risk factors would be significantly more strongly associated with brain health at midlife compared to more limited indexes and, by extension, identify gaps in existing indexes. Importantly, we are not recommending this benchmark for clinical use; rather we developed it for comparative analyses here to reveal whether there is any information missing from published risk indexes that might be worth the extra effort to collect.

In addition to the DunedinARB, we generated measures of four top published ADRD risk indexes suitable for use in midlife: (1) the CAIDE; (2) the LIBRA; (3) an index comprised of risk factors selected by the Lancet Commission on Dementia Prevention, Intervention, and Care, and (4) the ANU-ADRI. (Risk scores designed primarily for determining disease risk later in life, age 65+, have been tested elsewhere.)[17–20] Dunedin Study member scores on the four published risk indexes plus the benchmark DunedinARB were then compared on their association with diverse measures of midlife brain structural and functional integrity that are known antecedents of ADRD,[21–25] including MRI-measured brain aging and white matter disease, objective tests of cognitive function and decline, and subjective reports of everyday cognitive difficulties. Analyses were conducted in two sequential stages, beginning, first, with the construction and validation of the benchmark DunedinARB and moving, second, to the construction of the four published risk indexes and a comparison of their performance to each other and to the benchmark.

For Review Only

Materials and Methods

Study Design and Population

Participants were members of the Dunedin Study. The full cohort comprises all individuals born between April 1972 and March 1973 in Dunedin, New Zealand, who were eligible based on residence in the province and who participated in the first assessment at age 3. The cohort represents the full range of socioeconomic status in the general population of New Zealand's South Island.[26] On adult health, the cohort matches the New Zealand National Health and Nutrition Survey on key indicators (e.g. body mass index, smoking, physical activity, visits to a physician)[26] and the NZ Census of citizens of the same age on educational attainment.[27] The cohort is primarily white (as self-described using fixed categories). Assessments were carried out at birth and ages 3, 5, 7, 9, 11, 13, 15, 18, 21, 26, 32, 38, and the most recent data collection was completed in April 2019, at age 45 years. Participants gave written informed consent, and Study protocols were approved by the NZ-HDEC (Health and Disability Ethics Committee).

For Review Only

Measures

We studied 48 ADRD risk indicators (organized into 10 risk domains) and six midlife measures of brain structural and functional integrity. These are described in Supplementary Tables 1 and 2, respectively.

Statistical analysis

The study followed two stages. In the first stage, the benchmark DunedinARB (Figure 1) was constructed following the criteria presented in Supplementary Table 1 and evaluated with respect to: 1) the intercorrelation of its component risk domains, 2) its overall distribution, and 3)

its sex-adjusted association, using ordinary least squares (OLS) regression, with the midlife measures of brain structural and functional integrity. The DunedinARB was generated for all Study members who attended the age 45 assessment, using Markov chain Monte Carlo (MCMC) imputation with multiple chains, in SAS, to impute missing risk indicator data. Study members alive at age 45 who did (N=938) and did not (N=59) attend the age 45 assessment were not different on measures of socioeconomic background (t=-1.592, *p*=.112) or early life cognitive function (t=-1.562, *p*=.119). Those who had died before age 45 (N=40) were not different from living study members on measures of socioeconomic background (t=-1.209, *p*=.227), although they did have lower early-life cognitive function (difference of 5.09 IQ-points in childhood, t=-2.086, *p*=.037).

In the second stage, the four published risk indexes (the CAIDE, the LIBRA, the Lancet, and the ANU-ADRI) were constructed following the criteria presented in Supplementary Table 3 and evaluated with respect to: 1) their correlation with each other and with the DunedinARB, 2) their overall distributions, and 3) their sex-adjusted association, using OLS regression, with the midlife measures of brain structural and functional integrity. The published indexes were generated for all Study members who attended the age-45 assessment (N=938).

Analyses were conducted using Stata v16.1 and SAS 9.4. Findings were checked for reproducibility by an independent data-analyst, who recreated the code based on the manuscript and applied it to a fresh dataset. This report follows the STROBE reporting guidelines for observational studies[28]. Significance tests were two-tailed, α=.05.

For Review Only

Results

Construction and validation of the benchmark DunedinARB

A comprehensive benchmark for midlife ADRD risk, the DunedinARB, was generated for each Study member who attended the age-45 assessment wave (N=938; 90.5% of the original cohort, 94.1% of the original cohort members alive at age 45; 49.5% female). The DunedinARB was comprised of nearly all known or proposed risk factors for ADRD (**Figure 1**), organized into 10 conceptually distinct domains of risk, each comprised of 2-7 distinct risk indicators (48 indicators in total). Established or proposed risk factors for ADRD were identified by non-systematic search of PubMed, Scopus, and Google Scholar for these five key words: Alzheimer's, dementia, risk, review, meta-analysis. Additional risk factors were identified by review of the 2011 NIH State of the Science Consensus Panel on ADRD risk factors[29] and review of selected factors in each of the four published risk indexes included in the second stage of our research. **Supplementary Table 1** describes the individual risk factors included in the DunedinARB, their missingness, assessment procedures, and the assignment of risk points. Total risk scores in each of the 10 risk domains were z-scored and then summed to produce the benchmark DunedinARB with equal contributions from each domain.

Figure 2, Panel A presents the overlap (correlation) among the 10 domains of risk comprising the DunedinARB. Overlap was common, such that no risk domain was entirely

For Review Only

independent of the others. However, correlations tended to be small to moderate (Pearson's r's from -.05 to .42), suggesting that each risk domain provided non-redundant information to the overall DunedinARB. The highest overlap among the conceptually distinct risk domains followed common-sense expectations, including socioeconomic status risk with lifestyle risk (r=.39, p<.001), psychological and somatic function risk with subjective overall health risk (r=.42, p<.001), and cardio-metabolic status risk with physical and sensory function risk (r=.24, p<.001). Subjective overall health risk correlated modestly with most other risk domains (r's>.25 with 7 out of 9 risk domains). **Supplementary Table 4** presents correlations among individual risk indicators within the 10 domains of risk, which varied by domain but tended to be small to moderate (r<.50).

Figure 2, Panel B presents the distribution of the overall DunedinARB in the population-representative Dunedin Study cohort (mean(SD)=0(4.86)), which followed an approximately normal, if slightly leptokurtic, distribution in the cohort (skew=0.72, kurtosis=3.39). Some Study members had very high or very low risk, but most had low to moderate risk. Overall, men had greater risk than women (mean(SD) DunedinARB score = 0.58(5.00) for males, -0.60(4.65) for females; t-test(936)=-3.74, p<.001).

To evaluate the predictive validity of the DunedinARB, we tested its association with measures of midlife brain structural and functional integrity that have been shown, in other samples, to be predictive of neurodegenerative disease in older adults[16,30–32] (described in **Supplementary Table 2**). Three MRI measures of brain structural integrity were tested: 1) the brain Age Gap Estimate (brainAGE)[33], which is the difference between an individual's chronological age at the time of imaging and their "brain age," as estimated by a machine-learning algorithm trained to predict chronological age from gray- and white-matter MRI

For Review Only

measures in independent samples ranging in age from 19 to 82;[34] 2) log-transformed volume of white matter hyperintensities,[35] a measure of ischemic and general white matter pathology; and 3) mean grey matter volume of the hippocampus,[36] a brain region central to both healthy memory function and age-related memory decline.[37] In addition to the measures of brain structural integrity, three measures of brain functional integrity were tested: 1) objective cognitive function[38] (the Wechsler Adult Intelligence Scale-IV, WAIS-IV, full-scale IQ); 2) subjective problems in everyday cognitive function,[38] such as misplacing eyeglasses, getting easily distracted, or forgetting errands, as reported on by Study members and up to three informants who knew them well; and 3) objective cognitive decline[38] (measured as residualized change in full-scale IQ scores from childhood to age 45 years, assessed via matched Weschler Intelligence scales).

Across all six measures, the DunedinARB score proved informative about midlife brain health (**Figure 2, Panel C**). First, Study members with higher DunedinARB scores demonstrated lower structural brain integrity: each standard deviation increase in the DunedinARB was associated with an additional 1.77-years older algorithm-estimated brainAGE (95%CI: 1.25, 2.29, p-value<.001), a 0.16-log mm^3 greater volume of white matter hyperintensities (95%CI: 0.10, 0.21), p-value<.001), and a 79.04 mm^3 smaller hippocampal grey matter volume (95%CI: -129.82, -28.27, p-value=.002).

Second, Study members with higher DunedinARB scores demonstrated lower cognitive function: each standard deviation increase in the DunedinARB was associated with an additional 7.39-point lower score in full-scale IQ (95%CI: -8.25, -6.54, p-value<.001) and a 0.29-standard deviation higher score on self and informant-reported scales assessing everyday cognitive problems (95%CI: 0.24, 0.34, p-value<.001).

For Review Only

Third, Study members with higher DunedinARB scores demonstrated greater longitudinal decline in cognitive function from childhood to adulthood, a hallmark antecedent of ADRD[39]: each standard deviation increase in the DunedinARB was associated with an additional 2.51-point decline in full-scale IQ score from childhood to midlife (95%CI: -3.11, -1.90, *p*-value<.001).

Supplementary Table 5 presents the association of the 10 domains of risk comprising the DunedinARB (Figure 1) with the midlife outcome measures.

For Review Only

Construction of published risk indexes and comparison to the benchmark DunedinARB

Scores for four published pre-morbid ADRD risk indexes that have received considerable research attention to date were generated for each Dunedin Study member who attended the age-45 assessment wave (N=938). For all risk factors included in each index, Study members were assigned risk points following the index's published guidelines (**Supplementary Table 3**). The four indexes were:

1. The Cardiovascular Risk Factors, Aging, and Incidence of Dementia risk index (CAIDE, model-2; 8 indicators, including genetic risk),[12,14,15]
2. The LIfestyle for BRAin health risk index (LIBRA; 12 indicators),[10,40,41]
3. The Lancet Commission on Dementia Prevention, Intervention, and Care risk factor list (Lancet; 12 indicators),[42] and
4. The Australian National University Alzheimer's Disease Risk Index (ANU-ADRI; 15 indicators).[11,43]

What is the population distribution of risk as measured by published ADRD risk indexes?

Table 1 presents the risk factors that comprise each of the four published ADRD risk indexes. The CAIDE includes 8 risk indicators, the LIBRA 12, the Lancet 12, and the ANU-ADRI 15. Altogether the four indexes utilize a total of 20 unique indicators (as compared to the 48 included in the benchmark DunedinARB). Consequently a large number of proposed risk factors with consistent empirical support of predicting dementia were not included in any index (e.g., poor sleep,[44,45] poor sense of smell,[46–48] disadvantageous personality traits,[49] etc.).

Despite their inclusion of different, conceptually distinct risk factors, there was moderate to high correlation among the risk indexes (Pearson's r's between .55 and .80, p-values <.001) (**Table 2**), indicating that they largely ranked Study members' risk similarly, although rankings were not interchangeable. The distribution of risk scores was similar across the four indexes and DunedinARB **(Table 2**).

Table 3 presents the overlap (correlation) of the four published indexes with the 10 domains of risk (e.g., genetic, lifestyle, etc.) captured by the DunedinARB's 48 indicators. Cells highlighted in yellow indicate risk domains specifically represented in the construction of each index, documenting that each risk index, by design, was missing some risk information. The table reveals two points of interest. First, despite missing some risk information, each risk index captured information about risk domains that were not directly assessed in that index. For example, high LIBRA scores reflect socioeconomic risk as much as do high CAIDE, LANCET, and ANU-ADRI scores, although socioeconomic risk is not included in the LIBRA index but is included in the three other indexes. Second, some risk information was notably absent from some risk indexes. For example, genetic risk is not reflected in high LIBRA, Lancet, or ANU-ADRI scores; CAIDE and LIBRA only weakly capture risks associated with harmful events and

For Review Only

exposures (e.g., TBI, neurotoxicant exposures, etc.); and CAIDE does not capture psychological and somatic risk.

Are published risk indexes informative about midlife brain health?

Table 4 presents the association of the four published risk indexes with the midlife measures of brain structural and functional integrity that are antecedents of ADRD. Despite being comprised of risk factors for dementia in old age, all four risk indexes were significantly associated with most measures of midlife brain health, approximately 20-40 years before dementia diagnoses are expected.

First, Study members with higher ADRD risk scores had lower structural brain integrity, including older algorithm-estimated brainAGE (sex-adjusted β's between .16 and .22, *p*-values <.001), greater volume of white matter hyperintensities (sex-adjusted β's between .13 and .16, *p*-values <.001), and smaller hippocampal gray matter volume (sex-adjusted β's between -.08 and -.11, *p*-values <.01).

Second, Study members with higher ADRD risk scores demonstrated lower midlife cognitive function, including lower full-scale IQ scores (sex-adjusted β's between -.36 and -.44, *p*-values <.001) and higher scores on scales assessing everyday cognitive problems (sex-adjusted β's between .14 and .32, *p*-values <.001).

Third, Study members with higher ADRD risk scores demonstrated greater longitudinal decline in cognitive function from childhood to adulthood, a hallmark premorbid feature of ADRD (sex-adjusted β's between -.15 and -.20, *p*-values <.001).

For Review Only

Do published risk indexes represent an efficient mix of putative risk factors for ADRD?

In terms of association with the midlife measures of brain structural and functional integrity that are antecedents of ADRD, all four published risk indexes compared favorably to the benchmark DunedinARB (**Table 4**), and no one risk index notably outperformed all others across all outcomes. Published risk indexes thus likely represent an efficient mix of risk factors for ADRD, at least at midlife, despite only including a small portion of putative risks. The inclusion of more risk factors, as in the DunedinARB, tended to broadly increase associations with the outcome measures, but only modestly, and not always significantly.

On the MRI measures of brain structural integrity, the DunedinARB tended to outperform the four published indexes, but the improvements were small (difference in β's <.06). Tests of differences between dependent correlations[50] revealed that the DunedinARB significantly outperformed the CAIDE in its association with brainAGE (DunedinARB sex-adjusted β=.22, 95%CI: .16, .29), and outperformed the LIBRA, Lancet, and ANU-ADRI in its association with white matter hyperintensity volume (DunedinARB sex-adjusted β=.19, 95%CI: .13, .26). There were no significant differences among published index or benchmark associations with hippocampal volume.

For Review Only

On the cognitive measures, the DunedinARB always outperformed the four published indexes, as indicated by tests of dependent correlations,[50] but improvements varied by cognitive outcome. Differences in β's between the DunedinARB and the published indexes ranged from .05 to .13 for objective midlife IQ and .06 to .24 for subjective cognitive problems.

On the measure of longitudinal cognitive decline, the DunedinARB again consistently outperformed all of the published indexes (sex-adjusted β=-.26, 95%CI: -.32, -.20), as indicated by tests of dependent correlations (differences in β's ranged from .06 to 0.11).

These findings indicated that information contained in the DunedinARB and missing from the published indexes was uniquely informative about cognitive ability and, notably, longitudinal cognitive decline unfolding across adulthood. To evaluate this hypothesis further, we performed a number of post hoc sensitivity tests. First, models regressing the residualized cognitive decline measure on the DunedinARB were re-estimated including each of the four published risk indexes as covariates, in turn, to determine whether the DunedinARB was a significant predictor of cognitive decline over and above each published risk index (4 new tests in total), and it was. The DunedinARB remained significantly associated with cognitive decline in all models including the 4 risk indexes. Second, to determine whether a particular risk domain

For Review Only

within the DunedinARB was a significant predictor of cognitive decline over and above each published risk index, models regressing the residualized cognitive decline measure on the 10 risk domains comprising the DunedinARB were estimated including each of the four published risk indexes in turn (40 new tests in total). Results of these tests are reported in **Supplementary Table 6**. Four domains of risk were significantly associated with cognitive decline over and above all published indexes (socioeconomic status risk, physical and sensory function risk, epigenetic aging risk, and subjective overall health risk).

For Review Only

Discussion

We compared four published ADRD risk indexes to a comprehensive benchmark comprising 48 indicators covering 10 domains of risk, and mapped all five ADRD-risk scores onto midlife measures of brain structural and functional integrity that are antecedents of ADRD. This evaluation generated seven findings.

First, despite consistent overlap among distinct domains of ADRD risk (e.g., individuals with greater lifestyle-based risks such as tobacco smoking also had greater epigenetic risks, such as advanced cellular aging), all 10 domains of ADRD risk were found to be non-redundant; each added some unique information to a comprehensive benchmark. Second, midlife risk for later-life ADRD followed an approximately normal distribution on all ADRD-risk scores in our population-representative cohort. While ADRD diagnosis is categorical, relative risk appears to be quantitatively distributed, with the majority of the cohort, in midlife, at low-to-moderate risk

of late-life ADRD. This quantitative risk distribution poses a challenge to decision-making about premorbid dementia surveillance and therapeutics, given that cut-offs are not yet known.

Third, dementia risk indexes were informative about brain structural and functional integrity decades before the expected emergence of clinical diagnosis. Study members with higher risk scores demonstrated older brains (i.e., older algorithm-estimated brain age from MRI data), more white matter pathology, smaller hippocampi (a brain region critical for memory function and a primary locus of neurodegeneration in ADRD), relative deficits in tested cognitive performance, elevated subjective problems in everyday cognitive function, and longitudinal cognitive decline across adulthood, all at the relatively young age of 45. These findings reinforce the emerging perspective, voiced by the Lancet Commission on Dementia Prevention, Intervention, and Care, that it is "never too early" to prevent dementia.[42(p1)]

Fourth, published risk indexes comprised of small subsets of putative ADRD risk factors were correlated; they tended to rank the same individuals as at higher or lower-risk for ADRD as each other, despite each including a different collection of risk factors. All published indexes (the CAIDE, the LIBRA, the Lancet, and the ANU-ADRI) were found to contain information about domains of risk that they did not directly assess, including risk related to systemic inflammation, epigenetic aging, and subjective overall health. Individual indexes were, however,

For Review Only

found to selectively lack information about particular domains of risk (Table 3), notably genetic risk, harmful events and exposures (e.g., history of TBI), and psycho-somatic risks (e.g., history of depression), depending on the particular index.

Fifth, published risk indexes were determined to represent an efficient mix of ADRD risk factors, as they tended to perform nearly as well as the more comprehensive benchmark DunedinARB in their association with the outcome-measure antecedents of ADRD, particularly those of brain structural integrity. The DunedinARB outperformed the published indexes in its associations with these structural measures, but only modestly, and not always significantly. For example, the shortest index, the CAIDE, demonstrated a similar (although weaker) pattern of findings to the comprehensive DunedinARB, despite being comprised of 80% fewer indicators. This suggests that some conceptually distinct risk factors supported by the current literature are best considered proxy measures of other risk factors (and not unique contributors to risk). Other risk factors may only influence brain health after midlife. This latter potential would reinforce the Lancet Commission's life-course model of dementia prevention,[42] which argues that the influence of ADRD risk factors can vary across the life-course.

Sixth, while published ADRD risk indexes were found to perform well against the DunedinARB on many outcome measures, post hoc sensitivity tests determined that the DunedinARB was informative over and above each of the published indexes about cognitive

For Review Only

decline unfolding across adulthood. This additional predictive value could be accounted for, in part, by the DunedinARB's inclusion of risk domains that have largely been overlooked by existing indexes, including socioeconomic risk, physical and sensory function risk, epigenetic risk, and subjective overall health risk (Supplementary Table 6). This suggests that rounding out the four published indexes by adding any of these selectively missing risk domains may improve their ability to select individuals at-risk for ADRD. This could be done in a manner that maintains the unique benefits of each particular index (e.g., using self-report measures for the ANU-ADRI, making use of information readily available to physicians for the CAIDE, etc.).

Seventh, a close look at subjective cognitive complaints, here measured as reports by Study members and informants about everyday cognitive problems, such as misplacing eyeglasses, getting easily distracted, or forgetting errands, is informative because subjective complaints typically bring patients to clinical attention, lead them to volunteer for intervention trials, or prompt them to request medications. 93% of this midlife cohort had one or more subjective complaints and 75% had three or more. Comparison of the published indexes with the comprehensive DunedinARB suggested that existing indexes could do a better job of capturing risks related to developing cognitive complaints. Research in larger samples can inform as to

For Review Only

which index best discriminates conversion to ADRD among the pool of individuals presenting to clinicians with subjective cognitive complaints.

Limitations

This study has several limitations. First, it was observational and cannot establish causation. Reverse causation is possible; for example, cognitive decline may have promoted socioeconomic-status risk. However, for purposes of risk prediction, causation is not a central consideration. Second, we investigated only one cohort in one country and findings should be replicated elsewhere. Third, brain-health measures of ADRD antecedents were largely, although not exclusively, cross-sectional midlife measures. Continued follow-up will be required to more precisely estimate ADRD risk (for example, using plasma biomarkers) as well as to document risk factor associations with further longitudinal declines in brain integrity. Fourth, while the DunedinARB included most known or suspected measures of ADRD risk, some measures were unavailable, including blood, CSF or PET ADRD biomarkers, or exposure to outdoor air pollution, which could be added for further investigation.

For Review Only

Conclusion

With the coming advent of successful yet costly premorbid ADRD interventions on the horizon, the time has arrived to refine screening systems to differentiate the truly high-risk in urgent need of preventive treatment from the moderate-risk majority and legions of worried-well, for whom benefits may not outweigh side-effects. With a dizzying array of factors that may

elevate dementia risk differentially across the lifespan, it can be difficult to know where to focus clinical and research attention. Our study findings suggest that risk indexes are informative about risk as early as midlife, with differences in brain integrity already apparent by age 45. Findings also suggest that published ADRD risk inventories, including the CAIDE, the LIBRA, the Lancet, and the ANU-ADRI, perform well in identifying linear gradients of risk among members of the general population, even when they include only a small subset of potential risk factors. Our findings also suggest, however, that these published risk indexes could be improved with targeted additions to more holistically capture the different facets of risk that contribute to these costly and difficult-to-treat diseases of late-life.

For Review Only

Acknowledgments

We thank members of the Advisory Board for the Dunedin Neuroimaging Study, Dunedin Study members, Unit research staff, Pacific Radiology Group staff, and Study founder Phil Silva, Ph.D. We would like to acknowledge the assistance of the Duke Molecular Physiology Institute Molecular Genomics Core for the generation of data for the manuscript.

Data Availability

The Dunedin Study datasets reported in the current article are available on request by qualified scientists. Requests require a concept paper describing the purpose of data access, ethical approval at the applicant's university and provision for secure data access (https://moffittcaspi.trinity.duke.edu/research-topics/dunedin). We offer secure access on the Duke, Otago, and King's College London campuses.

Funding

This research was supported by the National Institute on Aging (NIA) grants R01AG032282, R01AG069939, and R01AG049789, and the UK Medical Research Council grant MR/P005918/1. Additional support was provided by the Jacobs Foundation, NIA P30 AG028716, and NIA P30 AG034424. The Dunedin Multidisciplinary Health and Development Research Unit was supported by the New Zealand Health Research Council (Project Grants 15-265 and 16-604) and the New Zealand Ministry of Business, Innovation, and Employment (MBIE). This work used a high-performance computing facility partially supported by grant 2016-IDG-1013 (HARDAC+: Reproducible HPC for Next-generation Genomics") from the North Carolina Biotechnology Center. Neuroimaging was supported by Brain Research New Zealand. A.R. was supported by the US-National Institute of Environmental Health Sciences grant F31ES029358.

Competing Interests

The authors declare no competing interests.

For Review Only

For Review Only

References

1. International AD. World Alzheimer Report 2019: Attitudes to dementia. Published online September 20, 2019. Accessed February 16, 2021. https://www.alzint.org/resource/world-alzheimer-report-2019/

2. Schneider JA, Arvanitakis Z, Bang W, Bennett DA. Mixed brain pathologies account for most dementia cases in community-dwelling older persons. *Neurology*. 2007;69(24):2197-2204. doi:10.1212/01.wnl.0000271090.28148.24

3. Mehta D, Jackson R, Paul G, Shi J, Sabbagh M. Why do trials for Alzheimer's disease drugs keep failing? A discontinued drug perspective for 2010–2015. *Expert Opin Investig Drugs*. 2017;26(6):735-739. doi:10.1080/13543784.2017.1323868

4. National Academies of Sciences, Engineering, and Medicine, Health and Medicine Division, Board on Health Sciences Policy, Committee on Preventing Dementia and Cognitive Impairment. *Preventing Cognitive Decline and Dementia: A Way Forward*. (Downey A, Stroud C, Landis S, Leshner AI, eds.). National Academies Press (US); 2017. Accessed February 16, 2021. http://www.ncbi.nlm.nih.gov/books/NBK436397/

5. Musiek ES, Morris JC. Possible consequences of the approval of a disease-modifying therapy for Alzheimer disease. *JAMA Neurol*. 2021;78(2):141-142. doi:10.1001/jamaneurol.2020.4478

6. Ngandu T, Lehtisalo J, Solomon A, et al. A 2 year multidomain intervention of diet, exercise, cognitive training, and vascular risk monitoring versus control to prevent cognitive decline in at-risk elderly people (FINGER): a randomised controlled trial. *Lancet Lond Engl*. 2015;385(9984):2255-2263. doi:10.1016/S0140-6736(15)60461-5

7. Moll van Charante EP, Richard E, Eurelings LS, et al. Effectiveness of a 6-year multidomain vascular care intervention to prevent dementia (preDIVA): a cluster-randomised controlled trial. *Lancet Lond Engl*. 2016;388(10046):797-805. doi:10.1016/S0140-6736(16)30950-3

8. Powell T. What a bad day science had. The Hastings Center. Published June 8, 2021. Accessed June 18, 2021. https://www.thehastingscenter.org/what-a-bad-day-science-had/

9. Stephen R, Soininen H. Biomarker validation of a dementia risk prediction score. *Nat Rev Neurol*. 2020;16(3):135-136. doi:10.1038/s41582-020-0316-8

10. Vos SJB, van Boxtel MPJ, Schiepers OJG, et al. Modifiable risk Factors for prevention of dementia in midlife, late life and the oldest-old: Validation of the LIBRA index. *J Alzheimers Dis*. 2017;58(2):537-547. doi:10.3233/JAD-161208

11. Anstey KJ, Cherbuin N, Herath PM. Development of a new method for assessing global risk of Alzheimer's disease for use in population health approaches to prevention. *Prev Sci*. 2013;14(4):411-421. doi:10.1007/s11121-012-0313-2

For Review Only

12. Kivipelto M, Ngandu T, Laatikainen T, Winblad B, Soininen H, Tuomilehto J. Risk score for the prediction of dementia risk in 20 years among middle aged people: a longitudinal, population-based study. *Lancet Neurol*. 2006;5(9):735-741. doi:10.1016/S1474-4422(06)70537-3

13. Kaffashian S, Dugravot A, Elbaz A, et al. Predicting cognitive decline. *Neurology*. 2013;80(14):1300-1306. doi:10.1212/WNL.0b013e31828ab370

14. Vuorinen M, Spulber G, Damangir S, et al. Midlife CAIDE dementia risk score and dementia-related brain changes up to 30 years later on Magnetic Resonance imaging. *J Alzheimers Dis*. 2015;44(1):93-101. doi:10.3233/JAD-140924

15. Exalto LG, Quesenberry CP, Barnes D, Kivipelto M, Biessels GJ, Whitmer RA. Midlife risk score for the prediction of dementia four decades later. *Alzheimers Dement*. 2014;10(5):562-570. doi:10.1016/j.jalz.2013.05.1772

16. O'Brien JT, Firbank MJ, Ritchie K, et al. Association between midlife dementia risk factors and longitudinal brain atrophy: the PREVENT-Dementia study. *J Neurol Neurosurg Psychiatry*. 2020;91(2):158-161. doi:10.1136/jnnp-2019-321652

17. Tang EYH, Harrison SL, Errington L, et al. Current developments in dementia risk prediction modelling: An updated systematic review. *PLoS ONE*. 2015;10(9). doi:10.1371/journal.pone.0136181

18. Licher S, Yilmaz P, Leening MJG, et al. External validation of four dementia prediction models for use in the general community-dwelling population: a comparative analysis from the Rotterdam Study. *Eur J Epidemiol*. 2018;33(7):645-655. doi:10.1007/s10654-018-0403-y

19. Bello-Chavolla OY, Aguilar-Salinas CA, Avila-Funes JA. The type 2 diabetes-specific dementia risk score (DSDRS) is associated with frailty, cognitive and functional status amongst Mexican community-dwelling older adults. *BMC Geriatr*. 2020;20(1):363. doi:10.1186/s12877-020-01776-5

20. Barnes DE, Covinsky KE, Whitmer RA, Kuller LH, Lopez OL, Yaffe K. Dementia risk indexes: A framework for identifying individuals with a high dementia risk. *Alzheimers Dement J Alzheimers Assoc*. 2010;6(2):138-141. doi:10.1016/j.jalz.2010.01.005

21. Wilson RS, Leurgans SE, Boyle PA, Bennett DA. Cognitive decline in prodromal Alzheimer disease and mild cognitive impairment. *Arch Neurol*. 2011;68(3):351-356. doi:10.1001/archneurol.2011.31

22. Amieva H, Jacqmin-Gadda H, Orgogozo JM, et al. The 9 year cognitive decline before dementia of the Alzheimer type: a prospective population-based study. *Brain*. 2005;128(5):1093-1101. doi:10.1093/brain/awh451

For Review Only

23. Elias MF, Beiser A, Wolf PA, Au R, White RF, D'Agostino RB. The preclinical phase of alzheimer disease: A 22-year prospective study of the Framingham Cohort. *Arch Neurol*. 2000;57(6):808-813.

24. Cole JH, Franke K. Predicting age using neuroimaging: Innovative brain ageing biomarkers. *Trends Neurosci*. 2017;40(12):681-690. doi:10.1016/j.tins.2017.10.001

25. den Heijer T, van der Lijn F, Koudstaal PJ, et al. A 10-year follow-up of hippocampal volume on magnetic resonance imaging in early dementia and cognitive decline. *Brain*. 2010;133(4):1163-1172. doi:10.1093/brain/awq048

26. Poulton R, Moffitt TE, Silva PA. The Dunedin Multidisciplinary Health and Development Study: overview of the first 40 years, with an eye to the future. *Soc Psychiatry Psychiatr Epidemiol*. 2015;50(5):679-693. doi:10.1007/s00127-015-1048-8

27. Richmond-Rakerd LS, D'Souza S, Andersen SH, et al. Clustering of health, crime and social-welfare inequality in 4 million citizens from two nations. *Nat Hum Behav*. Published online January 20, 2020:1-10. doi:10.1038/s41562-019-0810-4

28. von Elm E, Altman DG, Egger M, et al. The Strengthening the Reporting of Observational Studies in Epidemiology (STROBE) statement: guidelines for reporting observational studies. *Lancet Lond Engl*. 2007;370(9596):1453-1457. doi:10.1016/S0140-6736(07)61602-X

29. Daviglus ML, Plassman BL, Pirzada A, et al. Risk factors and preventive interventions for Alzheimer disease: State of the science. *Arch Neurol*. 2011;68(9):1185-1190. doi:10.1001/archneurol.2011.100

30. Rensma SP, van Sloten TT, Launer LJ, Stehouwer CDA. Cerebral small vessel disease and risk of incident stroke, dementia and depression, and all-cause mortality: A systematic review and meta-analysis. *Neurosci Biobehav Rev*. 2018;90(October 2017):164-173. doi:10.1016/j.neubiorev.2018.04.003

31. Franke K, Gaser C. Longitudinal changes in individual BrainAGE in healthy aging, mild cognitive impairment, and Alzheimer's disease. *GeroPsych J Gerontopsychology Geriatr Psychiatry*. 2012;25(4):235-245. doi:10.1024/1662-9647/a000074

32. Fjell AM, Westlye LT, Grydeland H, et al. Accelerating cortical thinning: Unique to dementia or universal in aging? *Cereb Cortex*. 2014;24(4):919-934. doi:10.1093/cercor/bhs379

33. Elliott ML, Belsky DW, Knodt AR, et al. Brain-age in midlife is associated with accelerated biological aging and cognitive decline in a longitudinal birth cohort. *Mol Psychiatry*. Published online December 10, 2019:1-10. doi:10.1038/s41380-019-0626-7

34. Liem F, Varoquaux G, Kynast J, et al. Predicting brain-age from multimodal imaging data captures cognitive impairment. *NeuroImage*. 2017;148(November 2016):179-188. doi:10.1016/j.neuroimage.2016.11.005

For Review Only

35. d'Arbeloff T, Elliott ML, Knodt AR, et al. White matter hyperintensities are common in midlife and already associated with cognitive decline. *Brain Commun*. 2019;1(1). doi:10.1093/braincomms/fcz041

36. Elliott ML, Caspi A, Houts RM, et al. Disparities in the pace of biological aging among midlife adults of the same chronological age have implications for future frailty risk and policy. *Nat Aging*. 2021;1(3):295-308. doi:10.1038/s43587-021-00044-4

37. Nyberg L, Pudas S. Successful Memory Aging. *Annu Rev Psychol*. Published online 2019. doi:10.1146/annurev-psych-010418-103052

38. Reuben A, Elliott ML, Abraham WC, et al. Association of childhood lead exposure with MRI measurements of structural brain integrity in midlife. *JAMA*. 2020;324(19):1970-1979. doi:10.1001/jama.2020.19998

39. Marioni RE, Proust-Lima C, Amieva H, et al. Social activity, cognitive decline and dementia risk: a 20-year prospective cohort study. *BMC Public Health*. 2015;15(1):1089. doi:10.1186/s12889-015-2426-6

40. Schiepers OJG, Köhler S, Deckers K, et al. Lifestyle for Brain Health (LIBRA): a new model for dementia prevention. *Int J Geriatr Psychiatry*. 2018;33(1):167-175. doi:10.1002/gps.4700

41. Deckers K, van Boxtel MPJ, Schiepers OJG, et al. Target risk factors for dementia prevention: a systematic review and Delphi consensus study on the evidence from observational studies. *Int J Geriatr Psychiatry*. 2015;30(3):234-246. doi:10.1002/gps.4245

42. Livingston G, Huntley J, Sommerlad A, et al. Dementia prevention, intervention, and care: 2020 report of the Lancet Commission. *The Lancet*. 2020;396(10248):413-446. doi:10.1016/S0140-6736(20)30367-6

43. Anstey KJ, Cherbuin N, Herath PM, et al. A self-report risk index to predict occurrence of dementia in three independent cohorts of older adults: The ANU-ADRI. *PLOS ONE*. 2014;9(1):e86141. doi:10.1371/journal.pone.0086141

44. Spira AP, Chen-Edinboro LP, Wu MN, Yaffe K. Impact of sleep on the risk of cognitive decline and dementia. *Curr Opin Psychiatry*. 2014;27(6):478-483. doi:10.1097/YCO.0000000000000106

45. Wennberg AMV, Wu MN, Rosenberg PB, Spira AP. Sleep disturbance, cognitive decline, and dementia: A review. *Semin Neurol*. 2017;37(4):395-406. doi:10.1055/s-0037-1604351

46. Ding D, Xiao Z, Liang X, Wu W, Zhao Q, Cao Y. Predictive value of odor identification for incident dementia: The Shanghai Aging Study. *Front Aging Neurosci*. 2020;12:266. doi:10.3389/fnagi.2020.00266

For Review Only

47. Roberts RO, Christianson TJH, Kremers WK, et al. Association between olfactory dysfunction and amnestic Mild Cognitive Impairment and Alzheimer Disease Dementia. *JAMA Neurol*. 2016;73(1):93-101. doi:10.1001/jamaneurol.2015.2952

48. Devanand DP, Lee S, Manly J, et al. Olfactory deficits predict cognitive decline and Alzheimer dementia in an urban community. *Neurology*. 2015;84(2):182-189. doi:10.1212/WNL.0000000000001132

49. Low LF, Harrison F, Lackersteen SM. Does personality affect risk for dementia? A systematic review and meta-analysis. *Am J Geriatr Psychiatry*. 2013;21(8):713-728. doi:10.1016/j.jagp.2012.08.004

50. Steiger JH. Tests for comparing elements of a correlation matrix. *Psychol Bull*. 1980;87(2):245-251. doi:10.1037/0033-2909.87.2.245

For Review Only

Tables and Figures

Figure 1. Schematic of the Dunedin Alzheimer's Disease and Related Dementias Risk Benchmark (DunedinARB).

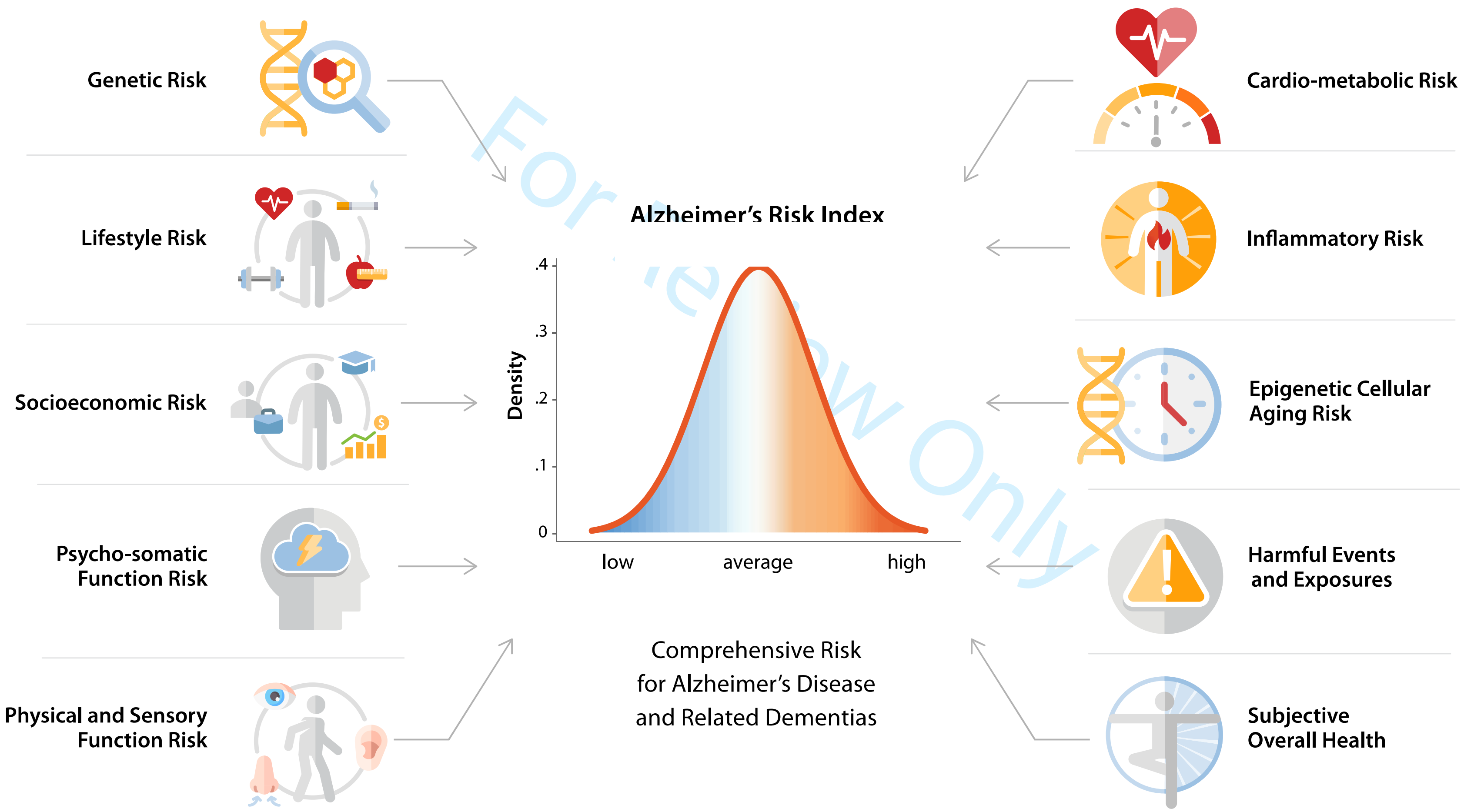

For Review Only

Note. The comprehensive Dunedin Alzheimer's Disease and Related Dementias Risk Benchmark (DunedinARB) is comprised of 48 risk indicators grouped into 10 conceptually distinct domains. Genetic risk includes family history of dementia and APOE ε4 allele status. Lifestyle risk includes physical activity, diet, tobacco smoking, alcohol consumption, folic acid supplementation, and regular prophylactic NSAID use. Socioeconomic risk includes occupational and educational attainment. Psycho-somatic Function risk includes chronic pain, history of migraine, history of depression, social isolation, sleep quality, neuroticism, and conscientiousness. Physical and Sensory Function risk includes balance, gait, hearing acuity and subjective hearing function, objective and subjective vision function, and sense of smell. Cardio-Metabolic Status risk includes hypertension, obesity, and diabetes status, total cholesterol, triglycerides, and retinal vascular health. Inflammatory risk includes CRP, IL-6, and suPAR levels and history of rheumatoid arthritis. Epigenetic Cellular Aging risk includes four separate DNA methylation "aging" clocks (Horvath, Hannum, PhenoAge, and GrimAge). Harmful Events and Exposures risk includes childhood lead exposure, occupational exposure to neurotoxicants, and history of traumatic brain injury. Subjective Overall Health risk includes self, informant, and research-worker ratings of Study member overall health. Details on the individual risk factors and indicators are provided in Supplementary Table 1.

For Review Only

Figure 2. The Dunedin Alzheimer's Disease and Related Dementias Risk Benchmark (DunedinARB) performance characteristics.

Panel A. Overlap (correlation, Pearson's r) among the 10 domains of risk comprising the DunedinARB.

	Genetic	Lifestyle	Socio-economic	Psycho-somatic	Physio-sensory	Cardio-metabolic	Inflam-matory	Epigen-etic	Harmful Events
1. Genetic	---								
2. Lifestyle	.04	---							
3. Socioeconomic	.03	.39***	---						
4. Psycho-somatic	-.05	.13***	.20***	---					
5. Physio-sensory	.06	.17***	.29***	.25***	---				
6. Cardio-metabolic	.06	.10**	.13***	.00	.24***	---			
7. Inflammatory	-.04	.12***	.16***	.22***	.21***	.13***	---		
8. Epigenetic	.04	.26***	.19***	.08*	.16***	.11**	.18***	---	
9. Harmful events	.04	.08*	.13***	.07*	.14***	.09**	.00	.01	---
10. Overall health	-.03	.33***	.38***	.42***	.38***	.26***	.36***	.25***	.11***

Note. *p-values <.05, **p-values <.01, ***p-values <.001. Shading reflects association size, with darker colors highlighting larger associations. Risk domains are as follows: 1. Genetic risk; 2. Lifestyle risk, 3. Socioeconomic risk; 4. Psycho-Somatic Function risk; 5. Physical and Sensory Function risk; 6. Cardio-Metabolic Status risk; 7. Inflammatory risk; 8. Epigenetic Cellular Aging risk; 9. Harmful Events and Exposures risk; 10.

For Review Only

Subjective Overall Health risk. Individual risk indicators contributing to each of the 10 risk domains are detailed in Supplementary Table 1; risk domains included between 2 and 7 indicators each.

For Review Only

Panel B. Distribution of DunedinARB scores in the cohort

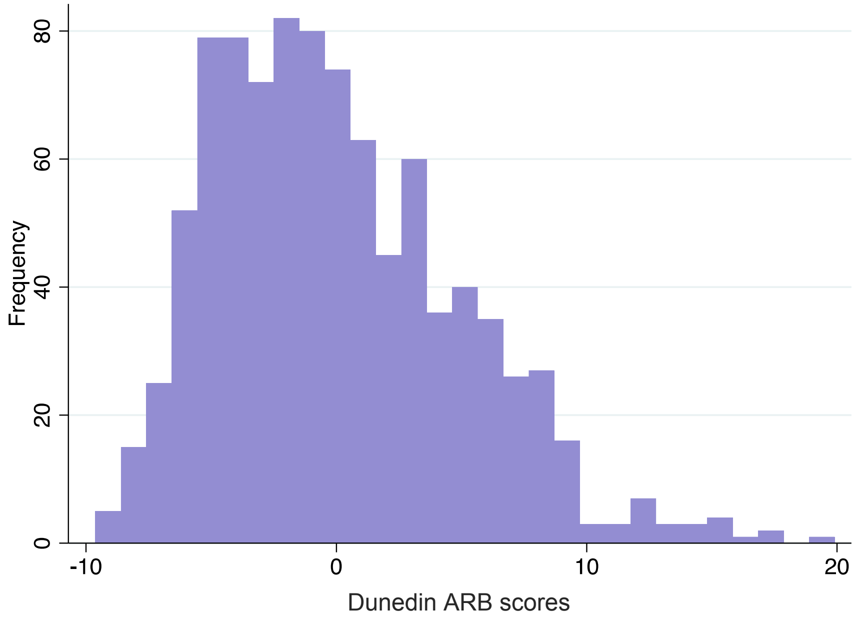

Panel C. Association of the DunedinARB with midlife measures of brain structural (MRI) and functional (cognitive) integrity.

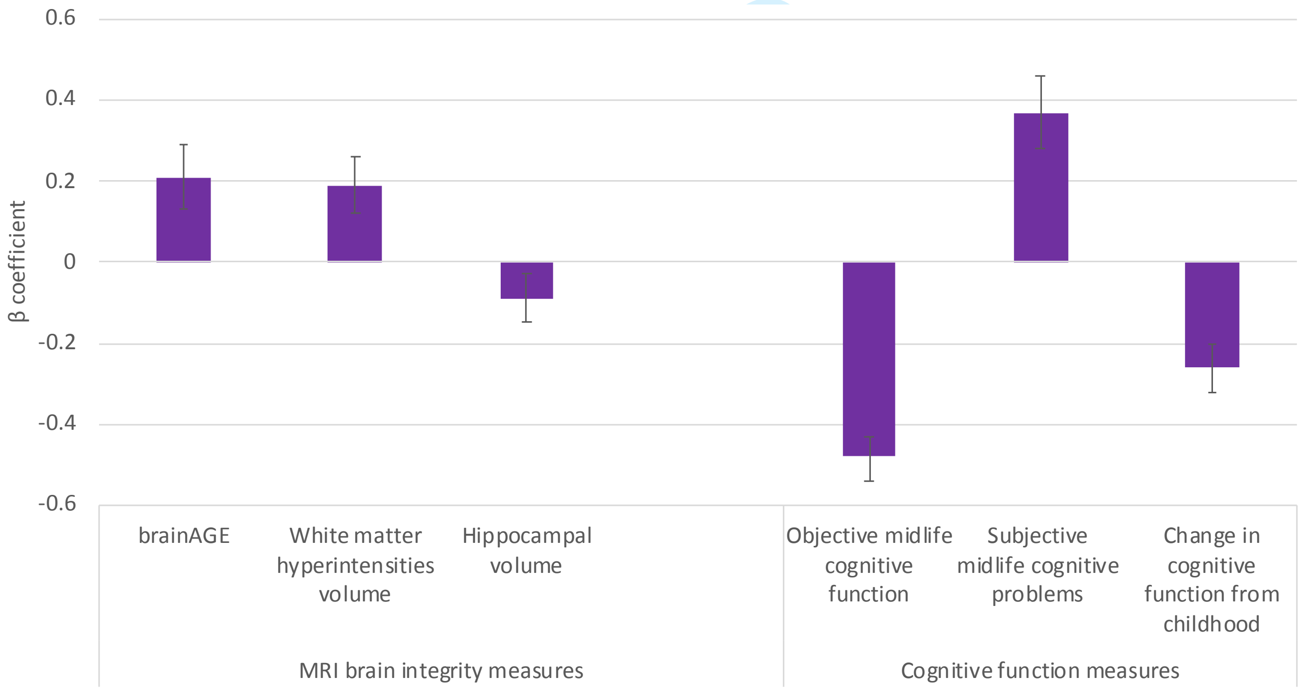

Table 1. Composition of the 4 published ADRD risk indexes.

		Risk Index			
**Risk Domain**	**Risk Factor**	**CAIDE**	**LIBRA**	**LANCET**	**ANU-ADRI**
1. Genetic risk	APOE status	■			
2. Lifestyle risk	Physical activity	■	■	■	■
	Alcohol consumption		■	■	■
	Tobacco smoking		■	■	■
	Diet		■		■
3. Socioeconomic status risk	Education	■		■	■
4. Psycho-somatic risk	Depression		■	■	■
	Cognitive engagement		■		■
	Social engagement / isolation			■	■
5. Physical / sensory function risk	Hearing impairment			■	
6. Cardio-metabolic risk	Body Mass Index (obesity)	■	■	■	■
	Hypertension	■	■	■	
	High cholesterol	■	■		■
	Diabetes		■	■	■
	Coronary heart disease		■		
	Chronic kidney disease		■		
7. Inflammatory risk					
8. DNA methylation aging risk					

For Review Only

9. Harmful events and exposures	Traumatic brain injury				
	Air pollution exposure				
	Pesticide exposure				
10. Subjective overall health risk					
Additional demographic risk factors	Age				
	Sex				
	**Total No. Factors**	8	12	12	15

Note. The DunedinARB includes direct or proxy measures of all risks on this table with the exception of air pollution exposure and cognitive engagement. Age and sex did not contribute to the DunedinARB score as the cohort is of equal age and sex was retained for use in analyses as a covariate to account for known sex-differences in brain structure.

For Review Only

Table 2. Distribution and correlation (overlap) of ADRD risk among Dunedin Study members as measured by the four published risk indexes and the DunedinARB.

	CAIDE	LIBRA	LANCET	ANU-ADRI	DunedinARB
CAIDE	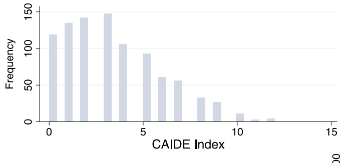				
LIBRA	.55 (.50, .60)	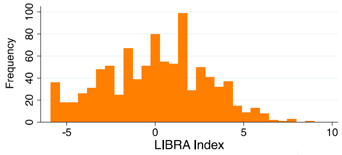			
LANCET	.60 (.54, .65)	.63 (.59, .68)	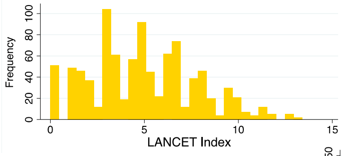		
ANU-ADRI	.66 (.61, .71)	.68 (.64, .73)	.80 (.76, .84)	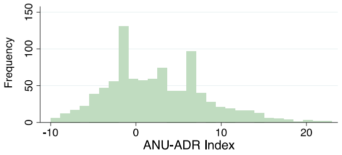	
DunedinARB	.63 (.58, .68)	.64 (.59, .69)	.75 (.71, .80)	.74 (.70, .78)	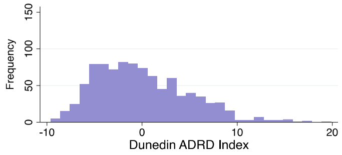

Note. Table cells below the diagonal present pairwise Pearson's r correlation coefficients (95% confidence intervals). All correlations are statistically significant, $p<.001$. Table cells on the diagonal present histograms showing the distribution of each risk index in the Dunedin Study cohort and the DunedinARB benchmark.

For Review Only

Table 3. Associations of the 10 domains of risk comprising the DunedinARB with the four published risk indexes.

	CAIDE (8 risk factors)	LIBRA (12 risk factors)	LANCET (12 risk factors)	ANU-ADRI (15 risk factors)
	Pearson's r			
1. Genetic risk	.35***	-.02	.06	.02
2. Lifestyle risk	.40***	.58***	.58***	.62***
3. Socioeconomic status risk	.56***	.51***	.45***	.56***
4. Psychological & somatic risk	.08*	.29***	.45***	.40***
5. Physical & sensory function risk	.28***	.30***	.50***	.35***
6. Cardio-metabolic risk	.50***	.42***	.37***	.36***
7. Inflammatory risk	.18***	.25***	.25***	.27***
8. Epigenetic aging risk	.23***	.20***	.25***	.24***
9. Harmful events and exposures	.11***	.12***	.24***	.26***
10. Subjective overall health	.38***	.45***	.51***	.51***

Note. Cells highlighted in yellow indicate risk domains specifically represented in the construction of the risk index.
*p-values <.05, **p-values <.01, ***p-values <.001

For Review Only

For Review Only

Table 4. Comparison of the four published risk indexes against the DunedinARB in associations with midlife measures of brain structural and functional integrity, adjusted for sex.

	**Brain structural integrity (MRI) measures**			**Brain functional integrity (cognitive) measures**		
	**brainAGE**	**Log WMH volume**	**Hippocampal volume**	**Objective midlife IQ**	**Subjective midlife cognitive problems**	**IQ change from childhood**
		**β (95% CI)**			**β (95% CI)**	
CAIDE	.16<br>(.08, .23)	.16<br>(.09, .23)	-.09<br>(-.15, -.03)	-.44<br>(-.50, -.37)	.14<br>(.08, .21)	-.18<br>(-.25, -.11)
LIBRA	.17<br>(.10, .24)	.13<br>(.06, .19)	-.08<br>(-.14, -.02)	-.36<br>(-.42, -.30)	.28<br>(.22, .34)	-.16<br>(-.22 -.09)
LANCET	.20<br>(.14, .27)	.14<br>(.07 .20)	-.11<br>(-.17, -.05)	-.39<br>(-.45, -.33)	.32<br>(.26, .38)	-.15<br>(.21, -.09)
ANU-ADRI	.22<br>(.15, .28)	.13<br>(.06, .20)	-.08<br>(-.14, -.02)	-.43<br>(-.49, -.37)	.30<br>(.24, .36)	-.20<br>(-.27, -.14)
DunedinARB	.22<br>(.16, .29)	.19<br>(.13, .26)	-.09<br>(-.15, -.03)	-.49<br>(-.55, -.44)	.38<br>(.32, .44)	-.26<br>(-.33, -.20)

Note. brainAGE = brain Age Gap Estimate, which is the difference between an individual's chronological age at the time of imaging and their "brain age," as estimated by a machine-learning algorithm trained to predict chronological age from MRI measures in independent samples ranging in age from 19 to 82. WMH = white matter hyperintensities.

For Review Only

Supplement

For manuscript entitled, “Improving risk indexes for Alzheimer disease and related dementias for use in midlife.”

List of materials

Table 1. Description of the risk indicators included in the Dunedin ADRD Risk Benchmark and assignment of risk points. 2

Table 2. Description of the midlife brain integrity measures. 16

Table 3. Assignment of risk points and weighting for each risk factor in the four published ADRD indices. 18

Table 4. Correlations among individual risk indicators within the 10 classes of risk comprising the DunedinARB. 24

Table 5. Association of the 10 domains of risk comprising the DunedinARB with the midlife measures of brain integrity. 29

Table 6. Association of the 10 domains of risk comprising the DunedinARB with longitudinal cognitive decline over and above the 4 published risk indices. 30

References....................31

For Review Only

Table 1. Description of the risk indicators included in the Dunedin ADRD Risk Benchmark and assignment of risk points.

Risk domain[a]	Risk indicator	Description	Risk point assignment[b]
Genetic risk[1,2]	Family history of dementia	Study members reported at age 45 on family history of dementia. (N=925 with present data before imputation, 99% of the analytic sample).	Risk points were assigned (0 [92.8% of cohort]; 1 [7.1%]; or 2 [0.1%]) based on number of parents with diagnosed dementia.
	APOE ε4 allele status	Number of APOE ε4 alleles was assessed. APOE protein iosoforms E2/E3/E4 were derived from phased haplotypes of SNPs rs7412 and rs429358 assayed on a genome-wide array, Infinium OmniExpress-12 v1.1 BeadChip array (Illumina Inc., San Diego, California). (N=848 with present data before imputation, 90% of the analytic sample).	Risk points were assigned (0 [69.7%]; 1 [27.2%]; or 2 [3.1%]) based on the number of ε4 alleles present.
Lifestyle risk[3–8]	Physical activity	Study members reported at age 45 on hours per week of leisure-time physical activity at moderate or greater levels of intensity. Cohort range: 0 to 22,	Risk points were assigned based on World Health Organization guidelines for adults aged 18-64 years[9]: -1 = >3hrs weekly (33.8%);

	mean(SD) = 2.82 (3.26). (N=908 with present data before imputation, 97% of the analytic sample).	0 = 1-3hrs weekly (31.0%); 1 = <1hr weekly (35.2%).
Diet	Study members reported at age 45 on their weekly diet. A scale assessing adherence to a Mediterranean-style diet was comprised of Study member report about their typical consumption per week (none, <1, 1-2 times, 3-4 times, most days, or daily) of meat, fish, nuts, beans, fruit, vegetables, sugary drinks, and extra virgin olive oil. Higher scores indicate closer adherence to a healthy diet (higher in fish, nuts, beans, fruit, vegetables, and olive oil, and lower in meat and sugary drinks). Resulting scale was z-scored, cohort range = -2.84 to 2.69; mean(SD) = 0(1). (N=900 with present data before imputation, 96% of the analytic sample).	Risk points were assigned as: -1 = >1SD above the mean (15.8%); 0 = within 1 SD of the mean inclusive (70.9%); 1 = < 1SD below the mean (13.3%).
Tobacco smoking	Study members reported at age 45 on daily tobacco smoking habits. (N=924 with present data before imputation, 99% of the analytic sample).	Risk points were assigned based on classification as non-smokers (0 [47.8%]), former smokers (1

For Review Only

		[30.3%]), and current smokers (2 [22.0%]).
Alcohol consumption	Study members reported at age 45 on weekly alcohol consumption habits. Consistent with published ADRD risk indices[10,11], moderate drinkers were designated as lower risk that non-drinkers or heavy drinkers. (N=938 with present data before imputation, 100% of the analytic sample).	Risk points were assigned based on classification as light drinkers (-1 [40.5%]), non-drinkers (0 [7.3%]), and heavy drinkers (1 [52.2%]). Light drinkers consumed ≤ 7 or 14 drinks per week, respectively for females and males, and reported fewer than 6 occasions of binge drinking (5 or more drinks in a single occasion) in the past year.
Folic acid supplementation	Study members reported at age 45 on whether they regularly took folic acid or B complex supplementation. (N=938 with present data before imputation, 100% of the analytic sample).	Risk points were assigned as: -1 = Yes (1.8%); 0 = No (98.2%).

For Review Only

	Regular prophylactic NSAID use	Study members reported at age 45 on whether they regularly took non-steroidal anti-inflammatory drugs (NSAIDs). (N=938 with present data before imputation, 100% of the analytic sample).	Risk points were assigned as -1 = Yes (20.8%); 0 = No (79.2% of cohort).
Socioeconomic risk[12–14]	Occupational attainment	Study members reported at age 45 on their occupational attainment. Occupations were scored using the New Zealand Socioeconomic Index (NZSEI-06), which codes each occupation based on its associated education level and income in the NZ Census (score range, 10 [low status]-90 [high status]) and groups occupations into 6 status levels. Examples of occupations in the 6 groups include medical practitioner (NZSEI code 90; group 6), engineering professional (code 66; group 5), database administrator (code 59; group 4), personal assistant (code 44; group 3), office cashier (code 28; group 2), and fish filleter (code 23; group 1). Homemakers and others not working in the past year were assigned the status of their most recent occupation, as reported at age 38. Study members who had been out of the labor force since age 32 were assigned the status of their partner if they had	Risk points were assigned as: -1 = High status (groups 5 and 6) (33.2%); 0 = Middle status (groups 3 and 4) (46.2%); 1 = Low status (groups 1 and 2) (20.7%).

For Review Only

		a partner with whom they shared a household. (N=935 with present data before imputation, 99% of the analytic sample).	
	Educational attainment	Study member's reported on their educational attainment by age 45 and were grouped into ranked education levels of: 0 = no certifications (N = 138, 14.7%); 1 = school certification achieved (N = 135, 14.4%); 2 = high school graduate or equivalent (N = 376, 40.1%); and 3 = Bachelor's degree or higher (N = 288, 30.7%). (N=937 with present data before imputation, 99% of the analytic sample).	Risk points were assigned as: -1 = Bachelor's degree or higher (30.7%); 0 = high school graduate or equivalent / school certification (54.6%); 1 = no school certification (14.7%)
Psychological and somatic function risk[15–20]	Pain interference with daily function	Study members reported at age 45 on pain interference with daily life (0=not at all, to 5=very much) via questions about the extent to which pain hinders engagement with social, cognitive, emotional, physical, and recreational activities. Cohort range: 0-20, mean(SD)=3.24(4.24). (N=908 with present data before imputation, 97% of the analytic sample).	Risk points were assigned as: 0 = ≤1 SD above the mean (84.8%); 1 = >1 SD above the mean (15.2%).

For Review Only

History of migraine	Study members reported at each assessment wave from age 26 to 45 about whether they had experienced frequent headaches lasting from 30 min to 7 days in the past year. Headache pain characteristics and symptoms were also assessed and headaches classified as either tensio-type or migraine.[21,22] A count of phases with migraine headaches was created. (N=938 with present data before imputation, 100% of the analytic sample).	Risk points were assigned as: 0 = never met criteria for migraine in adulthood (75.4%); 1 = met criteria for migraine at at least one wave (24.6%). 47.2% of Study members who met criteria for migraine did so at multiple waves.
History of depression	Study members reported at each assessment wave from age 15 to 45 about about symptoms of Major Depressive Disorder over the past year. 50.5% of Study members met criteria for depression at at least one wave and 28.4% met criteria at multiple waves. (N=938 with present data before imputation, 100% of the analytic sample).	Risk points were assigned as: 0 = 1 or fewer episodes of depression across adulthood (71.6%); 1 = >1 episode of depression across adulthood (28.4%).

For Review Only

Loneliness / social isolation	Study members reported at age 45 on loneliness and social isolation via response (0=hardly ever, 1=some of the time, 2=often) to four items adapted from the UCLA Loneliness Scale[23] (e.g., "How often do you feel you lack companionship?"; "How often do you feel isolated from others?"). Items were summed to create a loneliness / social isolation scale. Cohort range: 0-8, mean(SD)=1.21(1.77). (N=922 with present data before imputation, 98% of the analytic sample).	Risk points were assigned as: 0 = ≤1 SD above the mean (80.4%); 1 = >1 SD above the mean (19.6%).
Sleep quality	Study members reported at age 45 on sleep quality using the Pittsburgh Sleep Quality Index (PSQI).[24] The PSQI consists of 18 self-report items relating to individuals' sleep patterns and different forms of sleep impairment in the past month. These questions are used to derive scores for seven different components of sleep (subjective sleep quality, sleep latency, sleep duration, habitual sleep efficiency, sleep disturbances, use of sleep medication and daytime dysfunction), each scored from 0 to 3.	Risk points were assigned as: 0 = ≤1 SD above the mean (85.4%); 1 = >1 SD above the mean (14.6%).

For Review Only

	These were summed to produce a global score ranging from 0 to 21, with higher scores reflecting worse sleep quality. Cohort range: 1-17, mean(SD)=6.24(2.41). (N=909 with present data before imputation, 97% of the analytic sample).	
Neuroticism & Conscientiousness	At the age 45 assessment, informants nominated by the Study members as people "who knew them well" were mailed questionnaires and asked to describe each Study member using a 25-item version of the Big Five Inventory, which measured the personality traits of Neuroticism and Conscientiousness.[25] Items such as "Can be moody" and "Is emotionally stable, not easily upset" asssesed Neuroticism and items such as "Does a thorough job" and "Makes plans and follows through with them" assessed Conscientiousness. Resulting scales ranged from 0 to 10: cohort mean(SD) Neuroticism = 3.96(2.02) and Conscientiousness = 7.48(1.54). (N=883 with present data before imputation, 94% of the analytic sample).	Risk points for Neuroticism were assigned as: 0 = ≤1 SD above the mean (81.2%); 1 = >1 SD above the mean (18.8%). Risk points for Conscientiousness were assigned as: -1 = >1 SD above the mean (14.0%); 0 = ≤1 SD above the mean (86.0%).

For Review Only

Physical and Sensory function risk[26–31]	Balance	Balance was measured at age 45 using the Unipedal Stance Test as the maximum time achieved across three trials of the test with eyes closed.[32] Cohort range: 1-30, mean(SD)=14.58(9.72). (N=911 with present data before imputation, 97% of the analytic sample).	Risk points were assigned as: 0 = ≥1 SD below the mean (86.4%); 1 = <1 SD below the mean (13.6%).
	Gait	Gait speed (meters per second) was assessed at age 45 with the 6-m-long GAITRite Electronic Walkway (CIR Systems, Inc) with 2-m acceleration and 2-m deceleration before and after the walkway, respectively. Gait speed was assessed under 3 walking conditions: usual gait speed (walk at normal pace from a standing start, measured as a mean of 2 walks) and 2 challenge paradigms, dual task gait speed (walk at normal pace while reciting alternate letters of the alphabet out loud, starting with the letter "A," measured as a mean of 2 walks) and maximum gait speed (walk as fast as safely possible, measured as a mean of 3 walks). We calculated the mean of the 3 individual walk	Risk points were assigned as: 0 = ≥1 SD below the mean (84.9 = <1 SD below the mean (15.1%).

For Review Only

	conditions to generate our primary measure of composite gait speed.[33] Cohort range: 0.74-2.12, mean(SD)=1.41(0.19). (N=904 with present data before imputation, 96% of the analytic sample).	
Objective hearing function (hearing acuity)	Hearing thresholds were measured at age 45 by conducting pure-tone audiometry. In a sound-attenuating booth, pure-tones delivered in the following order of frequencies – 1000 Hz, 2000 Hz, 4000 Hz, 8000 Hz, 12500 Hz, and 500 Hz – were presented to the study members through headphones. Presentation intensity levels began at 40 decibels at hearing level (dB HL) for normal hearing study members, and 60 dB HL for hearing aid users. Study members used a response button to indicate whenever they heard a tone, and the lowest intensity level that elicited a response was identified as the hearing threshold for that frequency. A high PTA was calculated by averaging 8000 Hz and 12500 Hz. Results from the best ear are reported. Cohort range: -7.5 to 85, mean(SD)=22.12(14.75).	Risk points were assigned as: 0 = ≥1 SD below the mean (84.2%); 1 = <1 SD below the mean (15.8%).

For Review Only

Subjective hearing function	Study members reported at age 45 on hearing problems via responses to 3 items from the Speech, Spatial, and Qualities of Hearing Scale (SSQ12)[34] (e.g., "Can you follow the conversation in a busy restaurant?"). Cohort range: 0-29, mean(SD)=7.86(5.12). (N=924 with present data before imputation, 99% of the analytic sample).	Risk points were assigned as: 0 = ≤1 SD above the mean (82.2%); 1 = >1 SD above the mean (17.8%).
Objective vision function	Contrast sensitivity was measured at age 45 using a Pelli-Robson chart administered by trained technicians. The chart presents 3 letters per line and the letters gradually fade from black to gray to white on a white background to determine the lowest level of "contrast" that the eye can detect. The resulting measure is a contrast sensitivity score function, reflecting a person's best-corrected contrast detection threshold, the lowest contrast at which a pattern can be seen. Cohort range: 1.4-2.25, mean(SD)=2.00 (0.13). (N=904 with present data before imputation, 96% of the analytic sample).	Risk points were assigned as: 0 = ≥1 SD below the mean (96.1%); 1 = <1 SD below the mean (3.9%).

For Review Only

	Subjective vision function	Study members reported on vision difficulties at age 45 via responses on the 10-item Vision Quality of Life Core Measure (VCM1) questionnaire[35] (e.g., "How often has your eyesight stopped you from doing the things you wanted to do?"). Cohort range: 0-42, mean(SD)= 3.61 (4.70). (N=925 with present data before imputation, 99% of the analytic sample).	Risk points were assigned as: 0 = ≤1 SD above the mean (88.0%); 1 = >1 SD above the mean (12.0%).
	Poor sense of smell	Study members reported at age 45 on poor sense of smell via response to the question, "Have you had problems with your sense of smell, such as not being able to smell things, or things not smelling the way they should" that had lasted for at least 3 months. (N=931 with present data before imputation, 100% of the analytic sample).	Risk points were assigned as: 0 = No (96.8%); 1 = Yes (3.2%).
Cardio-metabolic function risk[36–41]	Blood pressure (hypertension)	Systolic and diastolic blood pressure were assessed at age 45 according to standard protocols with a BpTRU™ Vital Signs Monitor BPM 200. (N=906 with present data before imputation, 97% of the analytic sample).	Risk points were assigned based on classification as non-hypertensive (0 [82.0%]) or hypertensive (1 [18.0%]). Study members were considered hypertensive if they had

For Review Only

		systolic blood pressure 140 mm Hg or greater or diastolic blood pressure 90 mm Hg or greater.
BMI (weight status)	Height was measured at age 45 using a Seca 264 Wireless Stadiometer. Weight was measured at age 45 to the nearest 0.1 kg using calibrated scales. Individuals were weighed in light clothing. Body mass index (BMI) was calculated. Cohort range: 16.17-62.17, mean(SD)=28.45(5.77). (N=920 with present data before imputation, 98% of the analytic sample).	Risk points were assigned based on classification as non-obese (0 = <30BMI, 66.2%) and obese (1 = ≥30BMI, 33.8%).
Diabetes status	Whole blood glycated hemoglobin concentration (A1C) (expressed as a percentage of total hemoglobin) was measured at age 45 by ion exchange high performance liquid chromatography (Variant II: BioRad, Hercultes, Calif.), a method certified by the US National Glycohemoglobin Standardization Program (http://www.ngsp.org/).	Risk points were assigned based on classification as non-diabetic (0 = <6.5% A1C level, 97.7% of the cohort) and diabetic (1 = ≥6.5% A1C level, 2.3% of cohort).

For Review Only

	(N=876 with present data before imputation, 93% of the analytic sample).	
Total cholesterol and triglycerides	Serum non-fasting total cholesterol and triglycerides levels were measured at age 45 by colorimetric assay on a Cobas c702 analyzer. (N=879 with present data before imputation, 94% of the analytic sample).	Risk points were assigned based on clinical thresholds for high cholesterol (1 = ≥6.5 mmol/L, 8.1%) and high triglyceridies (1 = >2.26 mmol/L, 33.2%).
Retinal vascular health	Vascular health was measured at age 45 through assessment of retinal venular vessel calibers. Digital fundus photographs were taken after 15 min of dark adaptation and graded at the SNEC Ocular Reading Centre, Singapore National Eye Centre and Measurements using trained graders following standardized protocols with higher intergrader reliability. Vessel calibers were recorded for arterioles and venules where they passed through a region located 0.50 to 2.00 disk diameters from the optic disk margin and summarized as central retinal artery equivalent (CRAE) and central retinal vein	Risk points were assigned as: 0 = ≥1 SD below the mean (84.1%); 1 = <1 SD below the mean (15.9%).

For Review Only

		equivalent (CRVE).[42] Cohort range -2.71 to 3.72, mean(SD) = 0.01(0.98). (N=890 with present data before imputation, 95% of the analytic sample).	
Inflammatory risk[43–45]	CRP level	Serum C-reactive protein (mg/L) was measured at age 45 using particle-enhanced immunoturbidimetric assays on a Cobas c702 analyzer (Roche Diagnostics GmbH) following standard procedures. The lower detection limit of the assay was 0.3 mg/L. The intraassay and interassay CVs reported by the manufacturer were r = 0.28–1.34% and r = 2.51–5.70%, respectively. Values were log-transformed for analysis to account for positive skew. Cohort range 0-4.51, mean(SD) = 0.97(0.69). (N=879 with present data before imputation, 94% of the analytic sample).	Risk points were assigned as: 0 = ≤1 SD above the mean (85.8%); 1 = >1 SD above the mean (14.2%).
	Il-6 level	Serum IL-6 (pg/mL) was measured at age 45 using an electrochemiluminescence immunoassay on a Cobas e 602 analyzer (Roche Diagnostics GmbH)	Risk points were assigned as: 0 = ≤1 SD above the mean (93.9%); 1 = >1 SD above the mean (6.1%).

For Review Only

	following standard procedure. The lower detection limit of the assay was 1.5 pg/mL. The intraassay and interassay CVs reported by the manufacturer were 2.5–6.0% and 2.9–8.5%, respectively. Cohort range 0.40-28.47, mean(SD) = 2.18(2.54). (N=876 with present data before imputation, 93% of the analytic sample).	
SuPAR level	Plasma suPAR (ng/mL) was measured at age 45 with the suPARnostic AUTO Flex ELISA (ViroGates A/S, Birkerød, Denmark) according to manufacturer's instructions. The detection limit of the assay was 0.1 ng/mL. The intraassay correlation of repeat measurements of the same sample was r = 0.98 and coefficient of variation (CV) = 2.4%, and the interassay correlation was r = 0.81 and CV = 12.8%. Cohort range 0.87-14.37, mean(SD) = 3.07(1.06). (N=875 with present data before imputation, 93% of the analytic sample).	Risk points were assigned as: 0 = ≤1 SD above the mean (89.8%); 1 = >1 SD above the mean (10.2%).

For Review Only

	Rheumatoid arthritis status	Study members reported at age 45 on their history of rheumatoid arthritis during a general health screen. (N=926 with present data before imputation, 99% of the analytic sample).	Risk points were assigned as: 0 = No current arthritis diagnosis (98.2%); 1 = Current arthritis diagnosis (1.8%).
Cellular aging (DNA methylation clock) risk[46,47]	4 separate DNA methylation aging clocks	DNA methylation was measured at age 45 as CpG methylation Beta values derived from leukocyte DNA samples using the EPIC array (Illumina Inc., San Diego, California). Methylation values were transformed into four separate DNA methylation "aging" clocks: Horvath,[48] Hannum,[49] PhenoAge,[50] and Grim.[51] Horvath and Hannum represent first-generation clocks, trained on chronological age in diverse samples. PhenoAge and Grim represent second-generation clocks, trained on phenotypic biomarkers associated with aging (e.g., white blood cell count, albumin levels, etc.). All clocks were calculated using the online calculator found at https://dnamage.genetics.ucla.edu/new. 'Normalization' and 'advanced analysis in blood' options were selected, and data were anonymized prior to upload. From the results file, the corresponding epigenetic age calculations	Risk points were assigned for each DNA methylation clock as: 0 = ≤1 SD above the mean; 1 = >1 SD above the mean.<br>Horvath: 16.4% at risk<br>Hannum: 15.4% at risk<br>PhenoAge: 15.1% at risk<br>Grim: 16.5% at risk

For Review Only

		(DNAmAge, DNAmAgeHannum, DNAmPhenoAge, DNAmGrimAge) were extracted. (N=819 with present data before imputation, 87% of the analytic sample).	
Harmful events and exposures[52–58]	Early life lead exposure	Blood-lead level (ug/dL) was assessed at age 11 years. Approximately 30ml of venous blood was collected and whole blood samples analyzed through graphite furnace atomic absorption spectrophotometry. Details on the method of blood collection, storage, and analysis have been previously described.[59,60] Cohort lead exposure matched that of other same-aged cohorts tested in the United States and United Kingdom.[60] Cohort range: 4-31, mean(SD) = 10.85(3.61). (N=541 with present data before imputation, 58% of the analytic sample).	Risk points were assigned as: 0 = ≤1 SD above the mean (88.7%); 1 = >1 SD above the mean (11.3%).
	Occupational exposure to pesticides	Study members reported at age 45 on their current occupation. (N=877 with present data before imputation, 93% of the analytic sample).	Risk points were assigned based on occupations that include exposure to pesticides, insecticides, fungicides, or timber preservatives (1 point, 9.9% of cohort) or not (0 points, 90.1 %).

For Review Only

	History of traumatic brain injury	Study member history of traumatic head injury was assessed prospectively by asking at each assessment wave if they experienced a head injury requiring medical attention plus aftercare over the past year. Parents reported on head injury at assessment waves in childhood and adolescence and Study members self-reported at adult assessment waves. (N=938 with present data before imputation, 100% of the analytic sample).	Risk points were assigned as: 0 = no history of traumatic head injury by age 45 (88.5%); 1 = history of traumatic head injury (11.5%).
Subjective Overall Health[61,62]	Self-rated health	Study members reported at age 45 on their subjective overall health in response to the question, "In general, would you say your health is: excellent, very good, good, fair, poor." Cohort range: 1-5, mean(SD) = 3.66(0.90). (N=931 with present data before imputation, 99% of the analytic sample).	Risk points were assigned as: -1 = very good to excellent (59.3%); 0 = good (31.7%); 1 = fair to poor (9.1%).
	Informant-rated health	Informants who knew the Study members well rated Study member overall health (excellent, very good, good, fair, poor) on questionnaires mailed to them	Risk points were assigned as: -1 = very good to excellent (67.3%); 0 =

For Review Only

	during the age 45 assessment. Cohort range: 1-5, mean(SD) = 3.79(0.89). (N=883 with present data before imputation, 94% of the analytic sample).	good (25.3%); 1 = fair to poor (7.5%).
Research worker-rated health	Study research workers reported on their subjective impressions of Study member overall health (excellent, very good, good, fair, poor) at the age 45 assessment. Staff ratings were obtained from four raters for each Study member: the cardiovascular nurses, the sensory technicians, the Study Director, and the Assessment Manager (who was in charge of informed consent and logistics on the in-Unit assessment day). Cohort range: 1-5, mean(SD) = 3.36(0.84). (N=934 with present data before imputation, 99% of the analytic sample).	Risk points were assigned as: -1 = very good to excellent (47.7%); 0 = good (39.1%); 1 = fair to poor (13.3%).

Note. Indicators were present for 93-100% of the analytic sample for all indicators except DNA methylation clocks and childhood blood-lead level, which were available for only 87% and 58% of the cohort respectively; missing indicator values were imputed to create complete indicator data for the entire cohort present at the age-45 wave. Removing the lead-level indicator did not change the results.

For Review Only

[a]Representative empirical studies, meta-analyses, systematic reviews, and narrative reviews are cited to describe the evidence of associations of known or proposed risk factors within the 10 risk domains with ADRD. These are cited for expository purposes and are not meant to reflect all the evidence available on a given risk domain, factor, or indicator.

[b]Risk points were assigned after imputation to address missing data. Risk point assignments were not weighted as meta-analyses were not available for all risk indicators. The Dunedin ARI construction allowed each risk domain to contribute equally to the overall risk score.

For Review Only

Table 2. Description of the midlife brain integrity measures.

	Description
**Brain structural integrity measures**	
BrainAGE, years	At age 45, participants completed a neuroimaging magnetic resonance imaging (MRI) protocol including assessment of brain structure with T1-weighted, fluid-attenuated inversion recovery, and diffusion-weighted sequences using a Siemens Skyra 3T scanner (Siemens Healthcare) equipped with a 64-channel head and neck coil.<br><br>High-resolution structural images were used to generate estimates of Study member brainAGE[63], a composite neuroimaging biomarker of age-related brain structure, which estimates aging-related variance in diverse brains structures from cross-sectional brain measures. brainAGE is estimated by training machine-learning algorithms to predict chronological age from structural data collected in large samples of individuals across a broad age range. Using a publicly available algorithm brainAGE was estimated from surface area, cortical thickness, and subcortical volume data to produce a brain-age-gap estimate, which is the difference between an individual's predicted age based on MRI data and their chronological age. Positive brainAGE values suggest that the brain is morphologically "older" than an individual's chronological age would predict.<br><br>Analytic sample N = 869, M (SD) = 0.00 (8.01) years
White Matter Hyperintensities Volume, log mm^3	Total volume of white matter hyperintensities[64] was measured using the Unidentified Bright Objects (UBO) algorithm. White matter hyperintensities are bright spots on a T2 weighted MRI scan that are a sign of deterioration of white matter. The variable was log-transformed to produce an approximately normal distribution.<br><br>Analytic sample N = 852, M (SD) = 6.52 (0.80) log mm^3

For Review Only

Hippocampal Volume, mm^3	Total bilateral volume of the hippocampus[65], a region of the brain important for learning and memory, was measured using FreeSurfer's automated segmentation (ASEG) algorithm.<br><br>Analytic sample N = 861, M (SD) = 86.45 (8.5) mm^3

Brain functional integrity measures

Objective Cognitive Function	Objective cognitive function was assessed using the Wechsler Adult Intelligence Scale–IV (WAIS-IV; potential score range 40-160; standardized in the full cohort to mean[SD]=100[15]) at age 45 years to produce the overall full-scale IQ. Higher scores indicate greater cognitive performance.<br><br>Analytic sample, N = 918, M mean(SD) = 100 (15).
Subjective Cognitive Problems	Subjective everyday cognitive function was reported on by the Study members (self-report) and by individuals who knew the Study members well (informant-report). With matched checklist items, the Study members were interviewed by trained Study staff and the informants were mailed questionnaires. Both were asked about whether the Study members had problems with their memory or attention over the past year, including questions such as whether they were more likely to be "easily distracted" and "get sidetracked" as well as to "misplace wallet, keys or eyeglasses" and "forget to do errands, return calls or pay bills." Self and informant reports were correlated at r=.38, p<.001. Self and informant memory and attention scales were z-scored and averaged to produce an overall subject scale of problems with everyday cognitive function.<br><br>Analytic sample, N = 921, M (SD) = 0.01 (0.77).
Cognitive Decline	Cognitive change (decline) was assessed as change in objective cognitive function from childhood to midlife measured by studying IQ scores at midlife after controlling for IQ scores in childhood

For Review Only

	("residualized change"). The Wechsler Intelligence Scale for Children–Revised (WISC– ; potential score range 40-160; standardized in the full cohort to mean(SD)=100(15)]) yielding the full-scale IQ was administered to each participant at ages 7, 9, and 11 years, with scores from all years averaged together to increase baseline reliability.<br><br>Analytic sample, N = 906, M (SD) = 0.00 (9.55).

Note. SD = standard deviation.

For Review Only

Table 3. Assignment of risk points and weighting for each risk factor in the four published ADRD indices.

1. The CAIDE risk index[66]

Indicator	Level	Risk Points
Age		
	<47 years old	0
	47-53 years old	3
	>53 years old	5
Education		
	≥10 years	0
	7-9 years	3
	0-6 years	4
Sex		
	Female	0
	Male	1
Hypertension		
	SBP ≤ 140 mmHg	0
	SBP >140 mmHg	2
Obesity (BMI)		
	≤30 kg/m2 BMI	0
	>30 kg/m2 BMI	2
Total cholesterol		
	≤6.5 mmol/l total cholesterol	0
	>6.5 mmol/l cholesterol	1
Physical activity		
	Active: physical activity at least twice a week, lasting at least 20–30 min each time, and causing sweating and breathlessness.	0
	Inactive	1

For Review Only

APOE ε4 allele status		
	Non-carrier	0
	Carrier of at least 1 ε4 allele	2
	**Potential Total Points Range**	0 to 18
	**Dunedin Study Total Points Range**	0 to 13

For Review Only

2. The LIBRA risk index[67]

Indicator	Level	Risk Points
Hypertension		
	Not hypertensive	0
	SBP ≥ 140 mmHg or DBP ≥ 90 mmHg	1.6
Obesity (BMI)		
	<30 kg/m2 BMI	0
	≥30 kg/m2 BMI	1.6
High cholesterol		
	<6.5 mmol/l total cholesterol	0
	≥6.5 mmol/l total cholesterol	1.4
Diabetes		
	Not diagnosed	0
	Diagnosed	1.3
Coronary heart disease		
	No disease	0
	Myocardial infarction, angina, ischemic heart disease, or atrial fibrillation reported	1
Chronic kidney disease		
	No disease	0
	Chronic nephritis, chronic renal failure, and proteinuria reported	1.1
Physical inactivity		
	Active: At least 20-30 minutes of daily or 2-3x weekly physical activity causing breathlessness and sweating.	0
	Inactive	1.1
Low/moderate alcohol intake		

For Review Only

	Consumes alcohol once every 2 months or less	-1
	Consumes alcohol once a month or more / Does not drink	0
Smoking		
	Never smoker	0
	Ever smoker	1.5
Depression		
	Did not report feeling somewhat or more "hopeless" on questionnaire	0
	Reported feeling somewhat or more "hopeless" on questionnaire	2.1
Healthy diet		
	Low adherence to a healthy diet (≤8 on a 17 point scale)	0
	high adherence to a healthy diet (>8 on a 17 point scale)	-1.7
Cognitive and social engagement[a†]		
	Self-reported engagement in intellectual and social activities	0
	Self-reported engagement in intellectual and social activities	-3.2
	**Potential Total Points Range**	-5.9 to 12.7
	**Dunedin Study Total Points Range**	-2.7 to 12.7

3. The Lancet Commission[68] risk factor list[b]

Indicator	Level	Risk Points
Education		
	"More education" (Operationalized as: high school graduate or above)	0
	"Less education" (Operationalized as: left school without certification or high school degree)	1.6
Hearing loss		
	No hearing loss or hearing lose with use of a hearing aid	0
	Hearing loss without use of hearing aid	1.9
Traumatic Head Injury		
	No documented history of traumatic head injury	0
	History of traumatic head injury	1.8
Hypertension		
	Not hypertensive	0
	SBP ≥ 140 mmHg or DBP ≥ 90 mmHg	1.6
Excessive alcohol consumption		
	< 21 units alcohol consumed per week	0
	≥ 21 units consumed per week	1.2
Obesity (BMI)		
	<30 kg/m2 BMI	0
	≥30 kg/m2 BMI	1.6
Smoking		
	Non-smoker by midlife	0
	Current smoker by midlife	1.6
Depression		

For Review Only

	Never received a diagnosis by midlife	0
	Diagnosed episode by midlife	1.9
Social isolation		
	Not socially isolated (Operationalized as ≤1 SD above the cohort mean on a scale assessing loneliness)	0
	Socially isolated (Operationalized as >1 SD above the cohort mean on a scale assessing loneliness)	1.6
Physical activity		
	Active	0
	Inactive	1.4
Diabetes		
	No diagnosis	0
	Diagnosed	1.5
Air pollution exposure		
	Low exposure	0
	High exposure[c]	1.1
	**Potential Total Points Range**	0 to 18
	**Dunedin Study Total Points Range**	0 to 18

For Review Only

4. The ANU-ADRI risk index[69]

Indicator	Level	Risk Points
Age for males		
	<65 years	0
	≥65	1 to 38 points
Age for females		
	<65 years	0
	≥65	1 to 41 points
Education		
	> 11 years	0
	8-11 years	3
	<8 years	6
Weight status if age <60		
	Reported as normal	0
	Reported as overweight	2
	Reported as obese	5
Diabetes		
	No diabetes reported	0
	Diabetes reported	3
Symptoms of Depression		
	CES-D score ≤ 16	0
	CES-D score > 16	2
High cholesterol		
	<6.2 mmol/l total cholesterol	0
	≥6.2 mmol/l total cholesterol	3
Traumatic Brain Injury		
	No history of TBI	0
	Positive history of TBI	4
Smoking		
	Never smoker	0
	Ever smoker	1
	Current smoker	4

Review Only

Alcohol intake		
	No alcohol consumed	0
	Light to moderate consumption	-3
	Heavy consumption	0
Social engagement[e]		
	Highest scores in sample	0
	Lowest scores in sample	6
	Low to medium scores in sample	4
	Medium to high scores in sample	1
Physical activity		
	High IPAQ score	0
	Moderate IPAQ score	-2
	Low IPAQ score	-3
Cognitive activity[f†]		
	Lowest cogntive activity questionnaire scores	0
	Middle scores	-7
	Highest scores	-6
Fish intake		
	<.25 servings per week	0
	.25-2 servings per week	-3
	2-4 servings per week	-4
	>4 servings per week	-5
Pesticide exposure		
	Never	0
	Ever	2
	**Potential Total Points Range**	-18 to 35
	**Dunedin Study Total Points Range**	-10 to 23

Note. BMI = Body Mass Index; SBP = Systolic Blood Pressure; DBP = Diastolic Blood Pressure; TBI = Traumatic Brain Injury. CES-D = Center for Epidemiologic Studies Depression scale (CES-D). IPAQ = the International Physical Activity Questionnaire

[†]This measure was not available in the Dunedin Cohort.

[a]Self-reported engagement in intellectual and social activities in the last 12 months (e.g., read the newspaper on a daily basis, have a hobby, take a holiday, using the internet, being a member of any organizations).

[b]At the time of study there were no previous publications assessing risk prediction using the Lancet Comission risk factor list. Lancet Index cut-points were consequently developed based on existing clinical thresholds (e.g., for weight status and hypertension), the presence of categorical conditions (e.g., depression diagnosis), cut-points used by other indices (e.g., for physical activity), and extreme scores on continuous measures relative to cohort peers (e.g., for social isolation).

[c]High air pollution exposure was operationalized as residence for one year or more in a poor air quality city (i.e., top 500 worst air quality cities as ranked by IQAir, www.iqair.com) or a country with average annual outdoor ambiet air quality below World Health Organization standards.

[e]Social engagement in the ANU-ADRI development was measured via a composite score encompassing marital status, size and quality of social networks, level of social activities and living arrangements. It was operationalized in the Dunedin cohort as reverse coded high (≥ 6), medium to high (2.1 to 5), low to medium (0.1 to 2), and low (0) scores on the UCLA Loneliness Scale.[23]

[f]ANU-ADRI utilized a modified form of the Rush Memory and Aging Study cogntive activity questionnaire and assigned risk points based on the distribution of scores within the test sample.

For Review Only

Table 4. Correlations among individual risk indicators within the 10 classes of risk comprising the DunedinARB.

Risk class	Risk factors	Correlations (Pearson's r or Spearman's ρ)					
		1	2				
Genetic risk	1. Family history of dementia	---					
	2. APOE e4 allele status	.10**	---				
		1	2	3	4	5	6
Lifestyle risk	1. Physical activity†	---					
	2. Mediterranean Diet†	.28***	---				
	3. Tobacco smoking	-.11**	-.22***	---			
	4. Alcohol consumption	.04	.05	.15***	---		

For Review O

	5. Folic acid supplementation†	.04	-.03	.02	-.01	---	
	6. Regular prophylactic NSAID use†	.01	-.01	-.02	-.02	.05	---
		**1**	**2**				
Socio-economic risk	1. Occupational attainment†	---					
	2. Educational attainment†	.61***	---				
		**1**	**2**	**3**	**4**	**5**	**6**
Psychosomatic risk	1. Pain interference with daily function	---					
	2. History of migraine	.19***	---				
	3. History of depression	.16***	.23***	---			

Review Only

	4. Social isolation	.12***	.13***	.39***	---		
	5. Poor Sleep quality	.13***	.09**	.20***	.25***	---	
	6. Neuroticism	.07*	.13***	.33***	.28***	.19***	---
	7. Conscientiousness†	-.15***	-.03	-.13***	-.22***	-.15***	-.20***

		1	2	3	4	5	6
Physical and Sensory function	1. Balance†	---					
	2. Gait†	.27***	---				

Review Only

risk	3. Objective hearing function	.11***	.14***	---			
	4. Subjective hearing function†	.12***	.18***	.20***	---		
	5. Objective vision function†	.12***	.12***	.02	.14***	---	
	6. Subjective vision function†	.10***	.11***	.05	.20***	.21***	---
	7. Poor sense of smell	.00	.00	-.07*	-.05	-.06	.00

		1	2	3	4	5	6
Cardio-metabolic status risk	1. Blood pressure (hypertension)	---					
	2. BMI (weight status)	.25***	---				
	3. Diabetes status	.09**	.26***	---			
	4. Cholesterol level	.14***	.11***	.07**	---		

For Review Only

	5. Triglyceride level	.19***	.29***	.28***	.38***	---	
	6. Retinal vascular health†	-.15***	-.13***	-.09*	-.04	-.13***	---
		1	2	3	4		
Inflamma-tory risk	1. C-reactive protein level	---					
	2. Interleukin 6 level	.45***	---				
	3. SuPAR level	.27***	.16***	---			
	4. Rheumatoid arthritis	.05	..04	.06	---		
		1	2	3	4		
Epigenetic cellular aging risk	1. Horvath clock	---					
	2. Hannum clock	.51***	---				
	3. PhenoAge clock	.45***	.43***	---			
	4. Grim clock	.11***	.21***	.37***	---		
		1	2	3			

For Review Only

Harmful events and exposures	Early life lead exposure	---		
	Occupational exposure to pesticides	.04	---	
	History of traumatic head injury	.02	.07*	---
		**1**	**2**	**3**
Subjective Overall Health	Self-rated health†	---		
	Informant-rated health†	.46***	---	
	Research worker-rated health†	.48***	.51***	---

Note: *p-values <.05, **p-values <.01, ***p-values <.001. Correlations calculated using raw, non-dichotomous indicator variables where relevant.
† Indicators where higher scores indicate lower putative ADRD risk (e.g., higher self-rated health indicates better health, higher physical activity indicates greater weekly physical activity).

For Peer Review Only

Table 5. Association of the 10 domains of risk comprising the DunedinARB with the midlife measures of brain integrity.

	Structural integrity (MRI) measures			Functional integrity (cognitive) measures		
	brainAGE	Log WMH volume	Hippocampal volume	Objective midlife IQ	Subjective midlife cognitive problems	IQ change from childhood
	β			β		
**1. Genetic risk**	-.04	.04	.01	-.02	.00	-.02
**2. Lifestyle risk**	.18***	.05	-.08*	-.29***	.14***	-.14***
**3. Social class risk**	.18***	.16***	-.14***	-.60***	.24***	-.30***
**4. Psycho-somatic risk**	.15***	.11**	-.05	-.18***	.45***	-.11***
**5. Physical & sensory risk**	.10**	.12***	-.05	-.37***	.28***	-.18***
**6. Cardio-metabolic risk**	.09*	.15***	-.00	-.10**	.02	-.01
**7. Inflammatory risk**	.11**	.09**	-.03	-.19***	.15***	-.08*
**8. DNA methylation aging risk**	.12***	.08*	-.05	-.20***	.05	-.17***
**9. Harmful events risk**	.03	.02	-.04	-.12***	.13***	-.08*

For Review Only

**10. Subjective health risk**	.15***	.13***	-.07*	-.31***	.35***	-.19***

Note. All associations adjusted for sex. *p-values <.05, **p-values <.01, ***p-values <.001.

For Review Only

Table 6. Association of the 10 domains of risk comprising the DunedinARB with longitudinal cognitive decline over and above the 4 published risk indices, adjusted for sex.

Domain of Risk	Association over & above the CAIDE β	(95% CI)	Association over & above the LIBRA β	(95% CI)	Association over & above the LANCET β	(95% CI)	Association over & above the ANU-ADRI β	(95% CI)
1. Genetic	0.05	(-0.02, 0.12)	-0.02	(-0.08, 0.04)	-0.01	(-0.07, 0.06)	-0.01	(-0.08, 0.05)
2. Lifestyle	**-0.09**	**(-0.16, -0.02)**	-0.08	(-0.16, 0.00)	**-0.08**	**(-0.16, -0.00)**	-0.03	(-0.11, 0.06)
**3. Socioeconomic**	**-0.30**	**(-0.38, -0.23)**	**-0.30**	**(-0.38, -0.23)**	**-0.30**	**(-0.37, -0.23)**	**-0.28**	**(-0.36, -0.21)**
4. Psycho-somatic	**-0.09**	**(-0.16, -0.02)**	-0.07	(-0.14, 0.00)	-0.05	(-0.13, 0.02)	-0.03	(-0.10, 0.04)
**5. Physio-sensory**	**-0.14**	**(-0.21, -0.08)**	**-0.14**	**(-0.21, -0.08)**	**-0.14**	**(-0.21, -0.06)**	**-0.12**	**(-0.19, -0.06)**
6. Cardio-metabolic	**0.09**	**(0.02, 0.17)**	0.07	(-0.00, 0.14)	0.05	(-0.02, 0.13)	**0.07**	**(0.00, 0.14)**
7. Inflammatory	-0.04	(-0.11, 0.03)	-0.04	(-0.11, 0.03)	-0.04	(-0.11, 0.03)	-0.02	(-0.09, 0.05)
**8. Epigenetic**	**-0.14**	**(-0.20, -0.07)**	**-0.14**	**(-0.21, -0.08)**	**-0.14**	**(-0.21, -0.07)**	**-0.13**	**(-0.19, -0.06)**
9. Harmful events	**-0.07**	**(-0.14, -0.01)**	-0.07	(-0.13, 0.00)	-0.05	(-0.12, 0.02)	-0.03	(-0.10, 0.03)
**10. Overall health**	**-0.14**	**(-0.21, -0.07)**	**-0.15**	**(-0.22, -0.08)**	**-0.15**	**(-0.23, -0.08)**	**-0.12**	**(-0.19, -0.04)**

Note. 95% CI = 95% confidence interval. Red highlights indicate risk domains that are significantly associated with cognitive decline over and above the published indices (i.e., in models also including the published risk index).

For Review Only

References

1. Huang W, Qiu C, von Strauss E, Winblad B, Fratiglioni L. APOE genotype, family history of dementia, and Alzheimer disease risk: A 6-year follow-up study. *Arch Neurol*. 2004;61(12):1930-1934. doi:10.1001/archneur.61.12.1930

2. Rawle MJ, Davis D, Bendayan R, Wong A, Kuh D, Richards M. Apolipoprotein-E (Apoe) ε4 and cognitive decline over the adult life course. *Transl Psychiatry*. 2018;8(1):1-8. doi:10.1038/s41398-017-0064-8

3. Scarmeas N, Luchsinger JA, Schupf N, et al. Physical activity, diet, and risk of Alzheimer disease. *JAMA J Am Med Assoc*. 2009;302(6):627-637. doi:10.1001/jama.2009.1144

4. Tyndall AV, Clark CM, Anderson TJ, et al. Protective effects of exercise on cognition and brain health in older adults. *Exerc Sport Sci Rev*. 2018;46(4):215-223. doi:10.1249/JES.0000000000000161

5. Durazzo TC, Mattsson N, Weiner MW. Smoking and increased Alzheimer's disease risk: A review of potential mechanisms. *Alzheimers Dement J Alzheimers Assoc*. 2014;10(3 0):S122-S145. doi:10.1016/j.jalz.2014.04.009

6. Ballarini T, Schröder A, Lent DM van, et al. The effects of Mediterranean diet on memory and Alzheimer's disease biomarkers. *Alzheimers Dement*. 2020;16(S10):e045349. doi:https://doi.org/10.1002/alz.045349

7. Lefèvre-Arbogast S, Féart C, Dartigues JF, Helmer C, Letenneur L, Samieri C. Dietary B vitamins and a 10-year risk of dementia in older persons. *Nutrients*. 2016;8(12):761. doi:10.3390/nu8120761

8. Szekely CA, Breitner JCS, Fitzpatrick AL, et al. NSAID use and dementia risk in the Cardiovascular Health Study: Role of APOE and NSAID type. *Neurology*. 2008;70(1):17-24. doi:10.1212/01.wnl.0000284596.95156.48

For Review Only

9. World Health Organization. *WHO Guidelines on Physical Activity and Sedentary Behaviour.*; 2020.

10. Anstey KJ, Cherbuin N, Herath PM, et al. A self-report risk index to predict occurrence of dementia in three independent cohorts of older adults: The ANU-ADRI. *PLOS ONE.* 2014;9(1):e86141. doi:10.1371/journal.pone.0086141

11. Schiepers OJG, Köhler S, Deckers K, et al. Lifestyle for Brain Health (LIBRA): a new model for dementia prevention. *Int J Geriatr Psychiatry.* 2018;33(1):167-175. doi:10.1002/gps.4700

12. Seblova D, Fischer M, Fors S, et al. Does prolonged education causally affect dementia risk when adult socioeconomic status is not altered? A Swedish natural experiment in 1.3 million individuals. *Am J Epidemiol.* 2021;190(5):817-826. doi:10.1093/aje/kwaa255

13. Russ TC, Stamatakis E, Hamer M, Starr JM, Kivimäki M, Batty GD. Socioeconomic status as a risk factor for dementia death: individual participant meta-analysis of 86 508 men and women from the UK. *Br J Psychiatry.* 2013;203(1):10-17. doi:10.1192/bjp.bp.112.119479

14. Sharp ES, Gatz M. The relationship between education and dementia an updated systematic review. *Alzheimer Dis Assoc Disord.* 2011;25(4):289-304. doi:10.1097/WAD.0b013e318211c83c

15. Low LF, Harrison F, Lackersteen SM. Does personality affect risk for dementia? A systematic review and meta-analysis. *Am J Geriatr Psychiatry.* 2013;21(8):713-728. doi:10.1016/j.jagp.2012.08.004

16. Sabia S, Fayosse A, Dumurgier J, et al. Association of sleep duration in middle and old age with incidence of dementia. *Nat Commun.* 2021;12(1):2289. doi:10.1038/s41467-021-22354-2

17. Zilkens RR, Bruce DG, Duke J, Spilsbury K, Semmens JB. Severe psychiatric disorders in mid-Life and risk of dementia in late-life (Age 65-84 Years): A

For Review Only

population based case-control Study. *Curr Alzheimer Res*. 2014;11(7):681-693. doi:10.2174/1567205011666140812115004

18. Spira AP, Chen-Edinboro LP, Wu MN, Yaffe K. Impact of sleep on the risk of cognitive decline and dementia. *Curr Opin Psychiatry*. 2014;27(6):478-483. doi:10.1097/YCO.0000000000000106

19. Chuang CS, Lin CL, Lin MC, Sung FC, Kao CH. Migraine and risk of dementia: A nationwide retrospective cohort study. *Neuroepidemiology*. 2013;41(3-4):139-145. doi:10.1159/000353559

20. Islamoska S, Hansen ÅM, Wang HX, et al. Mid- to late-life migraine diagnoses and risk of dementia: a national register-based follow-up study. *J Headache Pain*. 2020;21(1):98. doi:10.1186/s10194-020-01166-7

21. Headache Classification Committee of the International Headache Society. Classification and diagnostic criteria for headache disorders, cranial neuralgias and facial pain. *Cephalalgia*. 1988;8 Suppl 7:1-96.

22. Waldie KE, Hausmann M, Milne BJ, Poulton R. Migraine and cognitive function: a life-course study. *Neurology*. 2002;59(6):904-908. doi:10.1212/wnl.59.6.904

23. Russell D, Peplau LA, Cutrona CE. The revised UCLA Loneliness Scale: Concurrent and discriminant validity evidence. *J Pers Soc Psychol*. 1980;39(3):472-480. doi:10.1037/0022-3514.39.3.472

24. Buysse DJ, Reynolds CF, Monk TH, Berman SR, Kupfer DJ. The Pittsburgh Sleep Quality Index: a new instrument for psychiatric practice and research. *Psychiatry Res*. 1989;28(2):193-213. doi:10.1016/0165-1781(89)90047-4

25. Benet-Martínez V, John OP. Los Cinco Grandes across cultures and ethnic groups: multitrait multimethod analyses of the Big Five in Spanish and English. *J Pers Soc Psychol*. 1998;75(3):729-750.

For Review Only

26. Verghese J, Lipton RB, Hall CB, Kuslansky G, Katz MJ, Buschke H. Abnormality of gait as a predictor of non-Alzheimer's dementia. *N Engl J Med*. 2002;347(22):1761-1768. doi:10.1056/NEJMoa020441

27. Thomson RS, Auduong P, Miller AT, Gurgel RK. Hearing loss as a risk factor for dementia: A systematic review. *Laryngoscope Investig Otolaryngol*. 2017;2(2):69-79. doi:10.1002/lio2.65

28. Paik JS, Ha M, Jung YH, et al. Low vision and the risk of dementia: a nationwide population-based cohort study. *Sci Rep*. 2020;10(1):9109. doi:10.1038/s41598-020-66002-z

29. Bathini P, Brai E, Auber LA. Olfactory dysfunction in the pathophysiological continuum of dementia. *Ageing Res Rev*. 2019;55:100956. doi:10.1016/j.arr.2019.100956

30. Verghese J, Wang C, Lipton RB, Holtzer R, Xue X. Quantitative gait dysfunction and risk of cognitive decline and dementia. *J Neurol Neurosurg Psychiatry*. 2007;78(9):929-935. doi:10.1136/jnnp.2006.106914

31. Naël V, Pérès K, Dartigues JF, et al. Vision loss and 12-year risk of dementia in older adults: the 3C cohort study. *Eur J Epidemiol*. 2019;34(2):141-152. doi:10.1007/s10654-018-00478-y

32. Springer BA, Marin R, Cyhan T, Roberts H, Gill NW. Normative values for the unipedal stance test with eyes open and closed. *J Geriatr Phys Ther 2001*. 2007;30(1):8-15. doi:10.1519/00139143-200704000-00003

33. Rasmussen LJH, Caspi A, Ambler A, et al. Association of neurocognitive and physical function with gait speed in midlife. *JAMA Netw Open*. 2019;2(10):e1913123. doi:10.1001/jamanetworkopen.2019.13123

34. Noble W, Jensen NS, Naylor G, Bhullar N, Akeroyd MA. A short form of the Speech, Spatial and Qualities of Hearing scale suitable for clinical use: the SSQ12. *Int J Audiol*. 2013;52(6):409-412. doi:10.3109/14992027.2013.781278

For Review Only

35. Frost NA, Sparrow JM, Durant JS, Donovan JL, Peters TJ, Brookes ST. Development of a questionnaire for measurement of vision-related quality of life. *Ophthalmic Epidemiol*. 1998;5(4):185-210. doi:10.1076/opep.5.4.185.4191

36. Exalto LG, Whitmer RA, Kappele LJ, Biessels GJ. An update on type 2 diabetes, vascular dementia and Alzheimer's disease. *Exp Gerontol*. 2012;47(11):858-864. doi:10.1016/j.exger.2012.07.014

37. Ninomiya T. Epidemiological evidence of the relationship between diabetes and dementia. In: Nakabeppu Y, Ninomiya T, eds. *Diabetes Mellitus: A Risk Factor for Alzheimer's Disease*. Advances in Experimental Medicine and Biology. Springer; 2019:13-25. doi:10.1007/978-981-13-3540-2_2

38. Perrotta M, Lembo G, Carnevale D. Hypertension and dementia: epidemiological and experimental evidence revealing a detrimental relationship. *Int J Mol Sci*. 2016;17(3):347. doi:10.3390/ijms17030347

39. Wartolowska KA, Webb AJS. Midlife blood pressure is associated with the severity of white matter hyperintensities: analysis of the UK Biobank cohort study. *Eur Heart J*. 2021;42(7):750-757. doi:10.1093/eurheartj/ehaa756

40. Cheung CY, Chan VTT, Mok VC, Chen C, Wong TY. Potential retinal biomarkers for dementia: what is new? *Curr Opin Neurol*. 2019;32(1):82-91. doi:10.1097/WCO.0000000000000645

41. Jong FJ de, Schrijvers EMC, Ikram MK, et al. Retinal vascular caliber and risk of dementia: The Rotterdam Study. *Neurology*. 2011;76(9):816-821. doi:10.1212/WNL.0b013e31820e7baa

42. Cheung CY, Tay WT, Mitchell P, et al. Quantitative and qualitative retinal microvascular characteristics and blood pressure. *J Hypertens*. 2011;29(7):1380-1391. doi:10.1097/HJH.0b013e328347266c

43. Peila R, Launer LJ. Inflammation and dementia: epidemiologic evidence. *Acta Neurol Scand Suppl*. 2006;185:102-106. doi:10.1111/j.1600-0404.2006.00693.x

For Review Only

44. Schmidt R, Schmidt H, Curb JD, Masaki K, White LR, Launer LJ. Early inflammation and dementia: a 25-year follow-up of the Honolulu-Asia Aging Study. *Ann Neurol*. 2002;52(2):168-174. doi:10.1002/ana.10265

45. Kinney JW, Bemiller SM, Murtishaw AS, Leisgang AM, Salazar AM, Lamb BT. Inflammation as a central mechanism in Alzheimer's disease. *Alzheimers Dement N Y N*. 2018;4:575-590. doi:10.1016/j.trci.2018.06.014

46. Salameh Y, Bejaoui Y, El Hajj N. DNA methylation biomarkers in aging and age-related diseases. *Front Genet*. 2020;11:171. doi:10.3389/fgene.2020.00171

47. Fransquet PD, Lacaze P, Saffery R, McNeil J, Woods R, Ryan J. Blood DNA methylation as a potential biomarker of dementia: A systematic review. *Alzheimers Dement*. 2018;14(1):81-103. doi:10.1016/j.jalz.2017.10.002

48. Horvath S. DNA methylation age of human tissues and cell types. *Genome Biol*. 2013;14(10):3156. doi:10.1186/gb-2013-14-10-r115

49. Hannum G, Guinney J, Zhao L, et al. Genome-wide methylation profiles reveal quantitative views of human aging rates. *Mol Cell*. 2013;49(2):359-367. doi:10.1016/j.molcel.2012.10.016

50. Levine ME, Lu AT, Quach A, et al. An epigenetic biomarker of aging for lifespan and healthspan. *Aging*. 2018;10(4):573-591. doi:10.18632/aging.101414

51. Lu AT, Quach A, Wilson JG, et al. DNA methylation GrimAge strongly predicts lifespan and healthspan. *Aging*. 2019;11(2):303-327. doi:10.18632/aging.101684

52. Fann JR, Ribe AR, Pedersen HS, et al. Long-term risk of dementia among people with traumatic brain injury in Denmark: a population-based observational cohort study. *Lancet Psychiatry*. 2018;5(5):424-431. doi:10.1016/S2215-0366(18)30065-8

53. Nordström A, Nordström P. Traumatic brain injury and the risk of dementia diagnosis: A nationwide cohort study. *PLOS Med*. 2018;15(1):e1002496. doi:10.1371/journal.pmed.1002496

For Review Only

54. Loef M, Mendoza LF, Walach H. Lead (Pb) and the Risk of Alzheimer's disease or cognitive decline: A systematic review. *Toxin Rev*. 2011;30(4):103-114. doi:10.3109/15569543.2011.624664

55. Schwartz BS, Stewart WF, Bolla KI, et al. Past adult lead exposure is associated with longitudinal decline in cognitive function. *Neurology*. 2000;55(8):1144-1150. doi:10.1212/WNL.55.8.1144

56. Reuben A. Childhood lead exposure and adult neurodegenerative disease. *J Alzheimers Dis*. 2018;64(1):17-42. doi:10.3233/JAD-180267

57. Genuis SJ, Kelln KL. Toxicant exposure and bioaccumulation: A common and potentially reversible cause of cognitive dysfunction and dementia. *Behav Neurol*. 2015;2015:e620143. doi:10.1155/2015/620143

58. Aloizou AM, Siokas V, Vogiatzi C, et al. Pesticides, cognitive functions and dementia: A review. *Toxicol Lett*. 2020;326:31-51. doi:10.1016/j.toxlet.2020.03.005

59. Silva PA, Hughes P, Williams S, Faed JM. Blood lead, intelligence, reading attainment, and behaviour in eleven year old children in Dunedin, New Zealand. *J Child Psychol Psychiatry*. 1988;29(1):43-52.

60. Reuben A, Caspi A, Belsky DW, et al. Association of childhood blood lead levels with cognitive function and socioeconomic status at age 38 years and with IQ change and socioeconomic mobility between childhood and adulthood. *JAMA*. 2017;317(12):1244-1251. doi:10.1001/jama.2017.1712

61. John P, Montgomery P. Does self-rated health predict dementia? *J Geriatr Psychiatry Neurol*. 2013;26(1):41-50. doi:10.1177/0891988713476369

62. Montlahuc C, Soumaré A, Dufouil C, et al. Self-rated health and risk of incident dementia: A community-based elderly cohort, the 3C Study. *Neurology*. 2011;77(15):1457-1464. doi:10.1212/WNL.0b013e31823303e1

For Review Only

63. Elliott ML, Belsky DW, Knodt AR, et al. Brain-age in midlife is associated with accelerated biological aging and cognitive decline in a longitudinal birth cohort. *Mol Psychiatry*. Published online December 10, 2019:1-10. doi:10.1038/s41380-019-0626-7

64. d'Arbeloff T, Elliott ML, Knodt AR, et al. White matter hyperintensities are common in midlife and already associated with cognitive decline. *Brain Commun*. 2019;1(1). doi:10.1093/braincomms/fcz041

65. Elliott ML, Caspi A, Houts RM, et al. Disparities in the pace of biological aging among midlife adults of the same chronological age have implications for future frailty risk and policy. *Nat Aging*. 2021;1(3):295-308. doi:10.1038/s43587-021-00044-4

66. Kivipelto M, Ngandu T, Laatikainen T, Winblad B, Soininen H, Tuomilehto J. Risk score for the prediction of dementia risk in 20 years among middle aged people: a longitudinal, population-based study. *Lancet Neurol*. 2006;5(9):735-741. doi:10.1016/S1474-4422(06)70537-3

67. Vos SJB, van Boxtel MPJ, Schiepers OJG, et al. Modifiable risk Factors for prevention of dementia in midlife, late life and the oldest-old: Validation of the LIBRA index. *J Alzheimers Dis*. 2017;58(2):537-547. doi:10.3233/JAD-161208

68. Livingston G, Huntley J, Sommerlad A, et al. Dementia prevention, intervention, and care: 2020 report of the Lancet Commission. *The Lancet*. 2020;396(10248):413-446. doi:10.1016/S0140-6736(20)30367-6

69. Anstey KJ, Cherbuin N, Herath PM. Development of a new method for assessing global risk of Alzheimer's disease for use in population health approaches to prevention. *Prev Sci*. 2013;14(4):411-421. doi:10.1007/s11121-012-0313-2

For Review Only
