# Supplementary material for: Improving risk indexes for Alzheimer’s disease and related dementias for use in midlife
Source: Brain Commun. 2022 Oct 6;4(5):fcac223. doi: 10.1093/braincomms/fcac223 (PMC9535507; doi:10.1093/braincomms/fcac223)
Supplement: fcac223_Supplementary_Data [file Revision_1_decision_letter.pdf › Revision_1_decision_letter.pdf]

From: brcoms.editorialoffice@oup.com

To: aaron.reuben@duke.edu

CC: alfred.njamnshi@brainafrica.org

BCC:

Subject: Brain Communications - Decision on Manuscript ID BRAINCOM-2022-088.R1

Body: 28-Jun-2022

Dear Mr. Reuben,

It is a pleasure to accept your manuscript entitled "Improving risk indexes for Alzheimer's disease and related dementias for use in midlife" for publication in Brain Communications. Any comments made by the reviewer(s) are included below.

The next step is for your paper to go through “First Look”. We will contact you again shortly if any final files or formatting changes are required for publication or if we have any queries.

If no changes are needed, your submission will be transferred to our Production Department and you will hear from the Production Editor in due course.

VIDEO ABSTRACTS - Brain Communications encourages authors to provide a video abstract to accompany their article. These are hosted on the Brain/Brain Communications YouTube channel. If you are interested in producing a 5-min video abstract to promote your article, then please contact the Editorial Office (brcoms.editorialoffice@oup.com) for further details and guidelines.

PRESS RELEASES: If you are interested in coordinating the timing of any press releases or publicity with the publication of your article it may be useful to agree an embargo date. Please let us know in the Editorial Office (brcoms.editorialoffice@oup.com) as soon as possible so that we can start to put arrangements in place before we send your article to the Production Team.

Thank you very much for your contribution. On behalf of the Editors of Brain Communications, we look forward to your future contributions to the Journal.

Best wishes

Professor Alfred K. Njamnshi
Associate Editor, Brain Communications

Professor Tara Spires-Jones
Founding Editor, Brain Communications

Reviewer(s)' Comments to Author:

Reviewer: 1 Sarah Gregory

Comments to the Author
Thank you to authors for their clear responses to the questions posed and for the additions or edits made to the text. I am satisfied with all responses and would be supportive of this manuscript being accepted for publication in the current form.

Reviewer: 2

Comments to the Author
Thanks to the authors for addressing all the comments. I have no further suggestions.

Date Sent: 28-Jun-2022
