# Supplementary material for: Improving risk indexes for Alzheimer’s disease and related dementias for use in midlife
Source: Brain Commun. 2022 Oct 6;4(5):fcac223. doi: 10.1093/braincomms/fcac223 (PMC9535507; doi:10.1093/braincomms/fcac223)
Supplement: fcac223_Supplementary_Data [file Original_submission_decision_letter.pdf › Original_submission_decision_letter.pdf]

From: brcoms.editorialoffice@oup.com

To: aaron.reuben@duke.edu

CC: alfred.njamnshi@brainafrica.org

BCC:

Subject: Brain Communications - Decision on Manuscript ID BRAINCOM-2022-088

Body: 18-May-2022

Dear Mr. Reuben,

Thank you for your recent submission BRAINCOM-2022-088 entitled "Improving risk indexes for Alzheimer disease and related dementias for use in midlife" to Brain Communications.

Your manuscript has been reviewed, and while the reviewers had largely positive comments, they have some constructive critiques that I think you could address in a minor revision of the manuscript. Therefore, I invite you to respond to the reviewer(s)' comments and revise your manuscript. The reviewer(s)' comments are included at the bottom of this message.

In addition to the reviewers' comments, please also address our Scientific Editor's comments added below to comply with our requirements for presentation of scientific data at Brain Communications.

Please format your revised manuscript according to our Instructions to Authors https://academic.oup.com/braincomms/pages/General_Instructions – a summary of key points is attached to help with this.

To revise your manuscript, click the link below or log into https://mc.manuscriptcentral.com/braincom and enter your Author Centre, where you will find your manuscript title listed under "Manuscripts with Decisions." Under "Actions," click on "Create a Revision." Your manuscript number will be appended to denote a revision.

*** PLEASE NOTE: This is a two-step process. After clicking on the link, you will be directed to a webpage to confirm. ***

https://mc.manuscriptcentral.com/braincom?URL_MASK=08482f7d70a94ee7822c9de9bf35f284

Please upload a revised manuscript with the changes you have made shown either in coloured text or using "track changes".

When submitting your revised manuscript, you will be asked to respond to the reviewer(s) comments. Please be as specific as possible in your response to all of the comments.

The editorial team at Brain Communications also aims to increase transparency in scientific publishing and to demonstrate the value that peer review brings to the scientific process. Please therefore consider whether you are willing to have reviewer comments on your paper and your response published as supplementary data with the paper, if it is accepted. This is entirely optional and will only be done if both the authors and reviewers agree. When submitting your revision you will have the opportunity to update the answer you entered previously.

IMPORTANT: Your original files are available to you when you upload your revised manuscript. Please delete any redundant files before completing the submission.

Because we are trying to facilitate timely publication of manuscripts submitted to Brain Communications, we would appreciate your revised manuscript as soon as possible. If it is not possible for you to resubmit within 3 months, the option to submit a revision will expire, and we would then consider your revised paper as a new submission. However, if you need a little more time, please get in touch and we can extend this deadline for you.

Once again, thank you for submitting your manuscript to Brain Communications and I look forward to receiving your revision.

Best wishes

Professor Alfred K. Njamnshi
Associate Editor, Brain Communications

Professor Tara Spires-Jones
Founding Editor, Brain Communications

Reviewer(s)' Comments to Author: 0

Reviewer: 1 Sarah Gregory

Comments to the Author
General overview summary
Thank you to the authors for the opportunity to read this well written and interesting paper. This paper that will add knowledge to a field that is heavily reliant on risk scores with little external validity outside of their originating cohort. The paper both develops a new, more comprehensive, risk score as well as comparing to four other risk scores, in a mid-life population. The choice of outcomes is sensible (brainAGE, hippocampal volume, white matter hyperintensities, cognition (objective and subjective)).

I had a number of minor comments, mainly requesting clarification around the scoring methodologies in either the manuscript or supplementary materials.

Reviewer comments
Introduction
- The Lancet Commission report is used as one of the indexes, in addition to the LIBRA, CAIDE and ANU-ADRI scores, however there is no background context to this provided where there is description of the other three scores. Could the authors consider adding a brief context here?

Methodology
- Markov-chain Monter Carlo imputation was used for missing data, presumably based on the assumption that all data was missing at random- did the authors consider a sensitivity analysis to test the case that data was missing not at random that may affect the validity of imputation?
- The items selected to create the DunedinARB score were derived from review of literature as well as reviewing 2011 NIH State of the Science Consensus Panel on ADRD risk factors to identify risk factors for inclusion in their score. It would be helpful to include some more information on this process in the manuscript, for example who did the literature search and NIH consensus panel review, how were decisions made about what was and was not included (was there a level of certainty in the evidence before it was included in the scale)?
- I had a number of questions relating to the score creation that the authors could clarify in either the manuscript or supplementary materials to provide additional context for the reader when interpreting the score and determining which, if any, factors they may want to supplement an existing risk score with:
o Family history of dementia- the rates in the Dunedin cohort appear to be very low (7.1% 1 parent, 0.1% 2 parents), particularly when considering the relatively high rates of APOEe4 carriers (27.2% 1 copy, 3.1% 2 copies). Could the authors comment on the representativeness of these two factors in the New Zealand population to provide context for this component? I understand that the participants are likely to have parents reaching dementia onset age now, however the rates still seem very low.
o There are a high rate of smokers included in the cohort (22% current smokers). Is this representative of the wider New Zealand population? There is a reference (Richmond-Rakerd et al, 2020) however I could not see smoking statistics in there, and looking at publicly available rates it seems like a high smoking rate for a majority white cohort.
o There are a high number of participants with no school certification (14.7%), again is this representative of the New Zealand population?
- Was hippocampal volume adjusted for total intracranial volume?
- What statistical tests were used to test for associations between the DunedinARB and outcomes of interest, as well as used to compare the various scores?
- How was the Lancet risk factors score created? I was not clear how each domain was assigned a difference score (e.g. diabetes is 1.5, depression is 1.9).
- In the Lancet risk factors score air pollution contributes and is described, however in the limitations the lack of air pollution data is highlighted. I wondered if air pollution data was included, just not to the level of detail the authors would have wished?

Results & Discussion
- A description of demographics of the cohort would be helpful to set the context of who has been included in this, and therefore who has not to tie into one of the conclusions that this should be replicated in other settings. Some of this information is in the supplementary table describing the score (e.g. education) but some information is not included at all (ethnicity- just that it is majority white).

Reviewer: 2

Comments to the Author
In this paper Reuben et al. leverage the Dunedin cohort - a longitudinal observational birth cohort with subjects aged 45 years to investigate the correlation between alzheimer's disease (AD) risk indices - e.g., CAIDE, LIBRA with imaging derived markers of brain health (such as brain age and hippocampal volume) as well as measures of brain function (e.g., cognitive scores and subjective memory problems). In addition they use the deep phenotyping available in the Dunedin study to define an exhaustive AD risk benchmark comprising ten risk domains to explore correlations between the established risk scores and subscales of the risk dimension. Given the makeup of the birth cohort the risk is explored at 'mid life' and no definite data on later AD and related dementias (ADRD) are available.
Overall, this is a well-written and clear paper with interesting results. I have only few suggestions.

1. Although statistical significance is not the main focus of the work. The presented p-values appear to be uncorrected and statements are made about 'significance' of correlations. I recommend to use an FDR correction to adjust for multiple testing.

2. Given that brain integrity based on imaging data is a main aspect of the paper, the authors could consider writing a more detailed paragraph detailing image acquisition (1.5T or 3T etc.), image processing and biomarker preparation. Most of these details are quite buried in the Supplementary Material at the moment. Moreover, it is not clear whether the hippocampal volume measures were adjusted for total intracranial volume.

Comments from the Scientific Editor:

-Fig2a: please, include a scale in the figure for the heatmap.

-Fig2c: please provide details of the statistical analysis performed, the experimental unit (n= mice, cells, wells, plate downs, etc)) and the number of data-points for each group analysed in these plots, and if the number is <10 show the single data-points. Please note our policy on pseudoreplication, we do not accept analyses where individual cells are the experimental unit when there is likely biological variability is between animals, culture replicates, etc. Please see instructions for authors for more details. Fort his type of data, we require either that a mean/median be taken per animal/culture replicate followed by statistical analyses or that more sophisticated analyses are performed such as mixed effects modelling which can account for random variables.

-Please supply a graphical abstract. A graphical abstract is a visual representation of the central finding or methodology of the paper. It will be displayed in the table of contents. Authors should strive to make the graphical abstract informative, interesting, visually appealing, and straightforward (no caption needed, no abbreviations except gene names). Please upload as a separate high resolution image file (tiff preferred). The technical requirements for the graphical abstracts are:

--size of the submitted image: 1200 pixels square at 300dpi

--font of the text: Arial 12-16 point (smaller fonts will not be legible online)

-Please add an Abbreviated Summary (up to 50 words) of your paper that captures the main purpose and conclusions of the work. The summary should state the main results written in the third person (‘[1st author surname] et al. report that …’) and conclusion. This summary will be used in the contents list online and should not contain any abbreviations other than those on the accepted abbreviations list and accepted gene/protein names, making it accessible to the non-specialist. Please upload it as a Word document. Allowed abbreviations: AIDS; ANOVA; AMPA; ATP; A,T,C,G; CNS; CSF; CT; DNA; ECG; EEG; EMG; GABA; HIV; MRI; NMDA; PET; PCR; RNA.
