# Supplementary material for: Improving risk indexes for Alzheimer’s disease and related dementias for use in midlife
Source: Brain Commun. 2022 Oct 6;4(5):fcac223. doi: 10.1093/braincomms/fcac223 (PMC9535507; doi:10.1093/braincomms/fcac223)
Supplement: fcac223_Supplementary_Data [file Response_to_reviewers_comments_original.pdf › Response_to_reviewers_comments_original.pdf]

1 June 2022

Dear Dr. Njamnshi,

We are submitting a revised version of manuscript BRAINCOM-2022-088, "Improving risk indexes for Alzheimer disease and related dementias for use in midlife."

Thank you for obtaining helpful and constructive reviews of our manuscript, and for your detailed comments. Below we paste the Reviewers' comments (in full), and our responses to each comment (in bold).

We would be pleased to clarify and address any further issues.

Thank you for your consideration.

Yours,

Aaron Reuben
Terrie Moffitt

Note to the Editor:

Both Reviewer #1 and #2 asked for more information about how well the Dunedin Cohort represents the general population. This question has two aspects. First, how well does the cohort represent its home country of New Zealand? We routinely compare the cohort to NZ national statistics, and these comparisons confirm that prevalence rates of study measures match the wider population very closely (as detailed in response to Reviewer #1 below and across the Supplement, Table 1). Second, can data from this cohort generalize to other countries such as the USA or UK? Again, we find that the prevalence rates of study measures match those of other countries quite well. Notably, however, reviewers often comment that prevalence rates seem higher in the Dunedin Cohort than what they are used to seeing in US and UK surveys (e.g., for tobacco smoking). The primary reason for this is that most US and UK surveys are characterized by healthy-volunteer bias and attrition bias (resulting in underestimates of health and social problems). In contrast, the Dunedin cohort is not characterized by such biases, as we have managed to achieve data collection phase retention rates >95% across the last three decades. This leads to a more precise, and somewhat higher, estimate of health and social problems. We explain in greater detail below in response to each query.

REVIEWER # 1

Thank you to the authors for the opportunity to read this well written and interesting paper. This paper will add knowledge to a field that is heavily reliant on risk scores with little external validity outside of their originating cohort. The paper both develops a new, more comprehensive, risk score as well as comparing to four other risk scores, in a mid-life population. The choice of outcomes is sensible (brainAGE, hippocampal volume, white matter hyperintensities, cognition (objective and subjective)).

I had a number of minor comments, mainly requesting clarification around the scoring methodologies in either the manuscript or supplementary materials.

Reviewer comments

Introduction

The Lancet Commission report is used as one of the indexes, in addition to the LIBRA, CAIDE and ANU-ADRI scores, however there is no background context to this provided where there is description of the other three scores. Could the authors consider adding a brief context here?

Thank you for this excellent suggestion. Brief context is now provided for readers, on page 5, on the Lancet Commission risk factors, of equal detail to that provided for the other three indexes. The text now notes that the index is "comprised of the modifiable risk factors for dementia selected by the Lancet Commission on Dementia Prevention, Intervention, and Care identified based on the availability of systematic or meta-analytic review evidence."

Methodology

Markov-chain Monte Carlo imputation was used for missing data, presumably based on the assumption that all data was missing at random- did the authors consider a sensitivity analysis to test the case that data was missing not at random that may affect the validity of imputation?

Thank you for this comment. The large number of risk indicators (48) precluded testing missing-not-at-random for each separate indicator. Notably, risk indicators were present for 93-100% of the analytic sample for all indicators except DNA methylation clocks and childhood blood-lead level, which were available for only 87% and 58% of analytic sample, respectively. DNA methylation clocks were derived from methylation profiles generated in European samples and were therefore created only for Study members of European ancestry. Thus, lack of present data on the DNA methylation clocks largely represents exclusion, not missingness. Childhood blood-lead levels, on the other hand, are known to be missing not-at-random in this cohort, as previously published (Reuben et al., 2017): data is only present for Study members who attended the assessment phase at the research unit at age 11 years and freely agreed to give blood. 579 of the 803 children (72%) in attendance at the unit agreed to give blood. An additional 122 children who lived outside city limits were assessed in their schools, where blood could not be drawn. For these reasons sensitivity tests were run excluding the lead-level indicator from the DunedinARB. This did not change the results. These details are provided for the reader on page 15 of the Supplement.

In contrast to the risk indicator variables, outcome measures were not imputed. Tests of attrition and missingness at the outcome age (45 years) assessment are detailed for readers on page 7 (Statistical Analysis section) and now in a new expanded section in the Supplement (Supplementary Appendix 1: Age-45 Data Collection Phase attrition analysis). The text on page 7 reads: "Attrition analysis (Supplementary Appendix 1) using childhood IQ, childhood SES, and Adverse Childhood Events (ACEs), among other measures, identified no significant differences between the full cohort (N=1,037),

those alive at age 45 (N=997), and those who attended data collection (N=938). Those who were deceased by the time of data collection had significantly lower childhood IQ's than those who were still alive (t=2.09, *p*-value=0.04)."

Reference:

- **Reuben A, Caspi A, Belsky DW, et al. Association of childhood blood lead levels with cognitive function and socioeconomic status at age 38 years and with IQ change and socioeconomic mobility between childhood and adulthood. *JAMA*. 2017;317(12):1244-1251. doi:10.1001/jama.2017.1712**

The items selected to create the DunedinARB score were derived from review of literature as well as reviewing 2011 NIH State of the Science Consensus Panel on ADRD risk factors to identify risk factors for inclusion in their score. It would be helpful to include some more information on this process in the manuscript, for example who did the literature search and NIH consensus panel review, how were decisions made about what was and was not included (was there a level of certainty in the evidence before it was included in the scale)?

Thank you for the opportunity to provide more detail on the risk factor search methodology. The text has been amended to respond to this comment through the inclusion of a new Appendix (Appendix 1: Methodology for the identification of ADRD risk factors for the DunedinARB) in the Supplement, which details the risk factor search methodology. Appendix 1 is now referenced in relevant sections of the text, including on page 8 of the Results section.

The new appendix reads as follows (citations included as superscript numbers for ease of reading):

> **Established and proposed risk factors for ADRD were selected for inclusion in the Dunedin ADRD Risk Benchmark (DunedinARB) following two steps. First, risk factors were selected based on their identification as risk factors through comprehensive review by: 1) the 2011 NIH State of the Science Consensus Panel on ADRD risk factors,[1] 2) the Lancet Commission on Dementia Prevention, Intervention, and Care,[2] and 3) the published ADRD risk indexes included in the comparison stage of the present study (the CAIDE,[3–5] the LIBRA,[6–8] and the ANU-ADRI[9,10]). Second, additional risk factors were identified through two separate non-systematic searches of PubMed, Scopus, and Google Scholar by the senior author (TEM) and first author (AR) for these five key words: Alzheimer's, dementia, risk, review, meta-analysis. These searches were intended to cast the widest net possible and risk factors were selected for inclusion in the DunedinARB if at least two quality studies reported statistically significant evidence of an association with dementia diagnoses in two separate samples, ideally from two separate populations. For the vast majority of risk indicators selected for the DunedinARB through the second search step, the authors found more than two studies reporting a significant risk-factor-dementia association. For example, the personality traits of conscientiousness and neuroticism, which have yet to be included in any ADRD risk index or consensus summary, have been associated with dementia risk in 8-12 different samples, with "robust evidence" identified in two separate systematic review and meta-analyses.[11,12]**

References:

1. **Daviglus ML, Plassman BL, Pirzada A, et al. Risk factors and preventive interventions for Alzheimer disease: State of the science. *Arch Neurol.* 2011;68(9):1185-1190. doi:10.1001/archneurol.2011.100**
2. **Livingston G, Huntley J, Sommerlad A, et al. Dementia prevention, intervention, and care: 2020 report of the Lancet Commission. *The Lancet.* 2020;396(10248):413-446. doi:10.1016/S0140-6736(20)30367-6**
3. **Vuorinen M, Spulber G, Damangir S, et al. Midlife CAIDE dementia risk score and dementia-related brain changes up to 30 years later on Magnetic Resonance imaging. *J Alzheimers Dis.* 2015;44(1):93-101. doi:10.3233/JAD-140924**
4. **Exalto LG, Quesenberry CP, Barnes D, Kivipelto M, Biessels GJ, Whitmer RA. Midlife risk score for the prediction of dementia four decades later. *Alzheimers Dement.* 2014;10(5):562-570. doi:10.1016/j.jalz.2013.05.1772**
5. **Kivipelto M, Ngandu T, Laatikainen T, Winblad B, Soininen H, Tuomilehto J. Risk score for the prediction of dementia risk in 20 years among middle aged people: a longitudinal, population-based study. *Lancet Neurol.* 2006;5(9):735-741. doi:10.1016/S1474-4422(06)70537-3**
6. **Vos SJB, van Boxtel MPJ, Schiepers OJG, et al. Modifiable risk Factors for prevention of dementia in midlife, late life and the oldest-old: Validation of the LIBRA index. *J Alzheimers Dis.* 2017;58(2):537-547. doi:10.3233/JAD-161208**
7. **Schiepers OJG, Köhler S, Deckers K, et al. Lifestyle for Brain Health (LIBRA): a new model for dementia prevention. *Int J Geriatr Psychiatry.* 2018;33(1):167-175. doi:10.1002/gps.4700**
8. **Deckers K, van Boxtel MPJ, Schiepers OJG, et al. Target risk factors for dementia prevention: a systematic review and Delphi consensus study on the evidence from observational studies. *Int J Geriatr Psychiatry.* 2015;30(3):234-246. doi:10.1002/gps.4245**
9. **Anstey KJ, Cherbuin N, Herath PM, et al. A self-report risk index to predict occurrence of dementia in three independent cohorts of older adults: The ANU-ADRI. *PLOS ONE.* 2014;9(1):e86141. doi:10.1371/journal.pone.0086141**
10. **Anstey KJ, Cherbuin N, Herath PM. Development of a new method for assessing global risk of Alzheimer's disease for use in population health approaches to prevention. *Prev Sci.* 2013;14(4):411-421. doi:10.1007/s11121-012-0313-2**
11. **Low LF, Harrison F, Lackersteen SM. Does personality affect risk for dementia? A systematic review and meta-analysis. *Am J Geriatr Psychiatry.* 2013;21(8):713-728. doi:10.1016/j.jagp.2012.08.004**
12. **Aschwanden D, Strickhouser JE, Luchetti M, Stephan Y, Sutin AR, Terracciano A. Is personality associated with dementia risk? A meta-analytic investigation. *Ageing Res Rev.* 2021;67:101269. doi:10.1016/j.arr.2021.101269**

I had a number of questions relating to the score creation that the authors could clarify in either the manuscript or supplementary materials to provide additional context for the reader when interpreting the score and determining which, if any, factors they may want to supplement an existing risk score with:

- Family history of dementia- the rates in the Dunedin cohort appear to be very low (7.1% 1 parent, 0.1% 2 parents), particularly when considering the relatively high rates of

APOEe4 carriers (27.2% 1 copy, 3.1% 2 copies). Could the authors comment on the representativeness of these two factors in the New Zealand population to provide context for this component? I understand that the participants are likely to have parents reaching dementia onset age now, however the rates still seem very low.

Thank you for this two-part question on the representativeness of the Dunedin Cohort to the New Zealand population and the New Zealand population to global averages. First, rates of parental dementia in the Dunedin cohort match the New Zealand population and global trends. In our raw, unimputed measure capturing parental dementia by Study member age 45: 834 Study members had 0 parents with dementia (92.5%), 67 had 1 parent with dementia (7.4%), and 1 had 2 parents with dementia (0.1%). This equates to a dementia prevalence of 3.7% (67 cases out of 1804 parents). The average age of Study member parents at the age 45 assessment was approximately 70 years (mean for mothers = 69.6, SD = 7.1; mean for fathers = 70.6, SD = 8.6). In New Zealand the prevalence of dementia of any kind for adults aged 70-74 years is approximately 2.0% when identified using hospital records (Walesby et al., 2020), which likely under-counts the true dementia prevalence. In the United States the estimated prevalence of dementia in this age-group ranged from 3.0% to 4.1% during our data collection window (Freedman, Cornman, & Kasper, 2021). Thus, Dunedin Study members in our analytic sample had typical rates of parental dementia. (Note that Supplementary Table 1 reports the prevalence rates using the imputed version of the parental dementia variable, which returns slightly lower rates than those reported above).

Second, the rates of APOE ε4 allele carriers in the Dunedin Study match global trends: 27.3% of the cohort carry at least one ε4 allele (27.2% of the analytic sample), which results in an ε4 frequency of 17.0%. This prevalence is typical for people of European descent, is somewhat smaller than what is typical for people of African descent, and is somewhat larger than what is typical for people of Asian descent (Kern et al., 2015; AlzGene meta-analysis for APOE, 2010). We are also very confident in our allele classifications as we have genotyped APOE SNPs twice, once as a candidate marker and then again on a SNP array. The call rate (0. 0.991) and concordance (0.998) was excellent for this genotyping.

These details have now been added to the relevant text in the Supplement, on page 3, Supplementary Table 1, Column 4.

References:

- **Freedman VA, Cornman JC, Kasper JD. National Health and Aging Trends Study Trends Chart Book: Key Trends, Measures and Detailed Tables. 2021. Available at: micda.isr.umich.edu/research/nhats-trends-dashboards.**
- **Kern S, Mehlig K, Kern K, et al. The distribution of Apolipoprotein E genotype over the adult lifespan and in relation to country of birth. *Am. J. Epi.* 2015;181(3):214-217. doi.org/10.1093/aje/kwu442**
- **AlzGene meta-analysis for APOE (e2/3/4). 2010. Available at: http://www.alzgene.org/meta.asp?geneID=83. Accessed May 20, 2022.**

- There are high rate of smokers included in the cohort (22% current smokers). Is this representative of the wider New Zealand population? There is a reference (Richmond-

Rakerd et al, 2020) however I could not see smoking statistics in there, and looking at publicly available rates it seems like a high smoking rate for a majority white cohort.

Thank you for this question. The prevalence of regular tobacco smoking in the Dunedin Cohort is comparable to that of the wider New Zealand population. The New Zealand Health Survey 2016/2017, which occurred within the 2-year span of the Dunedin age 45 assessment phase, identified 20% of the 35-44-year-old population of New Zealand as regular smokers (NZ Health Survey online statistics: https://minhealthnz.shinyapps.io/nz-health-survey-2020-21-annual-data-explorer/_w_4315dbc7/#!/explore-indicators). As Dunedin Study response/retention rates (94% at phase 45) exceed those of national surveys, we tend to capture a greater portion of low SES individuals and thus can be expected to show slightly greater rates of health behaviors negatively correlated with socioeconomic status, such as cigarette smoking.

In general, New Zealand tends to be an ideal study site for investigating health behaviors and health effects of regular tobacco consumption as New Zealanders typically fall in the middle of the global tobacco-consumption curve relative to other populations. In 2019, approximately 12.5% of New Zealanders above the age of 15 years were regular smokers -far less than in high-consumption countries (>25% regular smoking prevalence above age 15 years, e.g., in Turkey and Indonesia) and far more than low-consumption countries (<10% prevalence above age 15 years, e.g., in Costa Rica and Iceland). (OECD Health Statistics: https://www.oecd-ilibrary.org/sites/611b5b35-en/index.html?itemId=/content/component/611b5b35-en)

The correct reference for Dunedin cohort population-representativeness on health measures (Poulton et al., 2015) has been provided on page 6 (Methods: Study Design and Population).

Reference:

- **Poulton R, Moffitt TE, Silva PA. The Dunedin Multidisciplinary Health and Development Study: overview of the first 40 years, with an eye to the future.** ***Soc Psychiatry Psychiatr Epidemiol*****. 2015;50(5):679-693. doi:10.1007/s00127-015-1048-8**

- There are a high number of participants with no school certification (14.7%), again is this representative of the New Zealand population?

Yes. 14.7% of the Dunedin cohort analytic sample for this study (and 14.9% in the full cohort) left secondary school early without qualifications. This exactly matches the prevalence of leaving school without qualifications in the wider New Zealand population, which is 14.7% for those individuals born in the same years as the Dunedin Study members, as determined by the 2020 New Zealand Census. Further details are available in Richmond-Rakerd et al., 2021, Supplement pages 6-7, which is now included in the reference list for this variable.

Reference:

- **Richmond-Rakerd LS, D'Souza S, Andersen SH, et al. Clustering of health, crime and social-welfare inequality in 4 million citizens from two nations. *Nat Hum Behav*. 2020:1-10. doi:10.1038/s41562-019-0810-4**

Was hippocampal volume adjusted for total intracranial volume?

It was not. It has not been our lab's standard procedure to adjust association tests of hippocampal volume for total intracranial volume. We reserve this control for specific occasions because analyses including a global covariate assume that the relative amount of brain matter in a given region is what drives differences in other phenotypes of interest, while analyses that do not include a global covariate assume that the absolute amount is more important. It remains to be empirically demonstrated on a case-by-case basis which of these assumptions more closely reflects the underlying biological mechanisms of interest, and we tend to think that the absolute amount of brain matter in a given region is often the more plausible assumption for a given questions. For this particular research project we were primarily interested in associations of ADRD risk factors with the total volume of the hippocampus, rather than the volume relative to the whole brain. (Issues of collinearity can also make it difficult to interpret changes in association sizes after adjustment for brain size). (Hariri Lab Statement on the use of global covariates in analyses of regional measures of brain structure: haririlab.com/methods/GlobalCovariates.html).

Nevertheless, we appreciate that in this case readers may be interested in such a sensitivity test given the tendency of Alzheimer's-specific pathological processes to selectively target the hippocampus (and thus we would be interested in knowing whether a specific, differential association exists between ADRD risk factors and hippocampus volume over and above total brain size). We have now re-run all tests of hippocampal volume including additional adjustment for total brain volume (which correlates with intracranial volume r>.9). In these models the associations of the risk indexes with hippocampal volume were reduced in size and no longer statistically significant, suggesting that the modest associations of the midlife risk indexes with hippocampal volume were non-specific and likely represented an overall trend of smaller brain volumes among individuals at greater midlife risk for ADRD. These post hoc sensitivity tests, their results and interpretation, have now been added to the Methods, Results, and Discussion on pages 8, 10, 13, and 16.

What statistical tests were used to test for associations between the DunedinARB and outcomes of interest, as well as used to compare the various scores?

The primary tests of the association of the DunedinARB and the other risk indexes with the outcomes of interest were modeled using ordinary least squares (OLS) regression adjusted for sex (regressing the outcomes onto the risk indexes separately, one at a time). This is detailed for readers on page 7 (Statistical Analysis section). Comparison of outcome-associations among the risk indexes was accomplished through formal tests of differences between dependent correlations (Steiger, 1980). This is detailed for readers on page 15 (Results section). Further post hoc sensitivity tests utilized OLS regression to investigate unique associations of the DunedinARB with the longitudinal outcome measure of cognitive decline from childhood. In these models, residualized cognitive decline was regressed onto the DunedinARB while including each of the four published risk indexes as covariates, in turn, to determine whether the DunedinARB was a

significant predictor of cognitive decline over and above each published risk index (4 tests in total). These tests are detailed for readers on page 16 of the Results section.

Reference:

- **Steiger JH. Tests for comparing elements of a correlation matrix. *Psychol Bull.* 1980;87(2):245-251. doi:10.1037/0033-2909.87.2.245**

How was the Lancet risk factors score created? I was not clear how each domain was assigned a difference score (e.g. diabetes is 1.5, depression is 1.9).

Thank you for identifying this omission. These details have now been added to the relevant sections of the Supplement on page 23. The text now states: "Risk scores were assigned based on the Lancet Commission's published 'relative risk for dementia' scores for each risk indicator (Table 1, Column 1 in Livingston et al., 2020)."

Reference:

- **Livingston G, Huntley J, Sommerlad A, et al. Dementia prevention, intervention, and care: 2020 report of the Lancet Commission. *The Lancet.* 2020;396(10248):413-446. doi:10.1016/S0140-6736(20)30367-6**

In the Lancet risk factors score air pollution contributes and is described, however in the limitations the lack of air pollution data is highlighted. I wondered if air pollution data was included, just not to the level of detail the authors would have wished?

Thank you for this comment. We included a measure of air pollution exposure in the creation of the Lancet Index to follow their published indicator list as closely as possible. However, this measure was extremely low resolution and did not allow for fine discrimination of pollution exposure among Study members. For this reason, we did not include the air pollution measure in the DunedinARB, although we hope to do so in future iterations of the Dunedin index once higher resolution air pollution exposure measures become available for the cohort. High air pollution exposure was operationalized as residence for one year or more in a poor air quality city (i.e., top 500 worst air quality cities as ranked by IQAir, www.iqair.com) or a country with average annual outdoor ambient air quality below World Health Organization standards. 11 Study members (1.2%) met these qualifications.

Results & Discussion

A description of demographics of the cohort would be helpful to set the context of who has been included in this, and therefore who has not to tie into one of the conclusions that this should be replicated in other settings. Some of this information is in the supplementary table describing the score (e.g. education) but some information is not included at all (ethnicity- just that it is majority white).

Thank you for this question. Demographics on the Dunedin Cohort are provided on page 6 (Methods: Study Design and Population) and throughout the Supplement, most notably Supplementary Table 1 (Description of the risk indicators included in the DunedinARB). In response to this comment and to those above we have added additional demographic details (and details on the population-representativeness of the cohort) related to dementia prevalence, genetics, and health behaviors to the Methods

and the Supplement. Notably, we have also added race/ethnicity descriptions that note: "The cohort is primarily white (92.5%) (as self-described using fixed categories); 7.5% self-identify as being Māori, which matches the ethnic distribution of the South Island of New Zealand."

REVIEWER # 2

In this paper Reuben et al. leverage the Dunedin cohort - a longitudinal observational birth cohort with subjects aged 45 years to investigate the correlation between Alzheimer's disease (AD) risk indices - e.g., CAIDE, LIBRA - with imaging derived markers of brain health (such as brain age and hippocampal volume) as well as measures of brain function (e.g., cognitive scores and subjective memory problems). In addition they use the deep phenotyping available in the Dunedin study to define an exhaustive AD risk benchmark comprising ten risk domains to explore correlations between the established risk scores and subscales of the risk dimension. Given the makeup of the birth cohort the risk is explored at 'mid life' and no definite data on later AD and related dementias (ADRD) are available. Overall, this is a well-written and clear paper with interesting results. I have only few suggestions.

1. Although statistical significance is not the main focus of the work. The presented p-values appear to be uncorrected and statements are made about 'significance' of correlations. I recommend to use an FDR correction to adjust for multiple testing.

We have now applied the FDR correction to the p-values for all of the tests of the associations of the ADRD risk indices with the brain structure and function outcomes. The results did not change. The text has been amended on pages 7 of the Statistical Analysis section and throughout the Results to reflect this.

2. Given that brain integrity based on imaging data is a main aspect of the paper, the authors could consider writing a more detailed paragraph detailing image acquisition (1.5T or 3T etc.), image processing and biomarker preparation. Most of these details are quite buried in the Supplementary Material at the moment. Moreover, it is not clear whether the hippocampal volume measures were adjusted for total intracranial volume.

We welcome the opportunity to add additional detail on the study measures in the main manuscript. Readers wishing to know the indicators of risk captured in each risk domain in the Dunedin ADRD Risk Benchmark may find this information in the Introduction on pages 4 and 5. Greater detail on the outcome measures is now included in the Methods on pages 6 and 7, with specific additional details on the image acquisition protocol, including that assessment of brain structure included T1-weighted, fluid-attenuated inversion recovery, and diffusion-weighted sequences using a Siemens Skyra 3T scanner (Siemens Healthcare) equipped with a 64-channel head and neck coil. Additional details on the outcome measures are described on page 10 of the Results. Finally, additional details on the adjustment of the hippocampal volume tests for total brain volume (which correlates with total intracranial volume r>.9) have now also been added (as detailed in response to Reviewer #1) on page 8, 10, 13, and 16.

COMMENTS FROM THE SCIENTIFIC EDITOR

Fig2a: please, include a scale in the figure for the heatmap.

Done.

Fig2c: please provide details of the statistical analysis performed, the experimental unit (n= mice, cells, wells, plate downs, etc)) and the number of data-points for each group analysed in these plots, and if the number is <10 show the single data-points. Please note our policy on pseudoreplication, we do not accept analyses where individual cells are the experimental unit when there is likely biological variability is between animals, culture replicates, etc. Please see instructions for authors for more details. For this type of data, we require either that a mean/median be taken per animal/culture replicate followed by statistical analyses or that more sophisticated analyses are performed such as mixed effects modelling which can account for random variables.

Done.

Please supply a graphical abstract. A graphical abstract is a visual representation of the central finding or methodology of the paper. It will be displayed in the table of contents. Authors should strive to make the graphical abstract informative, interesting, visually appealing, and straightforward (no caption needed, no abbreviations except gene names). Please upload as a separate high resolution image file (tiff preferred). The technical requirements for the graphical abstracts are:

--size of the submitted image: 1200 pixels square at 300dpi

--font of the text: Arial 12-16 point (smaller fonts will not be legible online)

Done.

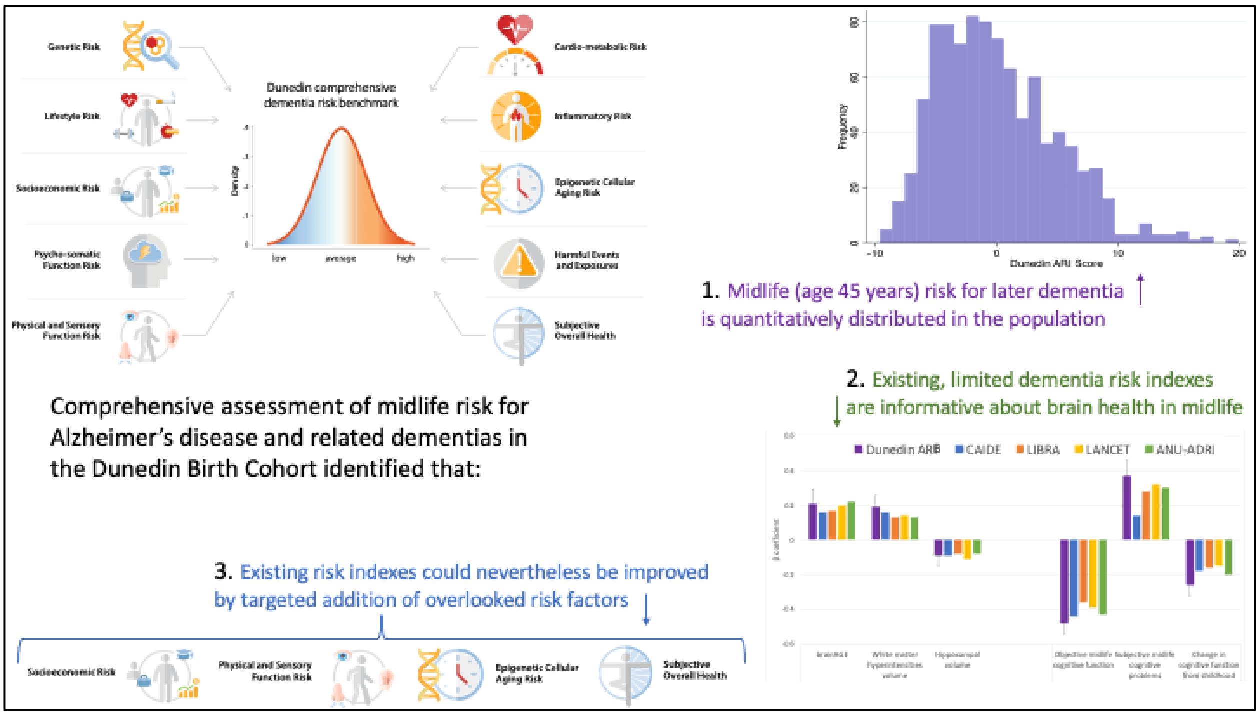

Please add an Abbreviated Summary (up to 50 words) of your paper that captures the main purpose and conclusions of the work. The summary should state the main results written in the third person ('[1st author surname] et al. report that …') and conclusion. This summary will be used in the contents list online and should not contain any abbreviations other than those on the accepted abbreviations list and accepted gene/protein names, making it accessible to the non-specialist. Please upload it as a Word document. Allowed abbreviations: AIDS; ANOVA; AMPA; ATP; A,T,C,G; CNS; CSF; CT; DNA; ECG; EEG; EMG; GABA; HIV; MRI; NMDA; PET; PCR; RNA.

Done. The Abbreviated Summary reads:
"Reuben et al. report that existing dementia-risk indexes are informative about brain health in the general population decades before dementia symptoms emerge, even when the indexes include only a subset of potential risks. Comparisons to a novel comprehensive risk-benchmark indicated that targeted risk-factor additions could improve the selection of at-risk
